# Smart microdevices for biomedical drug delivery: endogenous stimuli as the key to safer therapeutics

**DOI:** 10.1039/d5ra09767c

**Published:** 2026-03-18

**Authors:** Deepanjan Datta, Viola Colaco, Maria Nison, Ananya Prabha H, Sony Priyanka Bandi, Namdev Dhas, Vasudev R Pai, Praveen Halagali, Vamshi Krishna Tippavajhala, Sudarshan Singh, Lalitkumar K. Vora

**Affiliations:** a Department of Pharmaceutics, Manipal College of Pharmaceutical Sciences, Manipal Academy of Higher Education Manipal India deepanjan.datta@manipal.edu deepanjandtt@gmail.com; b Department of Pharmacognosy, Manipal College of Pharmaceutical Sciences, Manipal Academy of Higher Education Manipal India; c Fluoro-Agrochemicals, CSIR-Indian Institute of Chemical Technology Tarnaka Hyderabad 500007 India; d Faculty of Pharmacy, Chiang Mai University Chiang Mai 50200 Thailand; e Office of Research Administration, Chiang Mai University Chiang Mai 50200 Thailand; f School of Pharmacy, Queen's University Belfast 97 Lisburn Road Belfast BT9 7BL UK

## Abstract

The landscape of drug delivery has undergone a transformative shift with the emergence of adaptive therapeutics, smart microdevices engineered to respond dynamically to specific physiological or externally applied stimuli. These stimuli-responsive systems represent a significant advancement over conventional delivery methods by offering precise spatiotemporal control over drug release, thereby enhancing therapeutic efficacy and minimizing off-target effects and systemic toxicity. This review delves into the foundational design principles and material innovations that underpin these responsive microdevices. It highlights the role of endogenous stimuli such as pH gradients, temperature fluctuations, enzymatic activity, and redox, ionic, and hypoxia-responsive elements in activating drug release mechanisms. The integration of cutting-edge microfabrication techniques, nanomaterials, and bioinspired architectures has enabled the development of devices that are not only highly sensitive and selective but also capable of navigating complex biological environments. Furthermore, the article explores and examines the challenges associated with scalability, long-term biocompatibility, biosafety, and toxicity of implanted microdevices. Emerging trends such as AI-enhanced feedback loops, wearable biosensors, and closed-loop delivery platforms are discussed as future directions that could redefine personalized medicine. By bridging engineering ingenuity with biomedical imperatives, stimuli-responsive microdevices are poised to revolutionize drug delivery, offering intelligent, patient-centric solutions that adapt in real time to the dynamic needs of the human body. Notably, stimulus-responsive microdevices may soon facilitate localized delivery of imaging contrast agents, pharmaceuticals, genes, and mRNA; enable minimally invasive surgical procedures; and assist in cellular micromanipulation.

## Abstract 2

The drug delivery landscape has advanced with the emergence of adaptive therapeutics, comprising smart microdevices engineered for controlled, endogenous stimulus‑responsive drug delivery.

## Graphical abstract

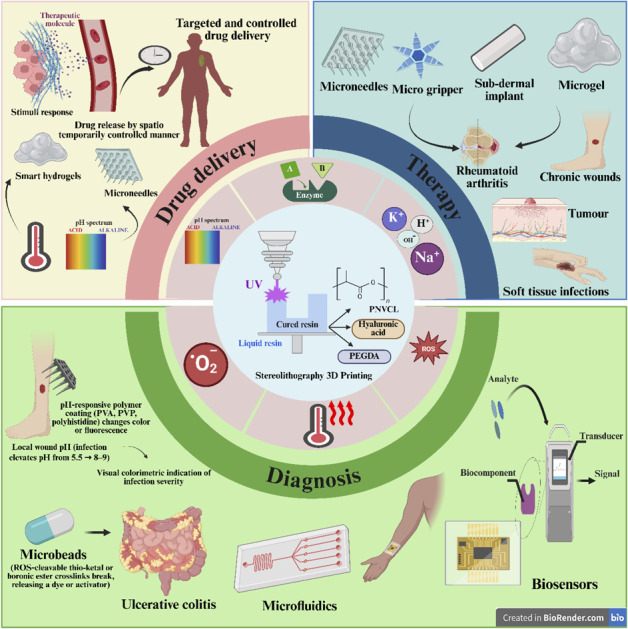

## 1. Introduction

The paradigm of drug delivery is undergoing a transformative shift from passive administration toward adaptive therapeutics, systems capable of sensing, responding, and dynamically adjusting to physiological cues. Recent advancements in this field have shown that patternable stimuli-responsive materials at the micro- or submicrometric scale exhibit significant potential, as they may be utilized in applications such as drug-release systems.^1^ Molecular architectures capable of undergoing externally triggered, reversible conformational changes, such as contraction or expansion, are poised to play a pivotal role in the development of next-generation all-organic functional devices. These systems hold promise for a diverse range of applications, including artificial muscles, site-specific drug delivery, responsive sensing platforms, soft robotics, and molecular-scale electronics. To achieve a coherent and measurable macroscopic response, these materials are often meticulously designed so that the conformational transitions of individual molecular subunits are synergistically amplified. This collective behaviour is typically modulated by external stimuli such as light, thermal energy, pH variations, metal ion coordination, solvent polarity, or applied electric fields.^2^

A range of stimuli-responsive materials has been developed to enable efficient and localized delivery of therapeutics, thereby mitigating side effects. Certain materials react to internal stimuli, autonomously controlling and selectively discharging their payloads when activated by a mechanism pertinent to a pathogenic occurrence. Alternative materials are externally actuated, providing enhanced spatial and temporal control, with low drug release in the ‘off’ state. Materials receptive to external stimuli are deemed more clinically relevant than those stimulated passively or internally. The alteration in the behaviour of stimuli-responsive materials within complex biological systems often arises from reactions to a confluence of external stimuli. It is plausible to assert that the most significant progress in the therapeutic selectivity of stimuli-responsive materials will result from employing synergistic responses to a combination of environmental stimuli.^3–5^ The drug delivery system that relies on stimuli encompasses the occurrence that affects an action at a specific location or target tissue, resulting in beneficial effects for drug release through diverse mechanisms. Stimuli-responsive materials experience a physical or chemical transformation when exposed to an external trigger. These materials demonstrate a remarkable ability to adapt to their surroundings and react to external stimuli by mimicking biological processes. The utilization of stimulus-triggered drug delivery systems holds significant significance in the realm of nanomedicines and nanotechnology, as it enables precise and controlled release of medication at the desired site.^6^ Moreover, recently, these stimuli-responsive materials have garnered heightened interest in biological applications owing to their capacity for spatially and temporally regulated release of theranostic compounds in response to certain stimuli.^7^ Stimuli-responsive materials have emerged as versatile platforms for the integration of multiple micro- and nanoscale devices within advanced drug delivery systems.^8^ By harnessing their ability to undergo precise physicochemical transformations in response to external cues, these materials enable spatiotemporal control over therapeutic release. This dynamic responsiveness facilitates the development of multifunctional delivery vehicles capable of navigating complex biological environments, enhancing targeting specificity, minimizing off-target effects, and improving overall therapeutic efficacy.

Microdevices have proven to be highly effective in medical applications, as they can be specifically designed to perform precise tasks with exceptional accuracy. Moreover, microdevices offer enhanced scalability and simplified fabrication processes when compared to nanocarriers. Microdevices have been utilized in several studies to administer drugs in a stimulus-responsive manner, demonstrating their applications in microfluidics, biosensors, drug delivery, tissue engineering, and diagnostics, among others.^9–13^ Stimuli-responsive microdevices offer a promising solution by responding to specific triggers, such as pH, temperature, or biochemical signals, thereby enabling the controlled and targeted release of therapeutics. Localized or systemic administration using microdevice technologies circumvents limitations, including serum proteases, serum protein adsorption, renal clearance, and infusion or hypersensitivity reactions.^14^ Microdevices encompass injectable hydrogels, coatings for implantable devices, and transdermal patches. Depot systems may have reservoirs of active ingredients encased within a physical barrier or agents distributed across a stable matrix material.^15,16^

Stimuli-responsive microdevices are sophisticated platforms engineered to undergo controlled alterations in their physicochemical properties upon exposure to specific environmental cues, thereby facilitating targeted and regulated drug release. These stimuli are broadly classified into endogenous triggers, which originate within the biological milieu, and exogenous triggers, which are applied externally. Central to the advancement of adaptive therapeutics is the strategic exploitation of endogenous signals, such as pH gradients, temperature fluctuations, redox conditions, enzymatic activity, and hypoxic environments, which are often characteristic of pathological sites. For instance, pH-sensitive hydrogels can swell or degrade in acidic tumor niches,^17^ while enzyme-cleavable linkers allow selective activation in inflamed or infected tissues.^18^ Hypoxia-responsive microneedles further exemplify how microdevices can be tailored to exploit pathological hallmarks for targeted intervention.^19,20^ Importantly, these systems are not merely passive carriers but adaptive actuators, capable of modulating drug kinetics in response to fluctuating physiological conditions. By responding to these intrinsic cues, microdevices enable precise spatiotemporal modulation of drug delivery, enhancing therapeutic efficacy while minimizing systemic side effects. Notably, stimuli-responsive microdevices can be engineered to react to exogenous cues, such as light, magnetic fields, ultrasound, and electric signals, among others, for precise, on-demand drug release. Light-responsive compounds (*e.g.*, azobenzene, spiropyran) undergo structural changes upon irradiation, while magnetic-field responsive systems enable remote actuation, hyperthermia, or targeted navigation.^21,22^ However, microdevices that incorporate exogenous materials, especially nanoparticles, polymers, or metallic components, face several critical limitations that exact a substantial tissue toll. First, the physicochemical characteristics of these materials, such as size, surface charge, and composition, can provoke oxidative stress, inflammation, and even genotoxic effects in surrounding tissue, triggering apoptosis or necrosis through reactive oxygen species (ROS) generation and DNA damage. Second, degradation products from polymeric or metallic payloads may leach toxic ions or acidic byproducts (*e.g.*, lactic or glycolic acid), which exacerbate local cytotoxicity and inflammation, impair tissue regeneration, and potentially disturb systemic physiology. Third, unintended accumulation and persistent retention of microdevice components in organs, particularly the liver, spleen, and kidney, can lead to long-term organ toxicity, immunogenic responses, and fibrosis. Fourth, immune activation through foreign-body reactions often results in granuloma formation and impaired integration of the device with native tissue, compromising efficacy and safety. Finally, these biosafety risks are compounded by challenges in precisely controlling degradation kinetics, dose distribution, and long-term biocompatibility, all of which demand extensive preclinical evaluation and complicated regulatory oversight before clinical translation. Because of the significant challenges posed by exogenous materials, including unpredictable degradation, immunogenicity, local and systemic toxicity, and complex regulatory hurdles, this review pivots toward endogenous stimuli-responsive strategies as a safer and more physiologically attuned alternative. By leveraging the body's inherent biochemical cues, these systems aim to minimize adverse reactions, enhance biocompatibility, and achieve precise drug release, thereby addressing the limitations that have long hindered the clinical translation of exogenous-material-based microdevices.

## 2. Literature search strategy

This review highlights recent progress in endogenous stimuli-responsive microdevices engineered for precision and safety in biomedical drug delivery. The literature search was designed to capture advances in device design, mechanistic understanding, and therapeutic implementation across micro- and nanoscale platforms.

A systematic search was carried out across PubMed, Web of Science, Scopus, SciFinder, ScienceDirect, and Google Scholar. A structured keyword strategy was adopted, incorporating three thematic domains: device platforms: “smart microdevices”, “micro/nanodevices”, “hydrogel microdevices”, “microneedles”; endogenous stimuli: “pH-responsive systems”, “ROS-responsive therapeutics”, “enzyme-responsive delivery”, “hypoxia-responsive materials”; “application context: “stimuli-responsive drug delivery”, “endogenous stimuli AND drug delivery microdevices”.

Boolean operators were applied to refine the search and ensure comprehensive coverage of mechanistic and application-focused studies. Only peer-reviewed, English-language, and full-text articles were considered. The search window spanned January 2005 to December 2026, enabling the inclusion of both foundational studies and contemporary developments in endogenous-stimulus-responsive drug delivery technologies.

### 2.1. Inclusion and exclusion criteria

To maintain an analytical rigour, pre-defined criteria were applied during screening.

#### 2.1.1 Inclusion criteria

Studies were retained if they described the design principles, mechanisms, or functional attributes of endogenous-stimulus-responsive micro or nanodevices; evaluated the therapeutic performance, biocompatibility, or biomedical relevance of such systems; and provided mechanistic, conceptual, or technological insights through primary research or comprehensive reviews relevant to stimulus-guided drug delivery.

#### 2.1.2 Exclusion criteria

Articles were excluded if they focused on non-biomedical or industrial devices; involved non-stimulus-responsive systems that lacked endogenous trigger relevance; were conference abstracts, non-peer-reviewed sources, or publications without adequate methodological detail; and lacked experimental, mechanistic, or conceptual relevance to endogenous stimuli-mediated drug delivery.

## 3. Stimuli-responsive materials: changing the game in microdevice mastery

Stimuli-responsive materials are dramatically transforming the capabilities of modern microdevices by introducing dynamic functionalities and adaptive behaviour, particularly within the field of biomedical engineering. These advanced matrices can alter their chemical and physical properties in response to specific external stimuli such as temperature, pH, light, electric or magnetic fields, and even biological signals.^23,24^ By leveraging such responsiveness, engineers can equip microdevices with the ability to sense their environment, actuate precisely, and deliver therapeutic or diagnostic interventions with unmatched specificity. The integration of stimuli-responsive matrices into microdevices has led to significant advances in biomedical engineering, including wearable sensors,^25,26^ implants,^27,28^ microneedles,^29,30^ actuators,^31,32^ and soft robotics.^33^ For instance, temperature-responsive hydrogels facilitate on-demand drug release in cancer,^34^ wound healing,^35^ arthritis,^36^ ocular diseases,^37^ and neurological disorders.^38^ In contrast, pH- and enzyme-specific materials enable targeted therapy with reduced systemic side effects in gastrointestinal diseases,^39^ infections,^40^ inflammatory conditions,^41^ and chronic wounds.^42^ A key advantage of these matrices is their customizable architecture, which enables precise tuning of their sensitivity, mechanical properties, and biofunctionality to meet the diverse needs of microdevice applications. As research continues to unravel new stimuli and composite designs, stimuli-responsive materials and polymers are poised to expand the toolbox for next-generation microdevice mastery, enabling more autonomous, efficient, and patient-centered technological solutions. Notably, a summary of stimuli-responsive microdevices for drug delivery is presented in Table 1, detailing the various types of microdevices, stimulus modalities, cargo, polymer composition, response behaviour, drug loading, biocompatibility, and therapeutic applications.

**Table 1:** Comparative analysis of endogenous stimuli-responsive materials in microdevice-based drug delivery systems^a^

Different types of endogenous stimuli	Type of microdevice	Cargo	Stimuli-responsive material	Response/response time	Drug loading capacity	Biocompatibility	Therapeutic outcome	Ref.
Temperature-responsive	Soft microrobot	DOX	Alginate/NIPAM	Thermoresponsive swelling/deswelling; rapid response to NIR-induced temperature change	80.8%	Yes	Controlled release *via* NIR-triggered actuation; potential for targeted therapy	43
				Response time: 6 h				
	Microfluidic hydrogel-based microcarriers	DOX	Poly(*N*-vinylcaprolactam)	Reversible swelling/deswelling near LCST	34%	Yes	Controlled release triggered by mild hyperthermia; potential for localized therapy	44
	3D-printed PNAGA hydrogel microrobot	DOX	PNAGA	Shrinking and drug release above LCST; seconds to minutes	—	Yes	Targeted cancer therapy	45
pH-responsive	Marsupial robotic system constructed by integrating chemical/magnetic hybrid nanorobots (child robots) with a miniature magnetic continuum robot (mother robot)	DMSNs	Fe_3_O_4_ and urease	Magnetic navigation into the brain cavity; pH-triggered drug release in acidic tumor microenvironment; response within minutes	75%	Yes	Enables precise cross-scale targeting and localized drug release in glioblastoma; bypasses the blood–brain barrier	46
	Double-layered MOF-based microswimmer	DHA, 5-FU, CPT-11, and DOX	ZIF-8 and MIL-100	pH-triggered release in acidic tumor microenvironment; magnetic navigation	DHA/5-FU = 52.6% DHA/CPT-11 = 46.4% DHA/DOX = 96%	—	Synergistic dual-drug delivery combats resistance; targeted tumor therapy	47
	Asymmetric microfluidic chitosan device	Minocycline	DMAEMA	Swelling and drug release in acidic pH over 12 h	—	Yes	Targeted infection control in bone defects	48
Enzyme-responsive	Microneedles	aPD1, 1-methyl-dl-tryptophan (1-MT)	HA	Immune activation within minutes	18%	Yes	Used in melanoma therapy	49
	Hydrogel actuator	Trypsin	PEGDA	Trypsin-triggered expansion	—	—	Drug delivery, adaptive wound dressings, and tissue engineering scaffolds	50
	ChemoBots	DOX	PEGDA	Controlled release was activated by matrix metalloproteinases (MMP2 and MMP9)	23.5%	Yes	Used for triple-negative breast cancer	51
Redox-responsive	Wireless bioelectronic implantable device	DOX	Au microelectrodes in PDMS matrix	Programmable release *via* voltage pulses; responsive within seconds to minutes	78.42%	Yes	Used in breast cancer	52
	Microneedles	Zinc phthalocyanine (ZnPc), doxorubicin hydrochloride (Dox)	HA	Acid-triggered degradation + O_2_ evolution + ROS generation within minutes	—	Yes	Targeted tumor therapy	53
	4D-printed needle panel meter	Glucose oxidase, lactate oxidase	EDDT, PEGDA	Geometric deformation	—	—	Non-invasive glucose (GLU) and lactate (LAC) detection from blood, sweat and urine samples	54
Hypoxia-responsive	Hypoxic gradient within a micropatterned monolayer culture of human cancer cells integrated with an oxygen sensor	Tirapazamine, pimonidazole	PDMS	Oxygen gradient formation	—	Yes	Used in targeted drug delivery	55
	Microfluidic device with ratiometric oxygen sensors	Doxorubicin and tirapazamine	PDMS	Real-time oxygen monitoring	—	Yes	Used in parallel analysis of tumor spheroid response to hypoxia, supports drug screening and mechanistic studies	56
	Microvascular chip	Sickle RBCs	PDMS	Rapid oxygen modulation	—	Yes	Used in the mechanistic study of vaso-occlusion, oxidative stress, and endothelial dysfunction in sickle cell disorders	57
Ionic-responsive	Microgel	Procaine hydrochloride	Carbohydrazide FGM and OA	Shape/stiffness change upon ion exposure	—	Yes	3D/4D bioprinting, tissue engineering, cancer modeling	58
	Stretchable organo-hydrogel-based microdevice	Gelatin and ferric-ion-cross-linked polyacrylic acid	Carbon, acetylene black, and poly(vinylidene fluoride)	Self-healing within minutes	—	Yes	Wearable biosensors, implantable diagnostics, soft robotics	59
	Microneedles	Mesenchymal stem cell (MSC)-exosomes	PVA	Sulfate ions enhanced tip stiffness for effective skin penetration, while nitrate ions softened the tips post-insertion, promoting tissue adaptation, and sustained exosome release	—	Yes	Used in tissue regeneration and diabetic wound healing	60

a DOX – doxorubicin; DMSNs – dendritic mesoporous silica nanoparticles; DHA – dihydroartemisinin; 5-FU – fluorouracil; CPT-11 – irinotecan hydrochloride; NIPAM – *N*-isopropylacrylamide; PVA – polyvinyl alcohol; PNAGA – poly-*N*-acryloyl glycinamide; DMAEMA – 2-(dimethylamino) ethyl methacrylate; HA – hyaluronic acid; PEGDA – polyethylene glycol diacrylate; EDDT – 2,2′-(ethylenedioxy) diethanethiol; PDMS – polydimethylsiloxane; FGM – functionalized gelatin methacrylate.

Stimuli-responsive materials are engineered to undergo controlled changes in their structure, solubility, and mechanical properties in response to specific environmental triggers such as temperature, pH, enzymes, light, magnetic fields, biological or electrical signals. These materials play a pivotal role in biomedical applications, particularly in microdevice-based drug delivery systems, where precision and adaptability are essential.^61,62^ Inorganic materials, such as Fe_3_O_4_ nanoparticles, respond to magnetic fields for the remote-controlled release of doxorubicin in the treatment of breast cancer. Meanwhile, TiO_2_ and azobenzene derivatives enable light-triggered activation, enhancing therapeutic efficacy, particularly in tumor targeting and controllable drug release. Biohybrid platforms incorporating *Spirulina platensis* exhibit antioxidant and bioadhesive properties, which facilitate wound repair during full-thickness skin injuries. Additionally, biotin–avidin systems enable ligand-specific cellular drug targeting.^63^ The PPy/Fe_3_O_4_/Pt composite comprising polypyrrole (PPy), magnetite nanoparticles (Fe_3_O_4_), and platinum (Pt) represents a multifunctional material system designed for the electrochemical removal of estrogenic contaminants from water. This hybrid structure integrates the charge-tunable affinity of PPy, the magnetic responsiveness of Fe_3_O_4_, and the catalytic conductivity of Pt to regulate the aggregation and capture of estrogenic compounds such as estradiol and ethinylestradiol. Biological stimuli-responsive materials are engineered to interact with specific biomolecules or cellular components, enabling highly selective and context-aware responses in diagnostic and therapeutic microdevices. For instance, systems incorporating *E. coli* bacteria as a biological recognition element utilize surface-functionalized polymers or aptamers that exhibit specific binding to bacterial markers associated with tumor-promoting infections, facilitating early cancer diagnosis through microbial profiling.^64^ Similarly, DNA-controlled systems utilize conformational changes, such as strand displacement, hairpin opening, or G-quadruplex formation, in response to oncogenic sequences or biochemical cues. These transitions trigger measurable outputs, such as fluorescence or electrochemical signals, enabling the sensitive detection of cancer biomarkers at early stages.^65^ Collectively, these materials enhance the functionality of microneedles, microfluidic chips, and implantable devices, offering on-demand, patient-tailored therapeutic solutions with reduced systemic toxicity.

Stimuli-responsive polymers belong to the versatile class of smart materials that undergo reversible changes in their physical or chemical properties in response to specific environmental triggers such as temperature, pH, electric fields, light, magnetic fields, redox conditions, solvents, or biological molecules. These polymers are widely used in drug delivery systems, biosensors, and microdevices due to their ability to provide controlled, site-specific, and on-demand therapeutic action.^66^ For example, temperature-responsive polymers such as poly(*N*-isopropylacrylamide) (PNIPAM), poly(*N*-vinylcaprolactam) (PVCL), and Pluronic block copolymers exhibit sol–gel transitions near body temperature, making them ideal for injectable hydrogels and localized drug delivery.^67,68^ pH-responsive polymers such as poly(acrylic acid), chitosan, and poly(l-histidine) contain ionizable groups that respond to acidic or basic environments, enabling targeted delivery in tumors or inflamed tissues.^69,70^ The pH-triggered oral drug delivery system was developed by capping mesoporous silica SBA-15 with pH-responsive polymer PAA *via* a facile graft-onto strategy, and it was used to deliver anti-cancer drug doxorubicin for the treatment of colon cancer.^71^ The pH-responsive biodegradable hydrogels made from four types of pH-sensitive PAA derivatives and poly(l-glutamic acid) as a cross-linker were applied for oral delivery of insulin.^72^ pH-responsive nanoparticles made of Eudragit RL 100 and Eudragit RL 100-poly(lactic-*co*-glycolic acid) were used as drug delivery systems for diclofenac sodium in an intestinal pH condition of 6.8.^73^

The microspheres made of pH-responsive Eudragit RS100 were used as a drug delivery system for the acidic drug ibuprofen.^74^ Eudragit S100, as a pH-responsive polymer, was coated onto the liposomes by a fast and organic solvent-free method for the colonic delivery of curcumin.^75^ The transparent film formed from Eudragit E100 polymer was used for transdermal therapy since it showed good adhesion to the skin. The loaded drug nicorandil was released due to erosion of the hydrophilic Eudragit E100 polymer, and the release of the drug was observed to be 100% within 20 min.^76^ Electric field-responsive polymers, such as polyaniline (PANI) and polypyrrole (PPy), alter their shape or conductivity in response to electrical stimulation, making them suitable for use in MEMS and electrochemical devices.^77^ Light-responsive polymers, including azobenzene- and spiropyran-containing systems, react to UV or NIR light for spatially controlled activation.^78^ Magnetic-responsive polymers, often composites with Fe_3_O_4_ nanoparticles, allow remote-controlled actuation or heating.^79^ Additionally, polymers responsive to solvent polarity or ionic strength, such as polyvinyl alcohol (PVA), polyacrylamide (PAAm), enable highly selective and adaptive therapeutic platforms for drug delivery.^80^ Collectively, these polymers offer modularity, tunability, and responsiveness, which are essential for advancing personalized medicine and next-generation biomedical devices.

## 4. Design considerations and comparative analysis of microdevices *vs.* nanocarriers

**Fig. 1:**
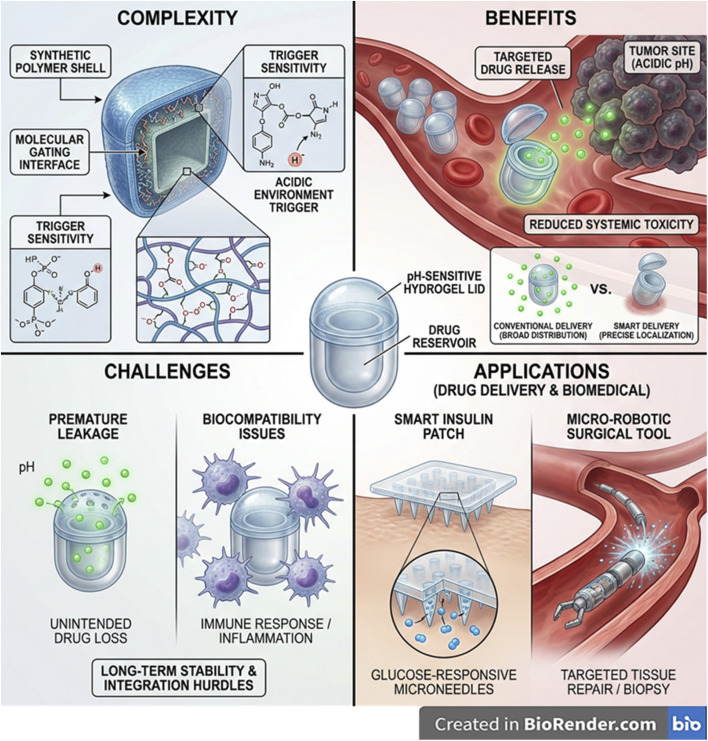
Schematic overview illustrating the complexity, benefits, challenges, and biomedical applications of endogenous stimuli-responsive microdevices. (Top left) Complexity arises from multilayered architectures with synthetic polymer shells, molecular-gating interfaces, and pH-responsive chemistries activated in acidic microenvironments. (Top right) Benefits include targeted drug release in diseased tissues such as acidic tumor sites, improving localization and reducing systemic toxicity compared with conventional delivery. (Bottom left) Key challenges include premature pH-triggered leakage, biocompatibility-related immune responses, and long-term stability limitations. (Bottom right) Applications include smart insulin patches with glucose-responsive microneedles, micro-robotic tools for minimally invasive repair or biopsy, and implantable pH-responsive drug reservoirs. The figure highlights how endogenous cues enable controlled, site-specific therapy while emphasizing remaining translational barriers. This image has been created as a Creative Common using the BioRender software (https://www.biorender.com/).

Designing multiple endogenous stimuli-responsive microdevices requires integration of materials, architecture, and microfabrication approaches that enable multi-cue sensing and controlled actuation at the microscale. Key considerations include device geometry (reservoirs, channels, compartments), mechanical robustness, biocompatibility, and the ability to incorporate diverse triggers such as pH, enzymes, redox state, or ionic changes without compromising structural stability (Fig. 1).^81^ These stimuli-responsive microdevices face persistent challenges largely tied to their size, multi-layered architecture, and long-term residence within tissues. First, achieving precise biomarker sensing remains difficult because endogenous cues such as pH, cytokines, or enzymes are often spatially heterogeneous and dynamic; microdevices respond only to the microenvironment immediately surrounding their surfaces, leading to inaccurate or delayed activation when biomarkers fluctuate or diffuse slowly.^82–84^ Biofouling and fibrotic encapsulation severely impair responsiveness over time, as proteins, cells, and ECM components clog or insulate responsive membranes, reducing permeability and altering trigger thresholds.^85,86^ Mechanical durability and long-term biostability are unresolved issues because cyclic swelling, degradation, and micro-valve fatigue compromise reliability, especially for chronic implants.^87^ Integration of orthogonal stimulus-responsive chemistries into micro-scaled structures remains technically challenging, and maintaining consistent, GMP-grade microfabrication with tight tolerances is still difficult. Additionally, sterilization sensitivity (heat, radiation, chemicals) can degrade responsive linkers or device architecture.^88^ Finally, regulatory pathways are unclear for multi-trigger implantable systems, especially those combining biochemical sensing with structural components, translating to slow and expensive.^89,90^

**Fig. 2:**
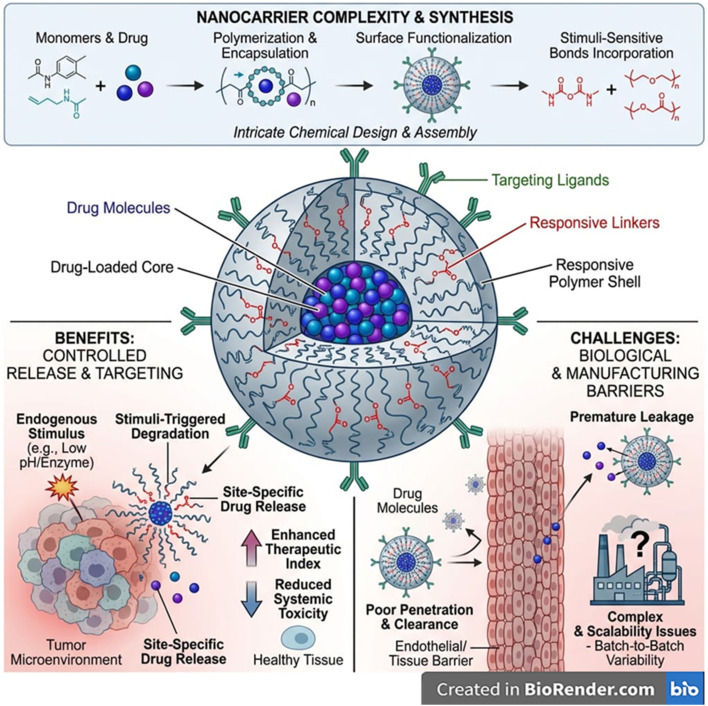
Schematic illustration of the complexity, benefits, challenges, and biomedical implications of endogenous stimuli-responsive nanocarriers. (Top panel) Multistep synthesis—spanning monomer/drug selection, polymerization, encapsulation, surface functionalization, and incorporation of stimuli-sensitive bonds—highlights the chemical complexity of responsive nanocarriers. (Central panel) The nanocarrier structure features a drug-loaded core, responsive polymer shell, targeting ligands, and stimuli-cleavable linkers enabling controlled, site-specific activation. (Bottom left) Benefits include endogenously-triggered degradation, localized release within diseased microenvironments such as tumors, enhanced therapeutic index, and reduced systemic toxicity. (Bottom right) challenges include premature leakage, limited tissue penetration, rapid clearance, and manufacturing barriers such as scalability and batch variability. The figure underscores both the promise and translational hurdles of multi-stimuli-responsive nanocarrier systems. This image has been created as a Creative Common using the BioRender software (https://www.biorender.com/).

In contrast, nanocarrier design focuses heavily on surface chemistry, colloidal stability, and responsiveness within tight nanoscale constraints. Architects must choose chemistries that remain inert during circulation yet respond sharply to specific endogenous cues, while maintaining uniform size, charge, and dispersity (Fig. 2).^91^ Nanocarriers must resist protein corona formation, avoid premature aggregation or opsonization, and exploit intracellular triggers such as endosomal pH or cytosolic GSH.^92,93^ Because nanocarriers enter cells and traverse biological barriers, their design prioritizes stealth behaviour, efficient loading of small or macromolecular drugs, membrane-interacting features for uptake, and orthogonality of stimuli-responsive linkages within a confined nanoscale architecture.^94^ For nanocarriers, the biggest unresolved challenges arise from their interaction with complex biological fluids and their dependence on precise molecular-scale triggers. Endogenous stimuli such as pH, ROS, GSH, or enzymes often exhibit shallow gradients, making it difficult for nanosystems to distinguish pathological from healthy tissue with true specificity.^95,96^ The protein corona remains a major unresolved barrier; adsorbed proteins mask targeting ligands and responsive groups, alter surface charge, change circulation time, and can entirely block stimulus-responsive mechanisms.^97^ Nanocarriers also struggle with colloidal instability, premature drug leakage, and inconsistent biodegradation rates, especially when multiple-responsive linkers interact or degrade unpredictably.^98–100^ Achieving reproducible multi-stimuli-responsiveness is difficult because the nanoscale limits the integration of orthogonal chemical motifs without cross-reactivity. *In vivo*, rapid clearance by the liver, spleen, and mononuclear phagocyte system reduces the probability of reaching the intended target in sufficient quantity to activate meaningfully.^101^ Moreover, inter-patient variability in ROS, enzyme levels, or metabolic states makes dose–response relationships less predictable.^102^ Lastly, analytical characterization at the nanoscale is extremely limited; regulators lack standardised metrics for activation thresholds, corona effects, or multi-trigger kinetics, leaving the clinical translation landscape uncertain.^103^

Endogenous single- and multiple-stimuli-responsive microdevices and nanocarriers represent two converging but functionally distinct strategies for precision drug delivery and biomedical sensing (Table 2). In broader biomedical use, nanocarriers integrate into scaffolds or wound dressings to provide microenvironment-guided release,^104^ whereas microdevices support closed-loop systems, targeted electromechanical actuation, or multi-compartment combination therapy.^105,106^ Overall, nanocarriers offer superior diffusivity and intracellular precision, while microdevices deliver higher control, longevity, and payload versatility, together forming complementary platforms in advanced responsive therapeutics. Together, these technologies provide complementary capabilities: microdevices excel in controlled, site-specific, long-duration therapy, whereas nanocarriers provide systemic reach and intracellular precision. Advancing clinical translation will require addressing material biostability, trigger orthogonality, manufacturing reproducibility, and standardized evaluation of multi-stimulus responses across both platforms.

**Table 2:** Comparative overview of endogenous single and multi-stimuli responsive microdevices *vs.* nanocarriers

Endogenous stimuli	Complexity	Benefits	Challenges in applications	Ref.
**Endogenous single-stimuli responsive microdevices**				
Temperature	Requires precise tuning of thermo-responsive polymers, regulatory emphasis on demonstrating safe, predictable temperature-triggered release, and real-world translation is limited by variable tissue temperatures	Precise activation at locally inflamed tissue, stable spatial confinement ensures controlled release	Limited temperature differential during *in vivo* studies, risk of premature activation	107–110
pH	Needs strict control of pH-responsive polymers for consistent activation, must prove selective response in acidic pathological target areas, and translational reliability is constrained by fluctuation in wound pH	Highly selective and predictable activation in acidic infection/wound sites, microdevice geometry minimizes off-target effects	pH varies across the tissues, which may cause inconsistent triggering	111–118
Enzyme	Uses enzyme-cleavable polymers requiring specialized synthesis, regulatory authorities expect strong evidence of enzyme-specific activation and safe degradation products. Clinical translation is challenged by patient-to-patient enzyme variability	Device-scale enzyme-cleavable architecture allows ultra-specific activation and deeper control over release kinetics	Inter-patient enzyme variability, slower kinetics	119–121
Hypoxia	Incorporates oxygen-sensitive moieties with demanding stabilization requirements, requires regulatory scrutiny on selective activation under hypoxic gradients, and translational performance is hindered by spatially unstable oxygen levels	Stable platforms allow incorporation of oxygen-sensitive moieties with precise spatial targeting, excellent for chronic wounds	Unstable hypoxia gradients, unstable, more complex manufacturing process	122–125
Redox	Requires meticulously balanced ROS-sensitive polymers to avoid premature activation, regulatory validation needed for selective response at pathological ROS levels, and translational consistency is affected by dynamic oxidative stress	Strong, localized redox-triggered release due to high ROS levels at infected sites, reduced risk of systemic activation	Levels of ROS fluctuate over-activation risk	126 and 127
Ionic	Simple to fabricate but difficult to tune for selective ion-dependent activation, regulatory authorities require proof of stable performance across mixed ionic environments, translation is limited by variability in wound exudate and electrolytes	Rapid swelling/dissolution with excellent localization, the microdevice structure restricts undesired ion-driven diffusion	Difficult to achieve high selectivity due to the broad distribution of ions	128

**Endogenous single-stimuli responsive nanocarriers**				
Temperature	Requires precise control of thermo-responsive polymers; small temperature shifts during synthesis/storage alter carrier stability during manufacturing, requires predictable temperature-triggered activation without unintended release at normal body temperatures, and highly variable tissue temperatures can reduce reliability and risk premature activation with respect to a translational point of view	Controlled drug release at fever/inflammation sites	Easily destabilised by temperature changes	129–132
pH	Needs stringent control of acid-labile polymers/coatings to maintain consistent activation thresholds across batches, selective activation in target acidic environments while avoiding release in normal physiological pH, wide pH variability across wounds/target size limits consistent targeting and performance	Ideal for infection-associated acidic sites, controlled drug release	High risk of off-target activation in acidic organs (stomach, endosomes), systemic dispersion reduces specificity	133 and 134
Enzyme	Relies on specialized synthesis of enzyme-cleavable polymers and stable incorporation into nanosystems, requires strong enzyme-specific activation data and safety of all enzymatic degradation products, enzyme levels differ significantly across target sites/tissues, making drug release less predictable	Reduced premature degradation, site-specific drug delivery	Enzyme-triggered nanocarriers often suffer from slow, incomplete activation and enzymatic degradation before reaching the target tissue	135 and 136
Hypoxia	Integration of oxygen-sensitive moieties requires careful stabilization and stringent quality control in the field of manufacturing, lacks the provision of evidence of selective activation under clinically relevant hypoxia without off-target behaviour, and highly unstable and heterogeneous oxygen gradients limit consistent -patient performance	Improved off-target irritation, enhanced penetration of the drug into stressed tissues	Hypoxia-responsive nanocarriers often poorly penetrate tissue, have inconsistent oxygen gradients, and show inefficient activation	137–139
Redox	Requires precise engineering of ROS/GSH-sensitive polymers, prone to premature activation during fabrication and storage. Activation occurs only at pathological oxidative stress levels, ensuring tissue safety. Rapid fluctuations in ROS/GSH across infection sites introduce variability in release kinetics	Cleavage of disulfide bonds in high-GSH/ROS microenvironments	High chance of premature activation inside cells or blood; poor control over redox-triggered kinetics	140 and 141
Ionic	Requires only simple fabrication but is difficult to tune for selective response within mixed ionic environments, lacks the demonstration of stable performance across variable physiological ionic strengths and prevents uncontrolled dissolution	Moderate ionic-responsive swelling/dissolution	Fast, uncontrolled response due to ubiquitous ions, difficult to maintain stability or targeted activation	142
	Diverse ionic compositions in tissues/fluids reduce targeting specificity and consistent activation			

**Endogenous multi-stimuli responsive microdevices**				
pH + temperature	Dual-responsiveness trigger polymers, calibrate p*K*_a_ and LCST, require moisture and sterility control, need to prove co-trigger fidelity, lack release at normal temperature/physiologic pH	Selectivity at acidic, inflamed sites, lowers off-target release	Narrowed physiological temperature window limits precise dual-trigger control, causing unpredictable release profiles	143–146
pH + enzyme	Requires acid-labile group/ROS-sensitive polymers, avoids cross-activation, requires the demonstration of selective co-activation and oxidative safety, pH/ROS fluctuates across tissues/body fluids	Enzyme gate prevents premature pH-only release, and deeper on-site activation	Enzyme levels vary widely across tissues and individuals, making activation inconsistent and site-specific response unreliable	147
pH + redox	Requires O_2_-sensitive moieties and ROS-labile polymers, requires stabilization during fabrication, requires validation under dynamic O_2_/ROS, has spatial heterogeneity, requires monitoring	Synergistic specificity in acidic, oxidative niches improved biofilm penetration	Redox gradients (ROS/GSH) are highly heterogeneous and often do not align with acidic regions, reducing dual-responsive precision	148
pH + hypoxia	Requires integrated acid + hypoxia modules without drift, requires stability testing, requires fidelity under variable pH/O_2_	Depth-selective activation in acidic, under-perfused tissue	Hypoxic zones are deep and poorly vascularized, limiting microdevice penetration and preventing effective dual-trigger activation	149

**Endogenous multi-stimuli responsive nanocarriers**				
pH + temperature	Requires thermo-/pH-responsive shells, narrow thresholds, colloidal stability requires co-trigger proof for no leakage at 37 °C and neutral pH, and chances for pH/*T* variability in target sites/tissues	Improved on-site release in acidic, inflamed regions reduced off-target	Precise coordination of pH-dependent swelling and thermo-responsive transitions is difficult due to the narrow safe physiological temperature range	150–152
pH + enzyme	Requires enzyme-cleavable coat + acid-labile core, batch fidelity, requires enzyme specificity + acid selectivity, patient enzyme variability	Enzyme prevents premature acid-release at the target site	Highly variable enzyme expression across tissues reduces predictable dual-trigger activation and consistent drug release	153 and 154
pH + redox	Requires protection from premature oxidation, storage control, lacks evidence of hemocompatibility, and variability in pH/ROS across tissues	High specificity to infected acidic, oxidative microenvironments	Redox gradients (ROS/GSH) often do not overlap with acidic regions, lowering the efficiency of simultaneous dual responsiveness	155 and 156
Hypoxia + redox	Requires hypoxia-sensitive pro-carriers + ROS-labile polymers, O_2_/ROS heterogeneity impacts dosing	Depth-selective activation	Hypoxic conditions and redox imbalance seldom coincide spatially, limiting synchronized activation of dual-responsive carriers	157–159
pH + hypoxia	Requires acid-triggered surface + hypoxia-responsive core	Depth-selective delivery in acidic, low-O_2_ tissues	Poor nanocarrier penetration into deep hypoxic zones prevents efficient exposure to both acidic and low-oxygen triggers	160–163
Hypoxia + enzyme	Requires dual triggers while preserving particle size/*ζ*-potential, and can cause stability issues	Sustained retention and controlled unmasking at diseased loci	Spatial mismatch between enzyme-rich regions and hypoxic microdomains leads to incomplete or delayed dual-trigger activation	164 and 165
Enzyme + ROS	Requires dual selectivity + oxidative safety dossier, pharmacodynamics vary with inflammation stage	Selective activation in protease-rich, oxidative niches tackles biofilms	ROS-rich regions may not contain sufficient enzyme concentrations, causing asynchronous activation of the enzyme- and ROS-responsive components	166 and 167
pH + temperature + enzyme	Requires multi-step functionalization, high QC burden, profiling, training and protocol complexity	Maximal pathogen-site specificity, staged biofilm disruption and drug release	Coordinating three stimuli simultaneously requires precise environmental overlap, which rarely occurs *in vivo*	168
pH + ROS + enzyme	Requires multi-step functionalization	Maximizes site-specific activation by requiring simultaneous acidic pH, elevated ROS, and high protease levels, enabling deep biofilm penetration, staged drug release, and minimal off-target exposure	Complex, heterogeneous tumor microenvironments cause inconsistent exposure to all three triggers, reducing tri-responsive efficiency	169

## 5. Blueprints in miniature: photolithography and additive manufacturing for microdevice design

Endogenous-stimuli-responsive microdevices, systems that sense and react to in-body cues such as pH, enzymes, redox state, glucose, or ROS, have become central to precision drug delivery, soft actuation, and on-chip regulation because they can couple local microenvironment changes to spatiotemporally controlled function. Recent reviews map the field's rapid growth, highlighting materials (*e.g.*, PEGDA, pNIPAM, HA, MA), response mechanisms, and biomedical use cases in drug delivery, wound healing, soft robotics, and photonics. In particular, hydrogels serve as the dominant transduction medium owing to high water content, tunable mechanics, and the ease of encoding chemical motifs that undergo swelling, cleavage, or crosslink rearrangement under endogenous triggers.^170,171^

**Fig. 3:**
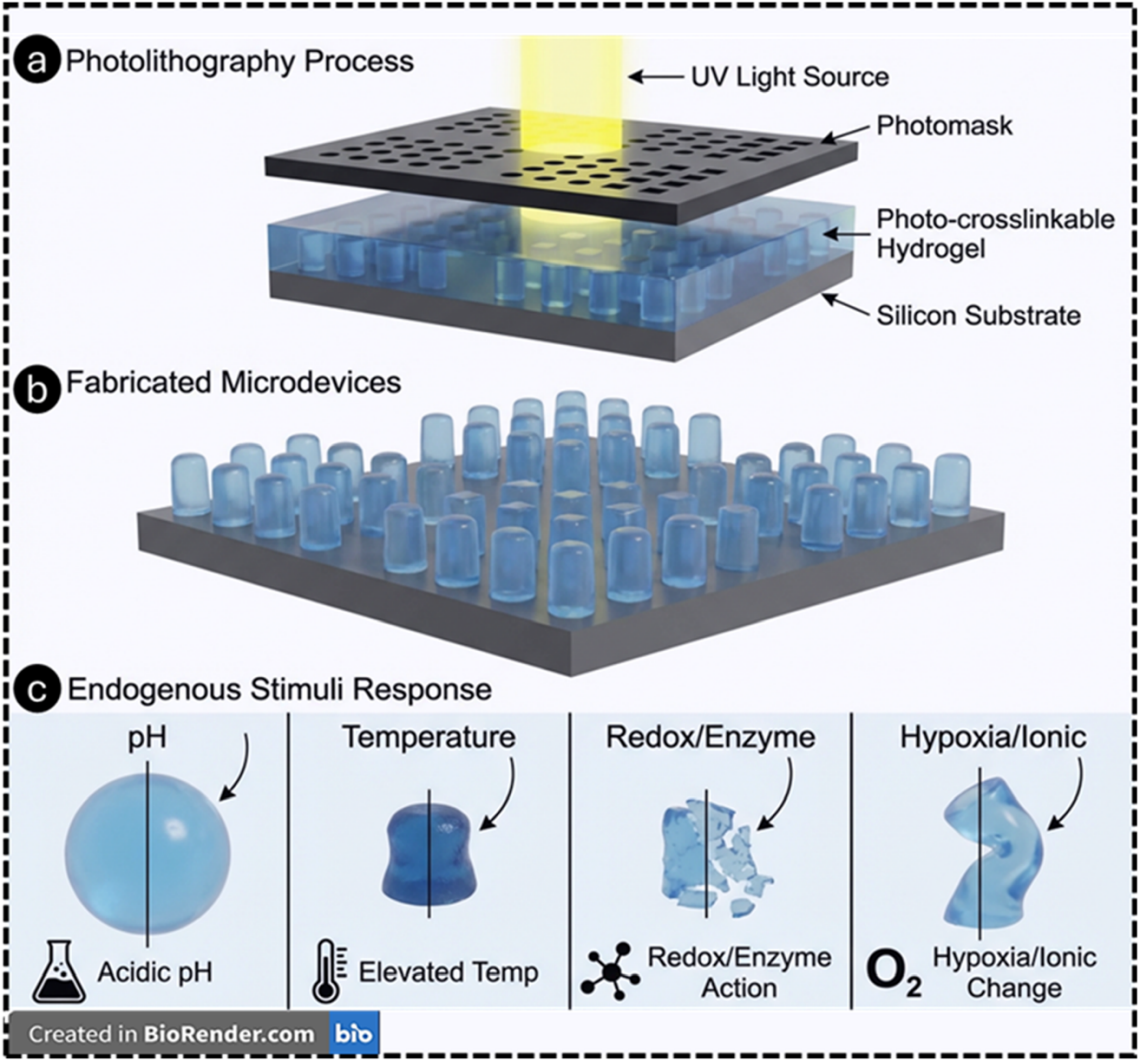
Schematic of the photolithography process, in which UV light passes through a photomask to pattern a photo-crosslinkable hydrogel layer on a silicon substrate (a). Resulting array of microfabricated hydrogel microdevices generated with high spatial precision (b). Representative endogenous stimuli—pH, temperature, redox/enzyme activity, and hypoxia/ionic changes, inducing structural or volumetric responses in the patterned hydrogel microdevices (c). This image has been created as a Creative Common using the BioRender software (https://www.biorender.com/).

Photolithography enables sub-micrometer lateral resolution, batch parallelization, and deterministic pattern transfer, capabilities that are decisive when one needs thin, planar microstructures (valves, membranes, grippers, micro-reservoirs) that respond predictably to small biochemical gradients (Fig. 3). Hydrogel photopatterning has been used to define heterogeneous crosslink density, composite layers, and anchored/hinged features, each of which governs swelling anisotropy and thus actuation or release profiles. Compared with many 3D print workflows, classical photolithography provides wafer-scale throughput and tight control of in-plane feature sizes, often tens of nanometers to a few micrometers in research settings, ideal for microfluidic channels, membranes, and valves that gate mass transfer with sensitivity to endogenous stimuli.^172,173^ Endogenous triggers (acidic pH in inflamed tissues/periodontal pockets, protease activity, elevated ROS, glucose fluctuations) are being harnessed to design “smart” dressings and carriers for oral ulcers,^174^ periodontitis,^175^ chronic wounds,^176^ and tumors. Reviews synthesize mechanisms and translational challenges, underscoring how micro-architected hydrogel features control diffusion paths and response kinetics, precisely the regime where photolithographic control of membranes and in-plane geometry is advantageous.^177^

Breger *et al.* reported self-folding microgrippers composed of photopatterned pNIPAM-*co*-AAc hydrogels (thermo/pH responsive) laminated with a stiff polymer layer have demonstrated autonomous gripping/release by swelling mismatch; iron-oxide loading adds magnetic guidance.^178^ These microgrippers were fabricated by photolithographic patterning of hydrogel bilayers, showing how 2D masks create 3D morphing devices driven by endogenous-like cues. Experiments showed that the grippers reliably transitioned around ∼36 °C, folding in opposite directions based on whether the hydrogel was swollen or collapsed, with robust performance over more than 50 cycles. Finite-element simulations validated the folding mechanism and revealed that gripper performance was highly sensitive to the modulus and thickness of both layers, identifying optimal design ranges, particularly an active hydrogel thickness around 45 µm and a stiff layer modulus near 16 MPa. Importantly, the increased stiffness achieved through the bilayer design enabled the microgrippers to effectively grasp and excise live fibroblast cells, demonstrating their potential for minimally invasive surgical tasks, tissue manipulation, and targeted therapeutic delivery. Reviews of biomimetic soft grippers further emphasize photolithography and direct printing as high-throughput routes to encode anisotropic swelling and hinge lines that convert chemical stimuli into robust shape change.^179^ Microvalves built from light-responsive or thermo-responsive hydrogel elements integrated into photolithographically defined microchannels can open/close on demand;^180^ an example uses LRH (with Fe_3_O_4_ nanoclay) microspheres that shrink within seconds of laser irradiation to open a normally closed channel and re-swell to re-close after stimulus removal.^181^ The improved uniformity of Fe_3_O_4_ nanoparticle distribution in N02 microspheres led to reproducible photothermal conversion, achieving rapid heating to ∼65 °C within 10 s of NIR irradiation that enabled stable, reversible change in the volume with <3% variation across repeated cycles. When integrated into a microfluidic chip, the LRH microsphere microvalve exhibited reliable light-triggered actuation, opening within 2 s of irradiation and reclosing in ∼18 s as the hydrogel cools and reswells, with effective operation across 1–20 mL min^−1^ flow rates and stable performance over 20 cycles per day for 5 days. Overall, the modified LRH microvalve achieves fast, repeatable, noncontact optical control of microfluidic flow, demonstrating strong potential for compact and integrated microfluidic systems. A review by Ko *et al.* on hydrogel photonic microstructures fabricated by photolithography (and related nanoimprint/e-beam methods) transduced swelling into optical shifts, yielding label-free sensing of pH, glucose, or ionic strength. This tight linkage between geometry and optical function relies on lithographic fidelity at the micro/nanoscale.^182^ Nonetheless, stimuli-responsive microneedles (MNs) have advanced substantially for endogenous-triggered delivery (*e.g.*, glucose-responsive insulin patches,^183,184^ and tumor microenvironment-responsive delivery,^185^ among others), though many MNs are fabricated *via* molding/printing. Reviews document pH/enzymatic/glucose-responsive MNs and their integration with stimuli-responsive polymers for on-demand release.^186^

**Fig. 4:**
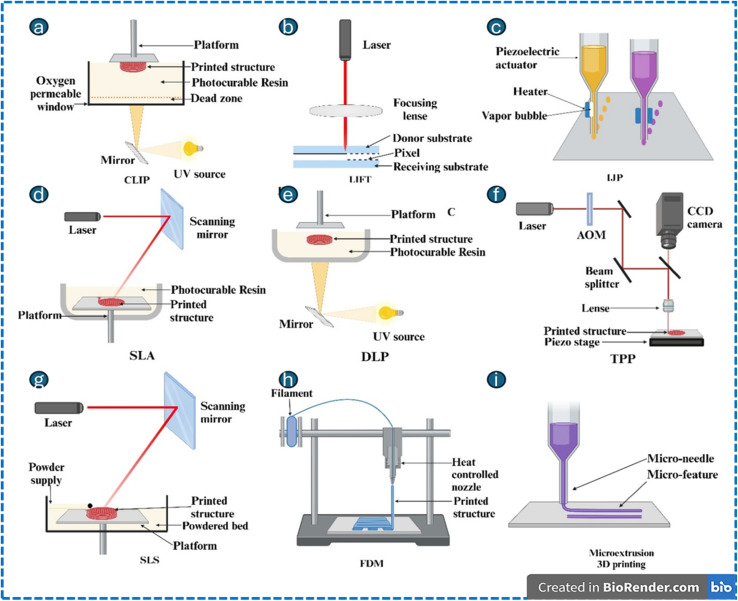
Schematic illustration of additive manufacturing techniques. Continuous liquid interface production (CLIP) (a) laser-induced forward transfer (LIFT) (b) inkjet printing (c) stereolithography (SLA) (d) digital light processing (DLP) (e) two-photon polymerization (TPP) (f) selective laser sintering (g) fused deposition modeling (FDM) (h) and microextrusion 3D printing (i). This image has been created as a Creative Common using the BioRender software (https://www.biorender.com/).

In contrast, additive manufacturing (AM) is a layer-by-layer fabrication method that enables the creation of complex 3D geometries and multi-material structures with reduced processing time. AM techniques such as stereolithography (SLA), digital light processing (DLP), two-photon polymerization (TPP), selective laser sintering (SLS), fused deposition modeling (FDM), continuous liquid interface production (CLIP), laser-induced forward transfer (LIFT), inkjet printing, and microextrusion printing are being utilized to fabricate microbots, providing varying resolution/compatibility to materials to meet a broad range of applications (Fig. 4).^83^ The stimuli-responsive micro/nanorobots involve material that responds to thermal, photic, pH, ultrasonic, magnetic, biological, or ionic triggers, enabling applications in precision medicine and smart materials.^187^ Examples include microfluidics, photothermal microneedles, electroactive actuators, and scaffolds. The multi-stimuli-responsive systems provide multifunctionality in complex environments, thereby paving the way towards applications in biomedicine and industries. The various types employed in the fabrication of microdevices are comprehensively detailed in the SI file provided (Section S4.1–S4.9). The fundamental differences between photolithography and additive manufacturing are mentioned in Table 3.

**Table 3:** Comparative overview of photolithography and additive manufacturing across key microfabrication performance metrics

Aspect	Photolithography	Additive manufacturing
Resolution and fidelity	Mature sub-micrometer (down to <100 nm in advanced variants) for planar patterns; exceptional lateral accuracy and smooth features—ideal for membranes, valves, gratings^188^	Typical hydrogel DLP/LCD lateral features ∼20–100 µm; advanced workflows show ∼75 µm laterals, ∼22 µm membranes; two-photon polymerization (2 PP) can reach <200 nm voxels but with limited throughput^189^
Throughput and scalability	Batch parallelization (whole-wafer exposure); fast for 2D arrays; excellent for mass fabrication of many identical devices^190^	Layer-by-layer, DLP/LCD scale favourably (full-layer projection), enabling thousands of devices per run; 2PP is slower, though parallelization strategies are emerging^191^
Geometry	Best for quasi-2D or multilayer (stacked) devices; 3D complexity requires alignment and bonding steps^192^	True 3D freedom (overhangs, internal channels, graded lattices) enables architected hydrogel actuators and complex microneedles^193^
Material palette	Photo-crosslinkable gels (PEGDA, pNIPAM, *etc.*) can be readily patterned; multilayer composites with controlled crosslink gradients by dose/mask design^194^	Broad and expanding: SLA/DLP resins for PEGDA/GelMA/HA-MA; custom inks for biocompatibility; 2PP-tailored photoresists including sugar-responsive systems for 4D actuation^195,196^
Preferred architectural domains	Microvalves, membranes, microchannels, photonic lattices, planar microgrippers; tight control of thickness and in-plane gradients for chemomechanics^197,198^	Complex microneedles, micro-robots, porous scaffolds; organ-on-chip with embedded structures; rapid iteration without masks^199,200^
Practical constraints	Requires masks/aligners and (typically) a cleanroom; superb repeatability once the process is set^188^	Maskless, CAD-to-part; consumer-grade LCD printers + tailored PEGDA inks now achieve high throughput at low cost; however, resin toxicity and post-cure steps need care^199,201^

## 6. Tug or drift? Navigating passive and active pathways in microdevice drug delivery

Miniaturization and developments in microdevices have paved the way for the development of biomedical microdevices with the potential to cure a wide range of acute and chronic diseases. New biomedical devices with micrometre characteristics can be manufactured and implanted through minimally invasive procedures to distribute therapeutic drugs in a regulated manner. Microdevices for controlled drug release are composed of various structural components, such as microchannels and micro-reservoirs, which store drugs. Additionally, microtransducers, including microsensors and microactuators, are integrated with microelectromechanical systems (MEMS) to augment the device's functionality.^202^ There can be passive and active microdevices, as elaborated further. Detailed discussions on the design, function, and attributes of such types are provided in the SI file (Section S5.1–S5.2). Consistent with the scope of this present review, our focus centres on stimuli-responsive microdevices, highlighting their mechanistic precision in delivering biomolecules in the domain of drug delivery and biomedical applications (Section 6).

## 7. Stimuli on cue: categorizing endogenous stimuli-activated microdevices for targeted therapeutics

**Fig. 5:**
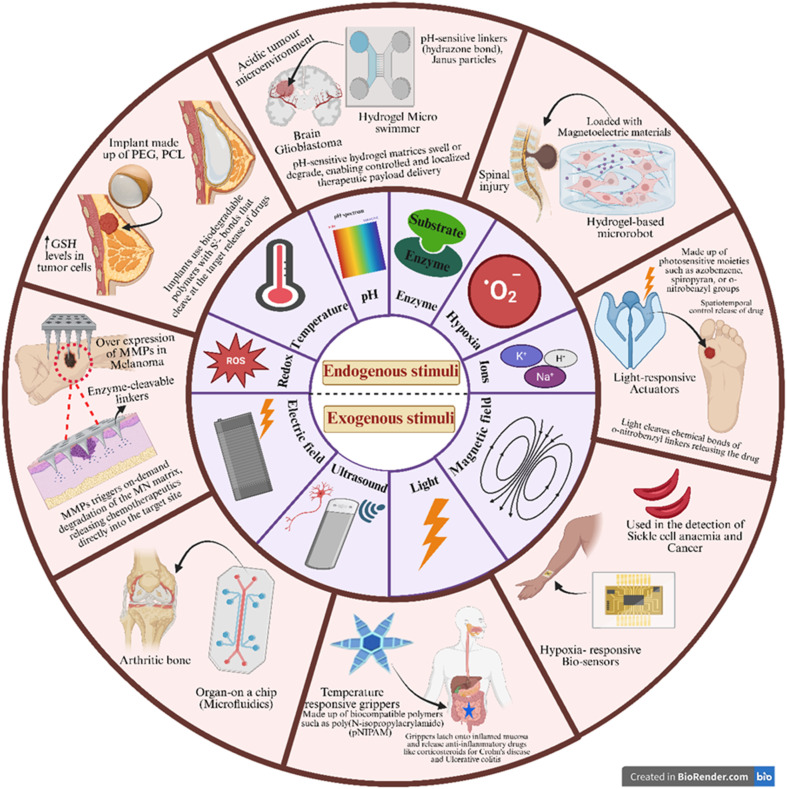
Schematic illustration of diverse endogenous and exogenous stimuli-responsive microdevices utilized in drug delivery and biomedical applications. This image has been created as a Creative Common using the BioRender software (https://www.biorender.com/).

Endogenous stimuli-responsive microdevices leverage intrinsic physiological and pathological cues within the body to achieve site-specific and controlled drug release. These devices are engineered using advanced techniques such as microfabrication, surface patterning, and microfluidics, and can be integrated with cellular or tissue models to enhance their functionality. Unlike exogenous triggers that rely on external interventions (*e.g.*, temperature, light, and magnetic fields), endogenous stimuli originate from the local microenvironment of diseased tissues, including variations in pH, enzyme activity, redox potential, hypoxic conditions, and ionic gradients (Fig. 5).^203^ By exploiting these inherent biochemical and physicochemical signatures, such microdevices enable highly selective therapeutic action, minimizing systemic exposure and improving efficacy. The following sections summarize key developments in microdevices designed to respond to these endogenous cues, highlighting their mechanisms and applications in precision drug delivery.

**Fig. 6:**
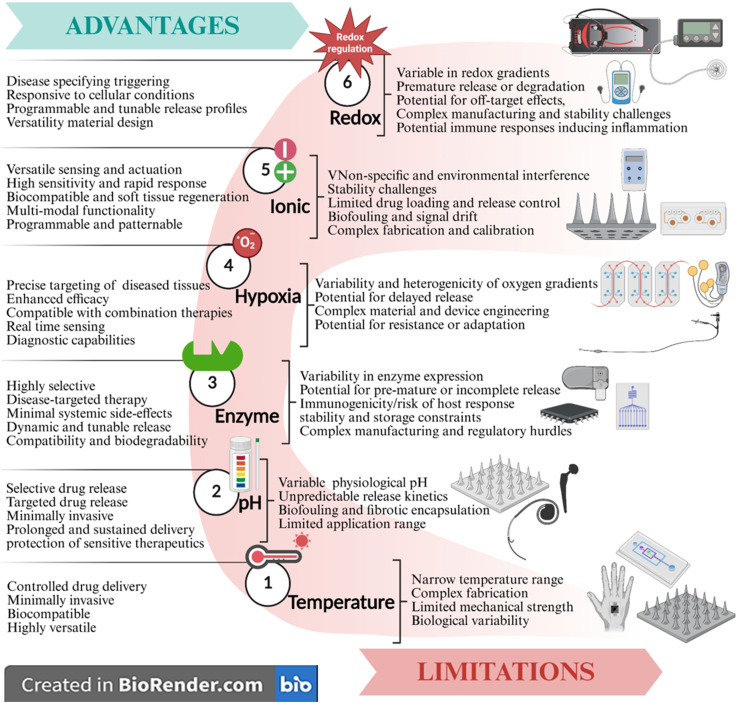
Advantages and limitations of endogenous stimuli-responsive microdevices. This schematic illustrates various internal triggers, including temperature, pH, enzyme, hypoxia, ionic, and redox, that operate on the principle of phase transition, swelling/degradation, enzymatic cleavage, by triggering drug release in low-oxygen environments, ion-sensitive polymers responding to ionic strength changes, and disulfide bond cleavage, respectively. This image has been created as a Creative Common using the BioRender software (https://www.biorender.com/).

Stimuli-activated microdevices represent a transformative frontier in precision drug delivery, enabling spatiotemporal control over therapeutic release through engineered responsiveness to physiological or externally applied cues. This section categorizes the diverse landscape of endogenous-responsive microdevices, delineating their design principles, activation mechanisms, and biomedical applications. Endogenous triggers, such as temperature, pH gradients, enzymatic activity, redox potential, and hypoxia, are harnessed to achieve site-specific release within pathological microenvironments (Fig. 6).^7^ Endogenous stimuli-based microdevices represent an innovative class of smart drug delivery systems that leverage internal physiological signals to achieve controlled and site-specific therapeutic release.

### 7.1. Temperature-responsive microdevices

Temperature is a unique stimulus that can be both endogenous, arising from localized hyperthermia in tissues such as tumors or infections, and exogenous, applied externally. Advances in biocompatible thermoresponsive polymers have enabled the design of intelligent drug-delivery nanocarriers with tunable release profiles.^204–206^ These systems can enhance therapeutic efficacy or spatially confine cytotoxic effects to target sites,^207,208^ and are increasingly integrated into multi-stimuli platforms, including dual (*e.g.*, temperature/pH) and triple-responsive (*e.g.*, thermo/pH/redox or glucose/pH/thermo) delivery systems.^209–213^

Vincenzo C. *et al.* conducted a study using superparamagnetic nanoparticles loaded in micro-carriers of thermosensitive and injectable biopolymers. This resulted in the creation of “smart” microdevices that could respond to an external magnetic field (EMF) by releasing co-encapsulated molecules with remote on–off control.^214^ The researchers proposed the use of Supercritical Emulsion Extraction Technology (SEET) to fabricate microcapsules with a core of poly-lactic-*co*-glycolic acid (PLGA) or poly-lactic acid (PLA) covered by carboxybetaine-functionalized chitosan (f-Chi). These microcapsules were loaded with paramagnetic nanoparticles (MAG) and water-soluble fluorescein (Fluo), which served as a fluorescent marker for release studies. The microcarriers had an average size of 800 ± 60 nm and an encapsulation efficiency of up to 90%. The f-chitosan coating resulted in a uniform functionalized surface with a thickness of 200 ± 50 nm, allowing for further chemical linkage. EDX-SEM and IR analyses confirmed effective dispersion and encapsulation of magnetite within PLGA and PLA microdevices fabricated *via* SEE. Surface charge measurements showed that magnetite was well encapsulated, as zeta potentials of uncoated carriers remained negative across pH levels, unlike free magnetite. Coating with f-chitosan significantly increased zeta potential to +26 ± 1.2 mV at pH 5.5, indicating successful surface modification. FE-SEM and TEM images revealed that the f-chitosan coating preserved the spherical shape of PLGA carriers while slightly roughening their surfaces. The study confirmed excellent dispersion of MAG within the biopolymer matrix, which exhibited responsiveness to EMF. Fluo was released over 3 or 5 days from PLGA or f-chipola microdevices into a PBS medium at 37 °C. Nearly 60% of Fluo was released on the first day, showing a typical burst release for water-soluble Fluo in submicron devices. From day two, release was sustained at ∼8% per day over five days. Applying an f-Chi coating halved the initial burst to 34% and slowed subsequent release to 5.5% per day, extending total release to 10 days. This controlled release was likely due to carboxybetaine in the coating, which traps solutes within its hydrated structure. Furthermore, remote on–off controlled release and tunability based on carrier composition were achieved by applying an alternating magnetic field (AMF). PLGA carriers released 40%, 30%, and 20% of Fluo in the first three AMF pulses, while f-ChiPLGA showed reduced release (36%, 28%, 10%), indicating the coating's dampening effect. PLA carriers released 25–18% in the first three pulses, followed by ∼10% in subsequent ones. Overall, the proposed method combines emulsification with supercritical fluid technology to efficiently produce engineered microcarriers with high encapsulation efficiency and well-dispersed magnetic nanoparticles. The addition of carboxybetaine-functionalized chitosan coating enhances controlled drug release and biocompatibility.

In another experiment conducted by Meng *et al.*, a microfluidic device was utilized to generate micro-HIFU (MHIFU). This device controlled the release of drugs from temperature-sensitive liposomes (TSL) and assessed the thermal and mechanical effects of ultrasound on cellular drug uptake and apoptosis.^215^ By adjusting the input electrical signal, the sample temperature was maintained at desired levels of 37 °C, 42 °C, and 50 °C with a deviation of ±0.3 °C. The results obtained from flow cytometry revealed a significant increase in apoptosis when using MHIFU sonication for drug delivery compared to incubation alone at an elevated temperature of 42 °C. At a constant fluid temperature of 37 °C, no significant apoptosis was observed across groups, with cell viability exceeding 92%. However, at 42 °C, late apoptosis in the first group increased to 17.8%, markedly higher than in the second (3%) and third (3.5%) groups. The second group maintained 94.3% viability, indicating that short MHIFU exposure alone did not compromise cell health. The minimal apoptosis in the third group at 42 °C is likely due to the short heating duration (3 min). Notably, dead cells in the first group rose to 5.3%, suggesting that MHIFU-assisted TSL delivery accelerates apoptosis. Overall, MHIFU treatment at 42 ± 0.3 °C resulted in a 5.9-fold increase in late apoptotic cells compared to the control group maintained at 37 °C. At 50 °C, apoptosis levels increased significantly across all groups, confirming that elevated temperature is the primary driver of cell death, independent of TSL involvement. Additionally, atomic force microscopy detected an increase in squamous and protruding structures on the cell membrane surface after MHIFU irradiation of TSL. These findings demonstrated that MHIFU allows for real-time monitoring of interactions between ultrasound and cells, which is not possible with routine HIFU treatment.

Cell-mediated drug delivery systems employ living cells as carriers for the precise administration of therapeutics. One of the system's features is the integration of cells with disk-shaped microparticles, producing cell-microdevice complexes that offer distinct advantages over their counterparts. Human mesenchymal stem cells (hMSCs) have been thoroughly investigated as therapeutic agents and employed as vehicles for drug-loaded nanoparticles or other functional nanoparticles. To this end, Xia J. *et al.* introduced a novel microdevice-based drug delivery system mediated by hMSCs.^216^ This study demonstrated that microdevices could be affixed to hMSCs in a controlled and adaptable manner. The resulting hMSC-microdevice complexes remained stable during culturing and trypsinization, and the attachment of the microdevices did not affect the viability or proliferation of the hMSCs. Furthermore, cultured microdevice-bound hMSCs preserved their capacity to move across a planar surface, generate a spheroid, and actively disassociate from the spheroid. Taken together, the results suggested that the fabricated microdevice-based hMSC-mediated technology holds the potential for advancement into a clinically feasible drug delivery system.

One of the interesting works reported by Ghosh A. *et al.* involved the creation of theragrippers equipped with multiple sharp microtips, like the hookworm, to ensure effective attachment to the gastrointestinal (GI) mucosa.^217^ The researchers reported that these GI parasite-inspired theragrippers consisted of thick, rigid segments and residually stressed bilayer hinges, covered with a thermosensitive wax layer. These theragrippers remained in the GI tract of live animals for 24 h by autonomously latching onto the mucosal tissue. Notably, a significant six-fold increase in the elimination half-life was observed when delivering a model analgesic, ketorolac tromethamine, using theragrippers. These findings demonstrated that shape-changing and self-latching microdevices can improve the effectiveness of prolonged drug delivery. The researchers also noted that the thermoresponsive nature of the theragrippers necessitated refrigeration for storage, especially in tropical regions where temperatures can exceed 37 °C. The extended-release delivery of drugs in the GI tract has greatly simplified drug administration and improved adherence to therapeutic regimens. Nevertheless, obstacles persist due to GI motility clearance, complicating device retention and prolonged medication release.

**Fig. 7:**
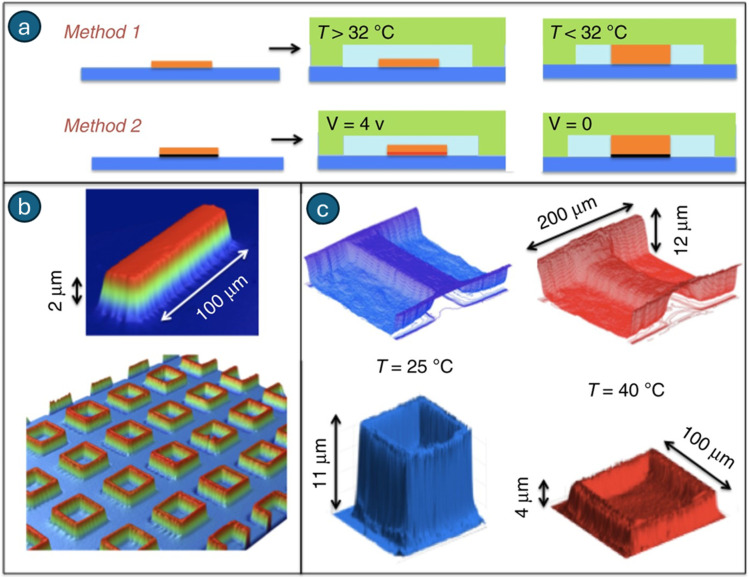
Microfabrication technique: hydrogel structures are deposited and grafted or patterned onto the substrate. Method 1 induces global temperature variations, while method 2 actuates hydrogel structures individually through the deposition of patterned chromium/gold layers. Then, the system is closed using an NOA or PDMS structure. At temperatures below the lower critical solution temperature (*T* < LCST), the hydrogel exhibits swelling. Increasing the temperature beyond the LCST or heating the metal layer causes the hydrogel to collapse (a). Diverse hydrogel patterns affixed to surfaces, visualized using interferometric microscopy: a singular patch and an array of square microcages. The widths of the structures (patch and microcage walls) are 10 µm, the horizontal dimensions of the cage are 200 µm × 200 µm, and the wall height is 2 µm (b). *In situ* topographic views of valves and cages depict the heating conditions. At 20 °C, the hydrogel is expanded, the valve is sealed, and the cage walls are elevated. At 40 °C, the hydrogel is contracted, the valve is open, and the chamber walls are positioned low (c). Adapted with permission.^219^ Copyright 2018, Springer Nature.

Microcages are utilized in pharmaceutical delivery to facilitate the controlled transport and release of medications. These function as containers for pharmaceuticals, facilitating focused administration and regulated release. Certain microcages can be activated by stimuli to open and dispense pharmaceuticals. Microcages can exhibit mechanical robustness and be integrated into implants. Certain microcages can accommodate substantial pharmaceuticals, including solid nano/microparticles.^217,218^ To this end, D'eramo *et al.* reported a hydrogel-based microfabrication approach for developing microdevices containing up to 7800 individually actuated microcages capable of solute sequestration and release. Each microcage is equipped with a thermally responsive valve exhibiting rapid and reproducible switching dynamics, with average opening and closing times of 250 ms and 600 ms, respectively (Fig. 7).^219^ The findings demonstrated sequential transport of two fluids, 0.8 mmol per L fluorescein and pure water, into a common channel *via* two valves actuated with a 180° phase shift at 2 Hz, producing fluorescein waves that propagate downstream. The wave amplitude exhibited a low-pass response with a cutoff frequency of 5 Hz. Valve switching times were quantified from interface displacement data, yielding a closing time constant of 0.6 ± 0.1 s and an opening time of 0.25 ± 0.15 s. These sub-second actuation times are well-suited for biotechnological applications and align with theoretical diffusion-based estimates. Accordingly, this approach was exemplified in single-cell handling and the nucleic acid amplification test (NAAT) for the Human Synaptojanin 1 gene, implicated in various neurological disorders, including Parkinson's disease. The efficacy of the temperature-responsive hydrogels presented in this work indicated that, coupled with their reasonable costs, these hydrogels could serve as a viable alternative to the existing actuation or manipulation methods employed in microfluidics, specifically pressure-actuated polydimethylsiloxane (PDMS) valves and droplets. Overall, very large system integration in isothermal systems, where all cages were actuated simultaneously, was feasible by minimizing the dimensions of the hydrogel valves or cages to 5 µm. However, the autonomous actuation of cages complicates the scenario; yet the prospect of achieving densities of thousands of cages per cm^2^, indicating a high throughput, remains feasible.

**Fig. 8:**
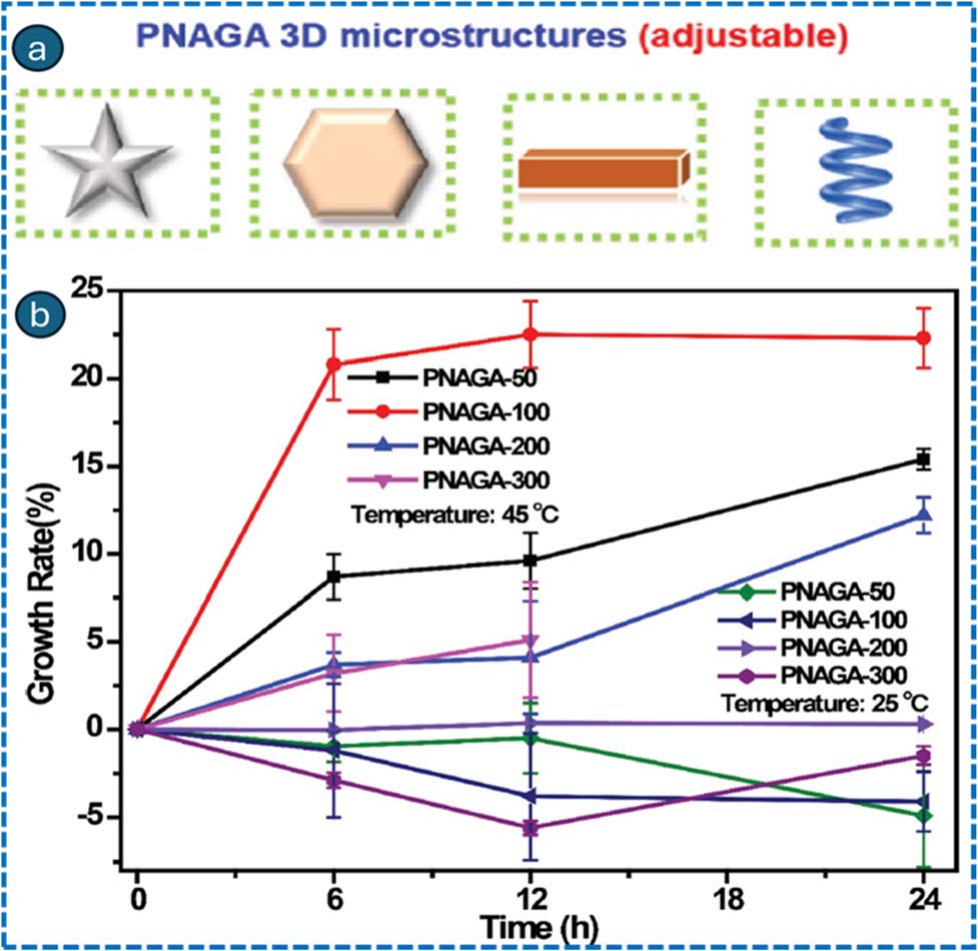
Schematic representation of 3D printed PNAGA microstructures (a), and thermosensitive properties of 3D-printed PNAGA-based microrobots at 25 °C and 45 °C (b). Adapted with permission.^45^ Copyright 2023, AccScience Publishing.

Temperature-responsive hydrogels are being progressively employed and studied due to their potential applications in the biomedical sector. In a study reported by Zhou *et al.*, thermosensitive poly-*N*-acryloyl glycinamide (PNAGA) hydrogel-based microrobots were prepared utilizing advanced two-photon polymerization printing techniques.^45^ The thermosensitive performance of *N*-acryloyl glycinamide (NAGA) was demonstrated to be concentration-dependent, and the underlying mechanism was investigated. PNAGA-100 exhibited rapid swelling behaviour at 45 °C, achieving a growth rate of 22.5%, the highest among the PNAGA hydrogels. A drug release study of PNAGA-100-based thermosensitive hydrogels was performed. The microrobots exhibited a greater drug release at 45 °C compared to 25 °C, underscoring their significant potential for application in medication delivery within the human body (Fig. 8). Additionally, PNAGA-100-based thermosensitive microrobots can navigate the predetermined path under the influence of a magnetic actuator following incubation with Fe@ZIF-8 crystals. Overall, biocompatible thermosensitive magnetic microrobots offer novel possibilities for advanced biomedical applications with high performance.

**Fig. 9:**
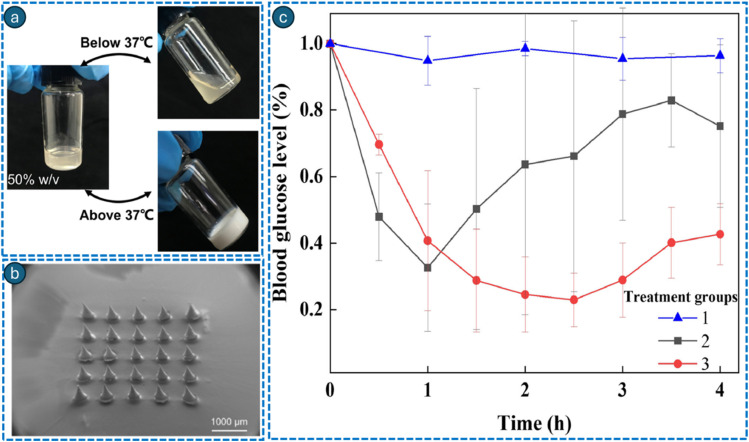
Visual morphological examination of the GP solution (50% w/v) at ambient temperature and 37 °C (a). Scanning electron microscopic images captured for drug-loaded MNs (b). Graphical representation of the blood glucose levels of group 1 (control mice), group 2 (gelatin-fabricated RS-MNs-treated animals), and group 3 (RS-GP-MNs-treated mice) (*n* = 3), administered with 0.4 IU of insulin. The blood glucose levels of the diabetic model mice were standardized to the value of 0 h (c). Adapted with permission.^230^ Copyright 2022, Elsevier.

Microdevices, including micron-sized impregnated needles attached to the polymeric substrate, have been shown to penetrate the stratum corneum to locally deliver bioactive compounds into the epidermis and/or superficial dermis.^220,221^ Microneedles (MNs) present an attractive way of delivery as this technique is painless and can potentially be self-administered. Recently, stimuli-responsive microneedles, often constructed from polymeric matrices, have been developed to enhance and regulate the release of payloads.^222,223^ Thermoresponsive microneedles are fabricated from materials having a low melting point (<55 °C), allowing them to shift from solid to liquid upon exposure to a heat source. These MNs can absorb heat from two sources to facilitate phase transition. The initial heat source originates from a thermal heater. Polymers like polycaprolactone, which have a low melting point of 50 °C, experience a phase transition that facilitates drug release.^224^ Such polymers often include chitosan and β-sodium glycerophosphate (β-GP), which were added to MN formulations to improve solubility.^225^ When the temperature exceeds the phase transition temperature of chitosan and β-GP, the hydrophobicity of chitosan molecules increases and hydrogen bonding weakens, resulting in a reversible gel formation. This characteristic can hasten the disintegration of MNs made from dextran. Other thermosensitive MNs may use amphiphilic block copolymers such as polyethylene oxide–polypropylene oxide (poloxamers or Pluronic, PPO–PEO–PPO). These amphiphilic block copolymers can undergo thermal sol–gel transformation.^226–228^ Hydrogels made from thermosensitive poly(lactic-*co*-glycolic acid)–polyethylene glycol-poly(lactic-*co*-glycolic acid) copolymers can transfer plasmid deoxyribonucleic acid to the skin *via* MNs.^229^ Li *et al.* reported the use of gelatin grafted with carboxylic end-capped poly(*N*-isopropylacrylamide) (PNIPAm) as the matrix material to fabricate a physical entanglement crosslinked hydrogel MN patch that can control drug release after skin application.^230^ PNIPAm can switch from a linear state to a coiled state with temperature. The quick-separating method and thermosensitive hydrogel delivered drug-loaded MNs to the skin in seconds. The hypoglycaemic effect in diabetic mice was distinctly regulated by insulin release *via* RS-GP-MNs, in contrast to the MNs fabricated from unmodified gelatin (Fig. 9).

Author's critical perspective: across these modalities, the strongest efficacy signals emerge when responsive materials are coupled to precisely titratable external triggers within a narrow mild-hyperthermia band (≈41–43 °C). Magnetic and ultrasound fields enable on–off dosing, but their clinical utility depends on thermal dosimetry discipline (CEM43) and biocompatible carrier designs that suppress early burst without sacrificing response agility. Mechanical and biological residency strategies (theragrippers, hMSC carriers) can extend exposure but introduce safety and regulatory tradeoffs; mucosal microtrauma and immunogenicity must be quantified, and biodegradability assured. Among the platforms, thermoresponsive MNs and SEET-fabricated microcarriers appear closest to translational viability, given their manufacturability and control over release. Future work should adopt standardized thermal metrics, *in vivo* burst profiling with clinically relevant payloads, and head-to-head comparisons under matched thermal doses to resolve the current ambiguity between heat-mediated cytotoxicity and triggered drug action. Taken together, in the studies mentioned above, researchers have examined the development, manufacturing, and applications of these miniature devices across different fields. The progress in thermosensitive microdevices involves combining materials that change properties based on temperature, allowing for accurate reactions at temperature levels. The discussed studies overall aimed to harness the potential of thermosensitive microdevices in fields such as healthcare, where they can be used for precise drug delivery or temperature-dependent diagnostic tests. Additionally, these microdevices have shown promising outcomes in environmental monitoring through sensing applications that use their temperature-detecting capabilities.

### 7.2. pH-responsive microdevices

Controlled drug-delivery systems enable administration of therapeutics at predefined rates and durations, surpassing conventional formulations; however, several clinical conditions, such as diabetes and cardiac rhythm disorders, still require improved spatiotemporal control of dosing. Ideally, drug release should be synchronized with physiological cues, including local pH variations. pH-responsive microdevices address this need by exploiting pathological pH differences, such as the acidic microenvironment of tumors or inflamed tissues, to trigger site-specific drug release. These systems typically employ polymers that undergo swelling or structural transitions under acidic or basic conditions, enabling regulated and targeted therapeutic delivery.^231,232^ Several key mechanisms are involved in the mechanism of action of pH-responsive microdevices.^233,234^ A decrease in pH induces protonation of ionizable functional groups within the polymer network, altering the local chemical environment and triggering structural changes. Protonation typically increases polymer hydrophilicity, leading to network swelling and enhanced permeability, which facilitates the diffusion of encapsulated drugs. Concurrently, acid-labile cross-links may dissociate under acidic conditions, further loosening or collapsing the polymer matrix. The combined effects of swelling and bond dissociation enable rapid, localized, and efficient drug release, thereby enhancing therapeutic efficacy while minimizing off-target exposure.

Environmentally responsive hydrogels are inherently suitable for use in microfabricated devices. The polymerization of necessary monomers and crosslinking agents can be achieved through free-radical polymerization utilizing UV light, thereby allowing for the use of photolithographic techniques to pattern these materials at the microscale.^235,236^ VanBlarcom *et al.* fabricated poly(methacrylic acid) crosslinked with different molar percentages of polycaprolactone diacrylate to make pH-responsive, biodegradable hydrogels.^237^ The equilibrium swelling properties of pH-responsive materials were examined. Methods to use these new hydrogels as silicon-based microsensor sensing components were investigated. Photopolymerization atop silicon microcantilever arrays produced extremely thin hydrogel films that converted pH-responsive volume change into optical signals. Organosilane chemistry covalently bonds the hydrogel to the silicon beam. As the hydrogel inflated, the silicon–hydrogel surface stress deflected the beam downward. The sensor has a maximum sensitivity of 1 nm/5.7 × 10–5 pH unit. The sensors' remarkable sensitivity was maintained in protein-rich liquids to imitate biological circumstances. The existing theory was examined and developed to predict composite cantilever beam deflection. Likewise, in another study, a microdevice for drug administration that incorporates pH-responsive nano-hydrogel composite membranes acting as an intelligent nanovalve was reported.^238^ The polymeric microdevices were monolithic and did not necessitate external control circuitry or Fntary components for regulating drug-release rates. pH-responsive nanoparticles were synthesized and incorporated into a composite membrane. The resultant pH-responsive composite membrane was combined with PDMS micro-reservoirs to create the proof-of-concept microdevices. The *in vitro* release characterization of the microdevices demonstrated a significant increase in the release rate of vitamin B_12_ when the local pH was reduced from 7.4 to 4. Modifying nanoparticle proportions, the morphology and dimensions of drug reservoirs, and drug loading concentrations can provide intricate drug release patterns in response to localized pH fluctuations, serving as a foundational technology for sophisticated drug delivery systems.

**Fig. 10:**
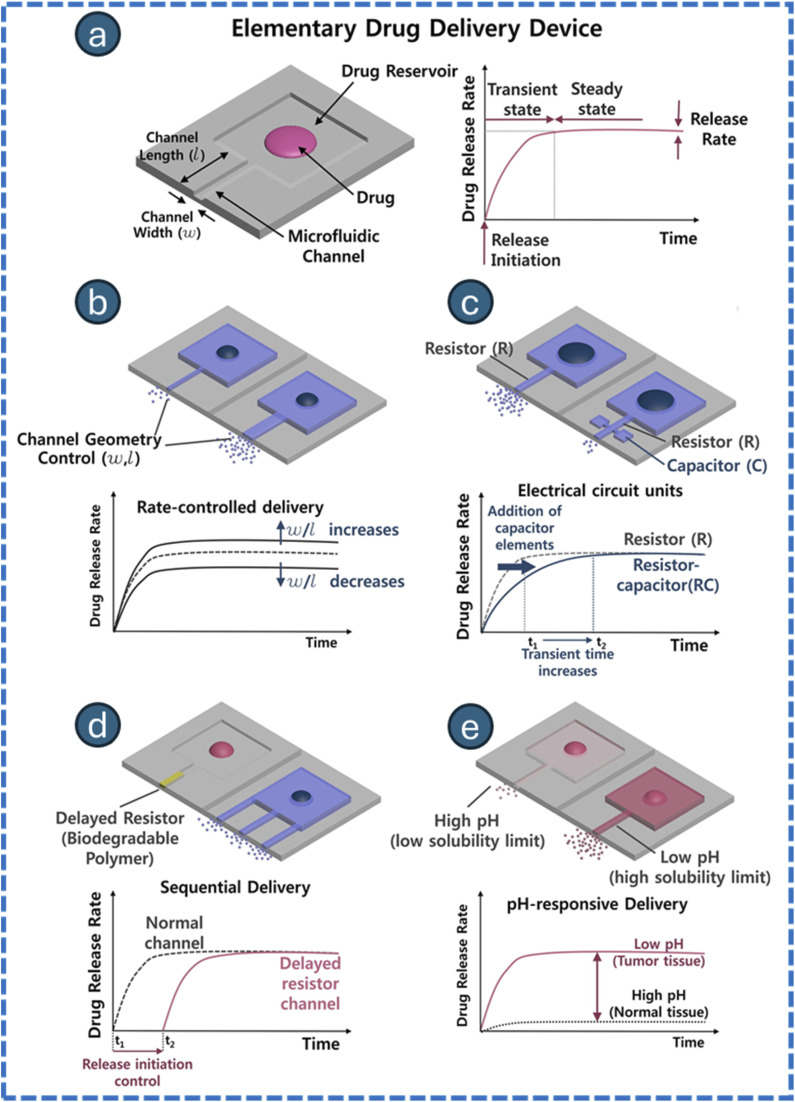
Schematic representations of microfluidic drug delivery regulation and associated release characteristics. An elementary microfluidic unit consisting of a drug reservoir and a microfluidic release channel (a). The release rate can be regulated by channel geometry, including channel length (*l*) and channel width (*w*) (b). Elementary drug delivery units were comparable to electrical circuit components such as resistors (*R*) and capacitors (c). The array of microfluidic drug delivery devices enables sequential (d) and pH-responsive (e) distribution, together with the associated release profile. Adapted with permission.^242^ Copyright 2018, Elsevier.

More advancements in the delivery of peptide and protein therapeutics are needed in controlled drug delivery. A feedback mechanism known as “homeostasis” regulates the emergence of many bioactive peptides in the body to preserve a proper metabolic balance. It would be helpful to have a system that could identify the sickness signal, measure its strength, and then release the right number of biomolecules in response. pH-responsive microdevices have great promise in improving the safety and effectiveness of medication delivery in therapeutics.^239^ For example, these microdevices can traverse the stomach's acidic milieu to deliver the therapeutic payload in the intestines' more neutral pH environment, thereby reducing systemic exposure and side effects in the treatment of gastrointestinal illnesses such as peptic ulcers or inflammatory bowel disease.^240^ Similarly, pH-responsive microdevices can be used in cancer therapy to release chemotherapeutics into tumor tissue while protecting healthy cells. This maximizes therapeutic efficacy and minimizes off-target limitations, as solid tumors frequently have acidic microenvironments.^241^ Yang D. *et al.* published a study that created microchannel-based drug delivery devices capable of precisely controlling release rates through the design of microchannel geometry.^242^ The devices were submerged in a release medium, and time-lapse photography showed a significant relationship between release rates and channel shape. The study also examined the capacitor effect using FEM modeling, proving that adding capacitors could alter transient conditions. The anti-cancer drug was successfully delivered at a low pH level, and HeLa cell viability assays verified a cutoff pH at which drug release was activated. Therapeutic molecules with pH-dependent solubility could be released through channels reacting to environmental pH, which has applications in cancer treatment. The proposed soft microrobot carrying a trapped drug microbead can locate a tumor, conduct its unfolding motion, and release the trapped drug microbead. This approach could be used in targeted drug delivery for cancer cell diagnostics and treatment (Fig. 10).

Li *et al.* developed a magnetically activated pH-responsive hydrogel soft microrobot for drug targeting and delivery.^243^ The microrobot, made of biocompatible PEGDA and PHEMA bilayers and Fe_3_O_4_ nanoparticles, featured eight radial arms and can be used to target docetaxel microbeads (PCLDTX). After treatment, the viability of 4T1 breast carcinoma cells dropped to 70.25 ± 1.52%. The microrobot's ability to locate tumors and release trapped drug microbeads is expected to be used in targeted drug delivery for cancer cell diagnostics and treatment. Other medical procedures that require various control methods for optimal efficiency and precision can be addressed using this strategy.

For numerous medical therapies, especially in oncology, it is essential to create a biocompatible microscale device capable of transporting an adequate quantity of a drug and administering it to specific target locations. Chemically powered micromotors have been utilized in live animal therapy; however, many exhibit poor biodegradability *in vivo*, potentially leading to toxicity and adverse effects. To this end, Zhou *et al.* fabricated and reported a microdevice including a poly(aspartic acid) (PASP) microtube, a thin iron intermediate layer, and a zinc core at different pH. This device can be powered by gastric acid as a fuel source.^244^ Following the adsorption of doxorubicin onto a PASP surface, the micro rocket could deliver drugs, penetrate the gastrointestinal mucus gel layer, and enhance drug retention in the stomach without eliciting a significant adverse response. All components of the micro rockets were biocompatible and biodegradable, capable of being rapidly dissolved by gastric acid or proteases within the digestive tract. Notably, this method may facilitate targeted drug delivery for cancer cell diagnosis and treatment, providing enhanced precision and efficiency in on-demand medical procedures. Taken together, micro rockets constructed from poly (amino acid)s are hypothesized to enhance the biomedical uses of micro- and nanomotors. In another study by Yan Y. *et al.*, a star-block copolymer was fabricated to release anionic therapeutics at physiological pH.^245^ Upon the PEI core, PEI-(PLL-*b*-PEG), the outer shell's PEG and the inner shell's PLL block were grafted. The polymerization of benzyloxycarbonyl-l-lysine *N*-carboxy anhydride induced ring-opening by PEI's peripheral primary amine groups. After that, activated PEG 4-nitrophenyl carbonate was applied to the surface to modify it. Historically, anionic dyes like rose Bengal and methyl orange, as well as model drugs like diclofenac sodium, could be released at high pH levels of 10.0–11.0 and low pH levels of 2.0–3.0 when encapsulated in this star-block copolymer. Overall, the effective encapsulation of PEI-*g*-(PLL-*b*-PEG) and its sustained, pH-responsive release characteristics may be utilized for the regulated delivery of anionic drugs.

Bacterial infection is currently considered to be one of the major reasons that lead to the failure of guided bone regeneration (GBR) therapy. Under normal conditions, the pH is neutral, while the microenvironment will become acidic at the sites of infection. To this end, Chen *et al.* reported an asymmetric microfluidic/chitosan device that can achieve pH-responsive drug release to treat bacterial infection and promote osteoblast proliferation at the same time.^48^ On-demand release of minocycline relies on a pH-sensitive hydrogel actuator, which swells significantly when exposed to the acidic pH of an infected region. The PDMAEMA hydrogel had pronounced pH-sensitive properties, and a large volume transition occurred at pH 5 and 6. Over 12 h, the device enabled minocycline solution flow rates of 0.51–1.63 µg h^−1^ and 0.44–1.13 µg h^−1^ at pH 5 and 6, respectively. The asymmetric microfluidic/chitosan device exhibited excellent capabilities for inhibiting *Staphylococcus aureus* and *Streptococcus mutans* growth within 24 h. It had no negative effect on the proliferation and morphology of L929 fibroblasts and MC3T3-E1 osteoblasts, which indicated good cytocompatibility. Therefore, such a pH-responsive drug-release asymmetric microfluidic/chitosan device could be a promising therapeutic approach in the treatment of infectious bone defects.

**Fig. 11:**
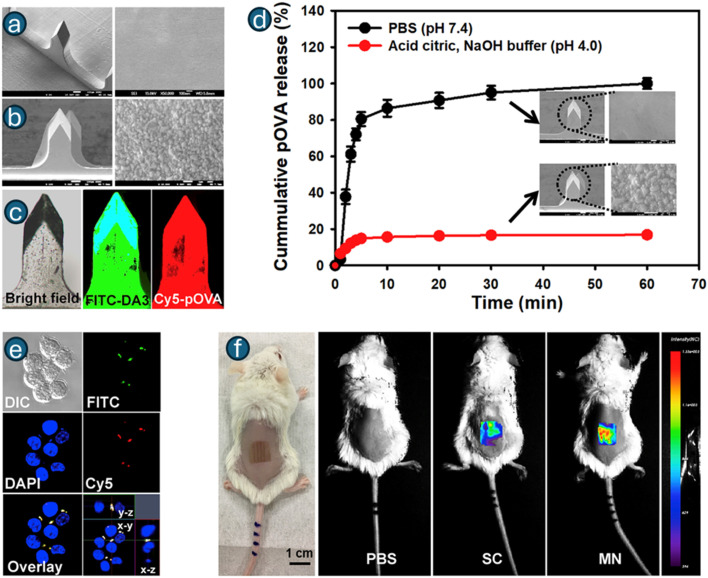
Schematic representation of a microfluidic device designed for evaluating the swelling and deswelling characteristics of pH-responsive microspheres (a). Micrograph depicting the ‘On’ state of the microvalve (b) and the ‘Off’ state of the microvalve (c). (a) Three-dimensional conceptual schematic illustrating the contraction motion of the microsphere within the entrap posts; valve in the ‘On’ position (d) and valve in the ‘Off’ state (e). SEM image of the microsphere situated within the entrap posts (f). Adapted with permission.^248^ Copyright 2018, Elsevier.

As mentioned earlier, for the thermos-responsive MNs, likewise, pH-responsive microneedles are composed of polymers that react to fluctuations in the surrounding pH by means of degradation, swelling, or collapse. These materials generally contain hydrophilic and ionic functional groups inside the polymer segments. Variations in pH across various organs or reduced pH at the locations of chronic wounds, inflammation, or cancer have been thoroughly investigated as a catalyst for the development of pH-sensitive drug delivery devices.^246,247^ Duong *et al.* reported advanced delivery of DNA vaccine utilizing nanoengineered DNA vaccines affixed to MNs coated in layers with ultra-pH-responsive OSM-(PEG-PAEU) and the immunostimulatory adjuvant poly(I:C), a synthetic double-stranded RNA (Fig. 11).^248^ The transcutaneous application of MN patches on murine skin penetrated the stratum corneum with minimal cellular injury; subsequent disintegration at the immune-cell-dense epidermis/dermis facilitated the release of adjuvants and DNA vaccines, due to the highly acute pH-responsive characteristics of OSM-(PEG-PAEU). The administered adjuvant and DNA vaccine can promote dendritic cell maturation and stimulate type I interferons, resulting in the production of antigen-specific antibodies that facilitate antibody-dependent cell-mediated cytotoxicity (ADCC) and enable CD8+ T cells to eliminate cancer cells. Remarkably, the transcutaneous administration of a smart vaccine formulation in mice produced threefold higher levels of anti-OVA IgG1 serum antibodies and a threefold increase in cytotoxic CD8+ T cells compared to the soluble DNA vaccine formulation. The formulation eliminated murine B16/OVA melanoma tumours in C57BL/6 mice by synergistically activating antigen-specific ADCC and cytotoxic CD8+ T cells. The strategic use of vaccine and adjuvant poly(I:C) in microneedles elicits both humoral and cellular immunity, presenting a promising vaccine technique that demonstrates enhanced efficacy, compliance, and safety. In a different study, a topical dissolving MN device with AIEgen (NIR950) was used to treat malignant skin tumour melanoma. The nanoprecipitation approach was used to prepare NIR950-loaded polymeric micelles (NIR950@PMs) to enhance the solubility.^249^ The micelles were coated on MN needle tips (NIR950@PMs@MN). NIR950@PMs' emission intensity did not decrease after an hour of laser irradiation. In an acidic tumour microenvironment, pH-responsive micelles can be protonated for intracellular uptake. Dissolving MN allowed NIR950@PMs to rapidly collect at the tumour location and attain a laser-killing temperature. Low-dose NIR950@PMs@MN could destroy melanoma tumors with one dosage and laser irradiation. This dissolving MN system loaded with NIR950 showed excellent photostability and photothermal action, suggesting therapeutic promise for superficial tumour therapy.

Zhang *et al.* fabricated and reported a pH-responsive MN patch for persistent incisional analgesia, followed by surgery.^112^ The MN patch had a 10 × 10 MN array in a 0.5 cm^2^ area, each having a complete core–shell structure and a needle height of ≈850 µm. Mechanical testing using a force measurement system demonstrated that each NaHCO_3_@CSMN loaded with ropivacaine (RopC) could withstand forces up to 1.3 ± 0.2 N before deformation, significantly surpassing the 0.1 N threshold required for effective skin penetration. The water-soluble backing layer dissolved quickly, allowing MNs to be implanted in 15 min. Shell rupture occurred when the pH-reactive media in the MN shell reacted with the surgical site's acidic microenvironment (pH 6.8–7.0). Encapsulated RopC microcrystals were released, which prolonged local anaesthesia duration to almost 72 h at 100 mg mL^−1^. The MN patch intelligently regulated medication release based on postoperative pain behaviour and surgical incision microenvironment pH. Overall, this responsive feature prolonged and personalized analgesia, overcoming the limitations of intrusive postoperative pain therapies. In another work on pH-responsive MNs, Ullah *et al.* fabricated porous polymer coatings on MNs that could automatically release therapeutics in response to wound pH to overcome the necrotic tissue barrier to improve antibiotic penetration without discomfort.^250^ The model drug was packed into the pores of the porous polymer film using aqueous gelatin porogen and covered with Eudragit S100 to prevent drug leakage and provide wound pH-responsive drug release. In rat and porcine skin, this formulation showed wound pH-sensitive drug release. MN release in test media was minimal at healthy skin pH (pH 4.5). Drug release increased significantly in MNs exposed to a wound pH of 7.5. The study proved that MNs with coating materials and antimicrobials can improve wound infection treatment and offer great potential for further development.

**Fig. 12:**
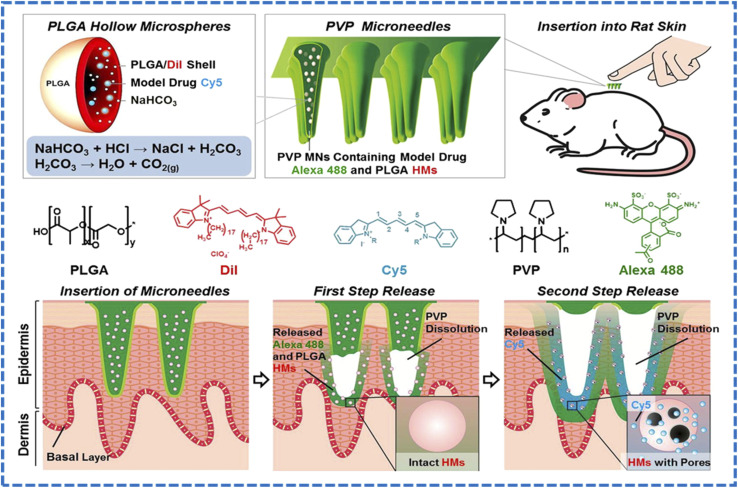
A schematic representation of the PVP MN arrays incorporating pH-responsive PLGA HMs and their mechanism for the sequential transdermal codelivery of two distinct model pharmaceuticals, Alexa 488 and Cy5. Adapted with permission.^251^ Copyright 2012, Elsevier.

Ke *et al.* introduced a method for the sequential transdermal co-delivery of two model drugs, Alexa 488 and Cy5, utilizing polyvinylpyrrolidone (PVP) MNs embedded with pH-responsive poly(d,l-lactic-*co*-glycolic acid) hollow microspheres (PLGA HMs) (Fig. 12).^251^ The MN system exhibited green fluorescence from Alexa 488 in PVP MNs, red fluorescence from the DiI-labeled PLGA shell of HMs, and cyan fluorescence from Cy5 in their aqueous core. The assembled MN arrays facilitated the localization of HMs and the monitoring of model drug release profiles inside skin tissues. The primary element of this system was NaHCO_3_, which can be readily integrated into HMs. Upon treatment of HMs with an acidic solution (mimicking the skin pH environment), protons (H+) can swiftly permeate the free space within the PLGA shells, reacting with NaHCO_3_ to generate a substantial quantity of CO_2_ bubbles. This process induced pressure within the HMs, forming pores in their PLGA shells and facilitating the release of the encapsulated Cy5. The MNs exhibited sufficient strength to be placed into rat skin without fracturing. The PVP MNs were rapidly dissolved within minutes, and Alexa 488, along with HMs, was effectively delivered into the tissues. Upon entering the acidic milieu of the skin, the liberated HMs commenced the release of Cy5 and subsequently disseminated into the adjacent tissues, marking the second phase of drug release. Taken together, this method can be employed clinically to sequentially and transcutaneously co-deliver a wide array of pharmaceuticals.

Author's critical perspective: pH-responsive microdevices show the greatest translational promise when biological pH gradients are paired with second-mechanism safeguards, mechanical residency (microneedles), anti-fouling skins (zwitterionic brushes), or guided placement (magnetics). While thin-film hydrogels and nanovalves offer precise, electronics-free control, their *in vivo* value hinges on fouling resistance and stability under pH cycling. Gas-mediated poration and micro-rockets provide sharp actuation but require comfort and safety envelopes for pressure and propulsion. Among platforms, pH-responsive microneedles and monolithic composite-membrane reservoirs are nearest to clinical scale, given manufacturability and clear functional endpoints (*e.g.*, 72-h analgesia, vaccine immunogenicity). Future studies should map real tissue pH landscapes, link trigger kinetics to PD outcomes, and standardize tests for biofouling and mechanical fatigue to resolve current ambiguities between elegant release curves and clinically meaningful benefit.

### 7.3. Enzyme-responsive microdevices

Enzyme-responsive microdevices are advanced drug-delivery platforms that exploit the catalytic specificity of dysregulated enzymes present in pathological tissues to achieve site-selective therapeutic release. By incorporating enzyme-labile linkers or enzyme-degradable polymer matrices, these microdevices remain stable under physiological conditions yet undergo localized degradation in environments enriched with proteases, glycosidases, or phosphatases.^252,253^ This strategy enables controlled, on-demand drug release within diseased sites, such as tumors, inflamed tissues, or infection foci. Their microfabricated architecture further supports precise dosing, reduced systemic exposure, and minimally invasive administration, positioning enzyme-responsive systems as promising tools for targeted and efficacious therapy.

The findings from Sengupta *et al.* demonstrated that enzymes immobilized on surfaces can act as micropumps when their substrates are present, even in the absence of adenosine triphosphate.^254^ The fluid-pumping velocity exhibited a concentration- and reaction-rate-dependent enhancement, governed by catalytic activity. Directional transport was driven by density gradients that induced convective flow, a consequence of localized catalysis. These micropumps further enabled precise spatial and temporal regulation of fluid movement, facilitating controlled delivery of colloidal particles and small-molecule cargo. Lastly, prototype enzyme-driven systems capable of self-propelled molecule and protein delivery in response to targeted chemical signals, such as glucose-induced insulin release, were demonstrated. The hydrogel leached dye molecules passively in the absence of a substrate, although dye release increased with substrate concentration. This was attributed to the enzymatic fluid pumping. At varied glucose concentrations in sodium acetate trihydrate buffer (pH 5.23), GOx-immobilized hydrogels released insulin. Increases in glucose concentration in the surrounding fluid increased hydrogel insulin release.

Jang *et al.*, in a distinct study, explored a multiplexed enzyme assay implemented within a microfluidic platform utilizing shape-coded poly (ethylene glycol) (PEG) hydrogel microparticles.^255^ The device architecture comprised two serially connected patterning chambers and a microfilter-integrated detection chamber, linked *via* a Y-shaped microchannel. Enzyme-loaded hydrogel microparticles of distinct shapes and sizes were fabricated in the patterning chambers through photolithography and subsequently transported to the detection chamber *via* pressure-driven flow. Sequential bienzymatic reactions, glucose oxidase (GOX) with peroxidase (POD), and alcohol oxidase (AOX) with POD, were successfully carried out using Amplex Red as the fluorescent reporter. By encoding GOX and AOX within uniquely shaped microparticles, individual enzymatic activities were readily distinguished, enabling simultaneous and interference-free detection of glucose and ethanol (1.0–10.0 mM) using a single fluorescence indicator within a unified detection zone. Overall, by randomly assembling tailored microparticles embedded with specific receptor molecules, the developed method can facilitate the simultaneous detection of multiple analytes, well beyond binary systems, within a single assay environment. An engineered glucose-responsive, implantable microdevice for closed-loop insulin delivery was fabricated, and its performance was evaluated through *in vivo* studies in diabetic rat models.^256^ These devices featured a bioinorganic membrane composed of albumin, GOx, catalase (CAT), and manganese dioxide (MnO_2_) nanoparticles. This membrane transduced fluctuations in ambient glucose levels into pH changes, which modulated the volume of embedded pH-sensitive hydrogel nanoparticles, thereby dynamically regulating membrane permeability. The membrane was integrated with microfabricated polydimethylsiloxane (PDMS) structures to create compact, self-contained microdevices that operate without external wiring or tubing. A marked increase in the slope of the release curves, corresponding to the rate of insulin release, was observed at 2.5 h when the glucose concentration was elevated from 5 mmol L^−1^ to 20 mmol L^−1^. Across all the *in vitro* experimental cycles, the permeability ratio (*P*_20_/*P*_5_) consistently exceeded 2, indicating robust and reproducible glucose responsiveness. Over a 4 h period, the cumulative insulin release reached 49.7 ± 11.1 µg, equivalent to approximately 5 units per day, well within the therapeutic range for *in vivo* administration in diabetic rat models. In *in vivo* studies, the implanted insulin delivery microdevices effectively maintained normoglycemic blood glucose levels in diabetic rats for up to 7 days post-implantation. In contrast, control animals receiving sham devices exhibited hyperglycemia, with glucose concentrations exceeding 20 mmol L^−1^. In the microdevice-treated group, blood glucose levels declined sharply following implantation, demonstrating a rapid therapeutic onset. A gradual rise in glucose levels was observed after day 10, likely attributable to depletion of the insulin reservoir or progressive loss of insulin bioactivity over time. Collectively, these results underscore the potential of the fabricated ‘smart’ microdevices for rapid, autonomous biosensing and responsive insulin delivery in closed-loop therapeutic systems. Xia *et al.* introduced a novel cell-mediated enzyme delivery strategy employing micrometer-scale, disk-shaped particles referred to as microdevices. These microdevices were fabricated *via* layer-by-layer assembly combined with soft lithography, using catalase as a model therapeutic enzyme.^257^ The enzyme loading was tunable by adjusting the number of catalase layers, enabling precise control over dosage. Catalase retained its catalytic activity within the microdevices and was gradually released over time. Using microdevices embedded with five catalase layers as a representative example, a population of 2.5 × 10^5^ microdevices catalyzed the decomposition of approximately 9.2 × 10^−3^ µmol of hydrogen peroxide per minute. Furthermore, functional cell-microdevice complexes were generated by attaching the catalase-loaded microdevices to the external membranes of K562 cells and mouse embryonic stem cells, demonstrating the feasibility of cell-guided enzyme delivery. Overall, given its modularity and biocompatibility, this technique holds broad applicability across diverse enzymes and cell types, offering a promising platform for therapeutic intervention and prophylaxis in a range of human diseases and injury models.

Obst *et al.* developed a self-regulating drug delivery system featuring a latex diaphragm that functions as a dynamic switch, enabling autonomous insulin release in response to glucose levels.^258^ The device incorporated a pH-sensitive hydrogel matrix embedded with immobilized GOX and catalase, which together catalyzed glucose oxidation and modulated local pH. Enzyme concentrations in the buffer were optimized at 6.9 mg mL^−1^ for GOX and 1.83 mg mL^−1^ for catalase. Upon exposure to glucose, the enzymatic reactions led to a measurable drop in pH, with the glucose-containing solution reaching a final pH of 4.76. The study further investigated the swelling behaviour of the hydrogel under varying glucose concentrations. Hydrogels exposed to higher glucose levels exhibited significantly greater swelling ratios, attributed to enhanced proton generation and subsequent expansion of the hydrogel. This swelling directly influenced the permeability of the latex diaphragm, thereby regulating insulin release in a glucose-dependent manner. Taken together, these findings provide compelling proof of concept for integrating microfabrication techniques with biocompatible, stimuli-responsive polymers to engineer multifunctional drug delivery systems. Such platforms hold promise for closed-loop therapeutic applications, particularly in the context of diabetes management, where autonomous and tunable insulin release is critical.

Author's critical perspective: enzyme-powered microdevices convincingly demonstrate that chemical cues can be transduced into dose-controlled delivery without electronics, through substrate-driven convection or pH-mechanical gating. Yet the same enzymatic chemistry that enables autonomy, particularly glucose oxidase, also introduces biochemical liabilities (H_2_O_2_ generation, acidification) that can erode safety and long-term device stability. Among the strategies surveyed, cell-mediated enzyme delivery is attractive where site-finding is the primary barrier, but its regulatory and *in vivo* stability challenges make it better suited to pathway modulation than precise milligram-level dosing. Across platforms, rigorous deconvolution of active transport *vs.* passive leak, standardized ROS/pH safety metrics, and dynamic performance benchmarking under physiologic oscillations are essential to transform elegant bench physics into clinically reliable therapy. Besides, glucose-responsive diaphragm systems presently offer the clearest control logic from analyte to actuation and appear closest to translation, provided pH excursions are buffered, and cycle-life is proven. In a nutshell, enzyme-responsive microdevices offer a smart and adaptable platform for real-time diagnostic and therapeutic uses. These systems enable precise control of material behaviour, drug release, and signal transduction in response to biologically relevant stimuli by harnessing the catalytic selectivity and stimulus-generating capacity of enzymes. Their versatile design makes them easy to include with different kinds of cells, hydrogel matrices, and microfluidic designs. The studies highlight their promising applications in closed-loop therapeutics, biosensing, and regenerative medicine, among others.

### 7.4. Redox-responsive microdevices

Redox-responsive systems have attracted considerable interest in nanomedicine for their ability to harness intracellular redox gradients, particularly elevated glutathione levels, to enable targeted drug delivery, diagnostics, and theranostic applications. However, despite progress at the nanoscale, translation of redox-triggered mechanisms to microdevice platforms remains limited. However, few studies have explored redox-induced actuation, matrix degradation, or release kinetics at the microscale, underscoring a significant opportunity to advance redox-responsive microdevices as next-generation precision delivery systems.

Chen *et al.* reported a 3D-printed multiwell device compatible with standard lab instrumentation (*e.g.*, plate readers, microscopes), enabling advanced redox-based spectral and electrochemical analyses.^259^ In biomanufacturing, mediated electrochemical probing distinguished intact monoclonal antibodies from fragmented variants, with signal metrics correlating to offline chemical assays. In materials characterization, *operando* spectroelectrochemistry revealed redox-state switching in catechol-based hydrogel films; oxidation enhanced absorbance, while reduction boosted fluorescence. Finally, a synthetic biofilm of redox-responsive *E. coli* was electro-assembled, demonstrating gene induction under both reductive (*via* H_2_O_2_ generation) and oxidative (*via* phenolic signaling) conditions. Collectively, these examples showcased how 3D printing enables bespoke electrochemical platforms for probing redox phenomena across biological and technological domains. A novel strategy for interfacing electronics with biological systems in microdevices using an “electrobiofabrication” toolbox was reported by Shang *et al.*^260^ This approach leverages electrode-induced signals to assemble biopolymer films spatially and electrochemically activate them for signal transduction. Unlike conventional surface modification methods, signal-guided assembly enables on-demand electrode functionalization, streamlining microfluidic sensor design. A chitosan film, selectively localized and covalently modified with redox-active phenolic species, served as a “molectronic” interface, transducing molecular events into electronic signals. These molectronic sensors enabled real-time electrochemical analysis of biomolecules, from small molecules and enzymes to cell-based cytotoxicity assays. To assess cytotoxicity *via* LDH activity, mammalian Caco-2 cells were exposed to Triton X-100. Cell viability was highest at the lowest Triton X-100 concentrations, while increasing concentrations led to pronounced cell shrinkage and lysis, as observed in bright field and green fluorescence images. Notably, treatment with 0.02% Triton X-100 resulted in reduced cell counts due to detachment from the substrate. Quantitative analysis using both a molectronic sensor and a commercial LDH-based cytotoxicity kit confirmed these trends. These results demonstrated the utility of molectronic sensing for real-time cytotoxicity assessment alongside conventional biochemical assays. Overall, this strategy provides a straightforward yet powerful approach to integrating biological sensing within microfluidic platforms, thereby expanding our toolkit for studying redox biology in various contexts.

**Fig. 13:**
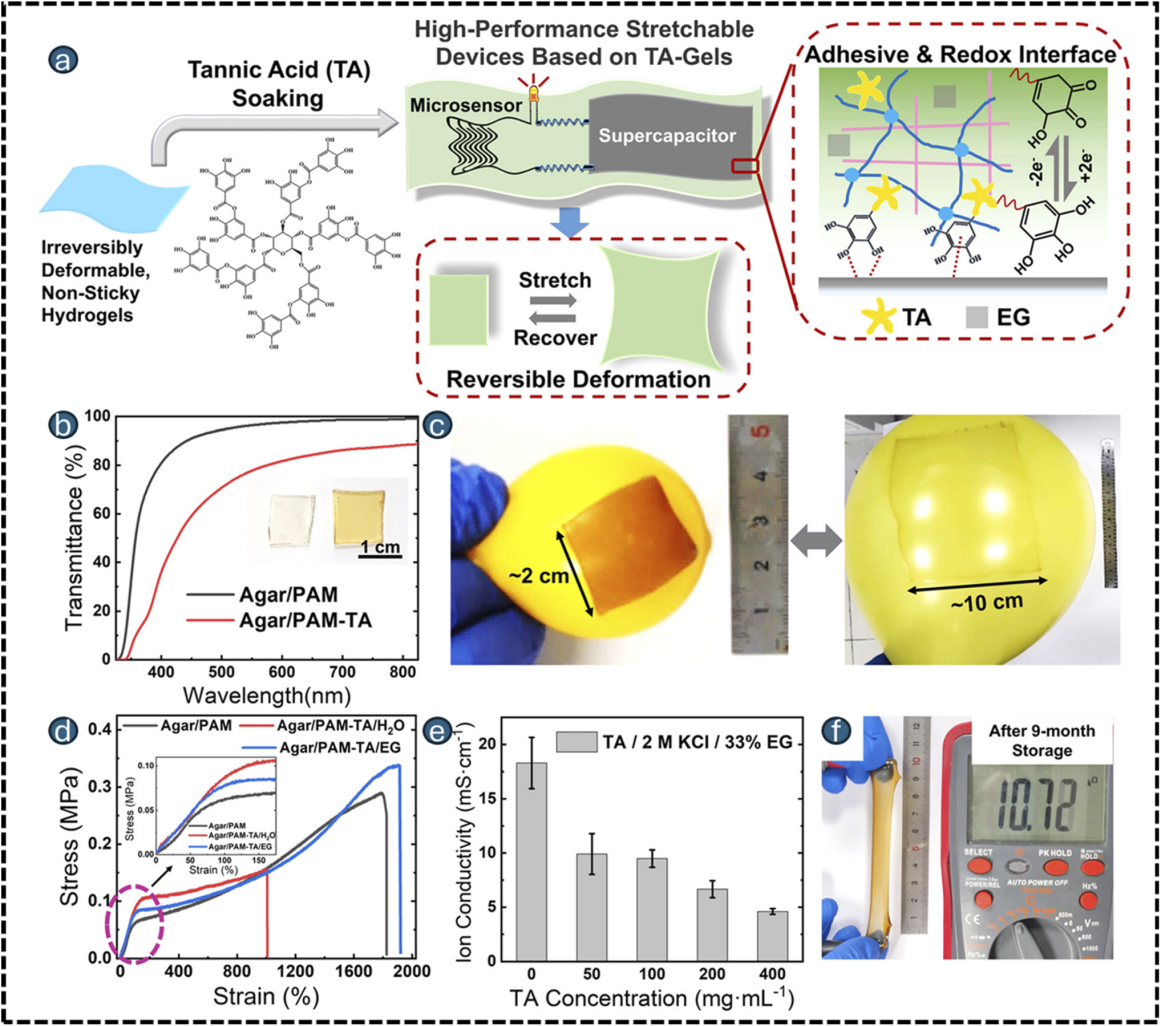
Schematic of stretchable electronic device fabrication, supercapacitors and microsensors—using TA-soaked gel electrolytes that enable reversible stretchability, strong adhesion, and redox-active interfaces. EG is incorporated to enhance ambient stability (a). UV-vis spectra of Agar/PAM before and after TA soaking, with inset images showing visual changes (b). TA-modified hydrogel undergoing biaxial expansion from ∼2 × 2 cm^2^ to ∼10 × 10 cm^2^ (c). Stress–strain curves of Agar/PAM, Agar/PAM-TA/H_2_O, and Agar/PAM-TA/EG; soaking conditions: 200 mg per mL TA, 2 M KCl, and 33% EG for 24 h (d). Ion conductivity of organohydrogels with varying TA concentrations; Agar/PAM-EG (no TA) soaked for 4 h showed minimal swelling (e). Agar/PAM-TA/EG retains >200% stretchability and stable ion conductivity after 9 months of storage. Agar/PAM-TA/EG retains >200% stretchability and stable ion conductivity after 9 months of storage (f). Reproduced from ref. 261. Copyright 2022, American Chemical Society.

According to the findings reported by Wang *et al.*, multifunctional soft electronic devices with reversible stretchability and enhanced electrode/electrolyte interfaces were enabled by polyacrylamide-based double-network organohydrogel electrolytes enriched with tannic acid (TA).^261^ A imparts redox activity and multiple noncovalent interactions, yielding shape-recoverable devices that withstand up to 500% strain (Fig. 13). The excellent shape resilience likely arises from rapid network reformation and polymer chain retraction, facilitated by dynamic binding between mobile polyphenol molecules and the polymer matrix upon stress release. Soaking-treated Agar/PAM gels, including Agar/PAM-TA/H_2_O and Agar/PAM-TA/EG, retained the excellent stretchability and toughness of pristine double-network hydrogels. These mechanical enhancements stem from TA-induced hydrogen bonding, forming dual-crosslinked networks. The TA-rich gels exhibited strong gel-electrode adhesion and high electrochemical capacitance (>200 mF cm^−2^), with a 4-fold enhancement upon co-incorporation of ethylene glycol. Beyond adhesion, TA's polyphenols introduced redox-active interfaces that improved charge storage. Cyclic voltammetry revealed characteristic redox peaks (0.35–0.45 V) and increased current beyond 0.6 V, attributed to reversible quinone/hydroquinone transitions. Galvanostatic charge–discharge curves showed battery-like plateaus, confirming faradaic contributions. Notably, Agar/PAM-TA/33% EG exhibited the highest current and specific capacitance, ∼1.7× and ∼3.8× greater than Agar/PAM and Agar/PAM/EG, respectively. Capacitance further increased with prolonged gel-electrode contact, underscoring the synergistic role of TA and EG in enhancing interfacial redox activity and energy storage. A soft electronic system integrating stretchable supercapacitors and gel-based microsensors maintained stable performance over 1000 deformation cycles at 200% strain, without residual strain or delamination. Overall, these results underscore the multifunctionality of TA-incorporated organohydrogels in soft electronics, offering a promising route to robust, biocompatible, and stretchable devices.

In a separate study by Wang *et al.*, carboxymethyl cellulose (CMC) chains were functionalized with self-complementary nucleic acid tethers and electron donor–acceptor groups to form hydrogels *via* dual crosslinking: duplex nucleic acids and donor–acceptor complexes.^262^ Rheometry analysis revealed that the dopamine/bipyridinium complex-bridged hydrogel exhibited a storage modulus (*G*′) of ∼39 Pa and a loss modulus (*G*″) of ∼12 Pa. In comparison, the redox-responsive hydrogel crosslinked by donor–acceptor units and duplex nucleic acids showed higher stiffness, with *G*′ ≈ 63 Pa and *G*″ ≈ 7.3 Pa. Upon oxidation of dopamine units using sodium persulfate, the hydrogel's stiffness decreased significantly (*G*′ ≈ 29 Pa, *G*″ ≈ 5 Pa; curve *b*/*b*′), consistent with the disruption of donor–acceptor bridges and retention of only nucleic acid duplex crosslinks. Additionally, CMC hydrogels incorporating both donor–acceptor bridges and K^+^-stabilized G-quadruplexes exhibited dually triggered stiffness control. Hydrogel assembly was achieved by mixing two polymer chains: P_E_ and P_F_. P_E_, based on a CMC backbone, was functionalized with dopamine electron donor units and amine-modified G-rich quadruplex subunits. P_F_ carried bipyridinium electron acceptor units and identical G-quadruplex motifs. Upon mixing in the presence of K^+^ ions, a stiff hydrogel (state I) formed through dual crosslinking, *via* donor–acceptor complexes and K^+^-stabilized G-quadruplexes. Disruption of either motif, *via* redox or crown-ether stimulation, yields low-stiffness states. In a nutshell, these dynamic crosslinking mechanisms serve as molecular “memories,” enabling programmable shape-memory and triggered self-healing functionalities. Mage *et al.* recently introduced an aptamer-based voltametric biosensor that enabled the first closed-loop control of doxorubicin levels in live rabbits.^263^ The system integrates a biosensor, controller, and infusion pump to process real-time signals from electrochemically tagged aptamer probes. These probes undergo reversible conformational changes upon drug binding, modulating redox current between the tag and electrode. Embedded within a microfluidic device, the biosensor continuously samples blood directly from the animal's circulation, allowing rapid, quantitative, and specific monitoring of *in vivo* drug concentrations. During controlled infusion, the system rapidly adjusted to set-point changes, achieving 95% of target drug levels within 7.5 ± 2.9 min and maintaining concentrations within 20% of the set point throughout, mirroring durations typical of clinical infusions. BSA-adjusted dosing of DOX in rabbits revealed significant pharmacokinetic variability, with only one animal maintaining target plasma levels for over 80% of the infusion period, despite identical dosing regimens. Real-time biosensor data highlighted the limitations of body surface area normalization in achieving consistent drug exposure. Co-administration of cisplatin significantly altered DOX's pharmacokinetics, increasing plasma levels and reducing time-in-range to 34%, despite identical BSA-adjusted dosing. In contrast, the closed-loop system automatically compensated for this drug–drug interaction, maintaining DOX within the target concentration for 97% of the infusion period, demonstrating robust adaptability to physiological perturbations. Overall, while the current setup operates *ex vivo* and requires catheterization, its design is amenable to miniaturization and future implantation. Importantly, the platform is adaptable for detecting a wide range of biomolecules, provided suitable aptamers or enzyme isoforms are available. Notably, for drugs with complex distribution and metabolism, the system could be adapted to predict concentrations in target organs using advanced PK models, like strategies used in artificial pancreas systems. Moving from an *ex vivo* system to implantable biosensors and infusion devices could provide ongoing dose regulation in everyday settings and support extended disease management.

Author's critical perspective: collectively, these studies advocate for a broader exploration of redox-responsive design principles at the microscale, where modularity, spatial control, and biological relevance converge to unlock new possibilities in diagnostics, therapeutics, and bioelectronic interfacing. Phenolic chemistries and nucleic-acid-inspired motifs provide reversible networks that are both mechanically adaptive and electrochemically addressable, while aptamer-based control shows that closed-loop dosing can correct real-world pharmacokinetic variability. The central challenge is durability: the same redox processes that amplify signals can erode interfaces and mechanics over time, and biorecognition elements may drift *in vivo*. Accordingly, the most translatable paths pair chemically stabilized sensing surfaces with zwitterionic passivation, usage of dual-network mechanics to maintain switching without sacrificing stiffness and adoption of control frameworks that enforce safety constraints alongside performance. Standardizing cycle-life and drift metrics will be key to converting impressive benchtop demonstrations into reliable, long-wear clinical systems.

### 7.5. Hypoxia-responsive microdevices

Hypoxia, prevalent in solid tumors, ischemic tissues, and chronic wounds, offers a distinct physiological trigger for targeted therapy. Hypoxia-responsive microdevices leverage this oxygen deficiency to enable controlled drug release, enhanced tissue penetration, and selective activation of therapeutics. Using chemistries such as azobenzene linkers, nitroimidazole groups, or hypoxia-sensitive promoters, these systems dynamically adjust release profiles in response to fluctuating oxygen levels. Advances in microfabrication and bioMEMS have further enabled multifunctional platforms, including microneedles and implantable reservoirs, capable of delivering drugs, imaging agents, or gene therapies with spatiotemporal precision in hypoxic microenvironments.

Ando *et al.* developed a microdevice platform that replicates intratumoral oxygen gradients, key drivers of the spatially heterogeneous hypoxic microenvironments characteristic of solid tumors. The system mimics a “tumor section” by embedding a cellular layer between two diffusion barriers, enabling the formation of physiologically relevant oxygen gradients through a combination of metabolic activity and physical constraints.^264^ Oxygen distribution within the device was validated *via* numerical simulations and imaging-based oxygen sensor measurements. To assess functional hypoxia, the authors demonstrated spatially resolved hypoxic signaling in cancer cells using immunostaining, gene expression profiling, and targeted drug response assays. To characterize oxygen gradients within the microdevice, a fluorescence-based oxygen sensor was embedded, composed of silica microparticles loaded with Ru (Ph_2_phen_3_) Cl_2_ (oxygen-sensitive) and Nile blue chloride (oxygen-insensitive control). These particles were dispersed in PDMS and layered onto the oxygen barrier pillar. MCF-7 breast cancer cells were micropatterned on collagen I-coated coverslips in circular islands to mimic tumor morphology and assembled into the device. After 24 h of culture, fluorescence imaging revealed a radial increase in Ru (Ph_2_phen_3_) Cl_2_ signal, indicating localized hypoxia. Devices without cells showed uniform, dim fluorescence, consistent with oxygen quenching. Immunofluorescent analysis was performed using pimonidazole to verify cellular response to oxygen gradients within the microdevice. Pimonidazole formed detectable adducts in hypoxic cells, and this was accompanied by elevated staining. Quantitative profiling revealed a plateau in signal intensity within a ∼600 µm radius, tapering off toward the edge (up to 1300 µm). These spatial transitions corresponded to oxygen concentrations of 0.028 and 0.08 mol m^−3^, which is consistent with COMSOL simulations. To evaluate the response of cancer cells to hypoxia-targeted therapy and demonstrate the utility of the microdevice for drug screening, viability assays were conducted using tirapazamine (TPZ). Live-dead staining revealed TPZ-induced cytotoxicity in both normoxic and hypoxic regions, with pronounced MCF-7 cell death under severe hypoxia (≤0.03 mol per m^3^ oxygen, per COMSOL simulation). Notably, untreated cells in deep hypoxia also showed reduced viability. TPZ significantly reduced viability only in the hypoxia microdevice, with no statistical difference observed in the other conditions. Overall, this platform offered precise control over oxygen gradients without the need for complex microfluidic setups. It supported modular integration of additional tumor components and is fully compatible with high-content imaging and high-throughput screening workflows, making it a versatile tool for studying tumor hypoxia and therapeutic responses *in vitro*. Ilana Berger Fridman*et al.* developed a multi-layer microfluidic platform that integrates high-throughput generation of 3D breast tumor spheroids with a linear gradient of five distinct oxygen levels, enabling simultaneous testing of multiple microenvironmental conditions and hundreds of replicates on a single chip.^56^ Using this system, the influence of oxygen gradients on ROS production and the cytotoxic responses to DOX and tirapazamine were evaluated. Oxygen concentrations in the array layer were measured using tris(2,2′-bipyridyl) dichlororuthenium(ii) hexahydrate (Ru-complex), confirming a linear gradient across channels: 19.35 (20%), 15.64 (15%), 9.78 (10%), 4.25 (5%), and 0.34 (0%) oxygen. Intracellular hypoxia sensing in MCF7 spheroids showed low, stable fluorescence under 20%, 15%, and 10% oxygen, but significantly increased at 5% (3.39 ± 0.79 RFU) and ∼1% oxygen (8.58 ± 1.84 RFU), indicating heightened sensitivity to severe hypoxia. Similarly, MCF7 + M2 macrophage co-spheroids exhibited minimal fluorescence under 20%, 15%, and 10% oxygen, but a dramatic rise under 5% (20.27 ± 8.92 RFU) and ∼1% oxygen (23.59 ± 7.47 RFU), confirming robust hypoxia responsiveness in both cell types. In conclusion, the fabricated system enabled simultaneous conditioning and analysis of spheroids across a physiologically relevant oxygen spectrum. DOX showed reduced cytotoxicity under hypoxia, consistent with prior findings, while tirapazamine exhibited increased efficacy at lower oxygen levels, closer to clinical outcomes than standard *in vitro* assays. This system offered a robust, *in vivo*-like model for studying oxygen-regulated tumor responses, accelerating drug screening and mechanistic studies in 3D cancer models. Another study by Schad *et al.* developed a microfluidic platform featuring 3D, endothelial-lined microchannels within an oxygen-tunable environment to address the need for physiologically relevant models of sickle cell disease (SCD).^57^ This system enabled on-chip simulation of hypoxia/reoxygenation (H/R), red blood cell (RBC) sickling, and vaso-occlusion. The cultured endothelium exhibited hallmark microvascular features, including 3D lumen architecture and expression of functional endothelial markers. Using this platform, occlusion assays were conducted to assess the impact of hypoxic preconditioning on RBC-endothelial interactions. Both cyclic and sustained mild hypoxia significantly reduced vaso-occlusion rates to 8.89% and 11.78%, respectively, compared to 57.93% and 55.05% in normoxic controls. RNA sequencing revealed differential gene regulation associated with this protective effect, notably in CYBB, RELN, and SERPINA1. These findings provide mechanistic insight into endothelial adaptation during H/R and highlight potential molecular targets for therapeutic intervention in SCD. A microfluidic platform that integrates precise spatiotemporal oxygen control with long-term optical monitoring was fabricated to investigate tumor spheroid responses to hypoxia. This system recreates physiologically relevant low and cycling oxygen levels, conditions unattainable in conventional culture models, while enabling real-time visualization of cellular dynamics. Using this platform, tumor spheroids underwent reversible swelling and shrinkage in response to oxygen cycling between 0% and 10%, driven by volumetric changes in individual cells. Further, the system was used to assess drug responses under varying oxygen conditions, revealing enhanced DOX uptake under cycling hypoxia compared to chronic hypoxia or normoxia. Spheroids exposed to cycling oxygen profiles showed faster and greater doxorubicin accumulation than those under normoxia (20%), with deeper drug penetration. Initially, uptake was higher in 20% O_2_ spheroids than in 0% O_2_, but after 24–36 h, spheroids under 0% O_2_ surpassed in uptake at all depths, consistent with overall drug accumulation trends. Two-photon microscopy enabled single-cell resolution imaging, uncovering intratumoral heterogeneity in drug distribution. Overall, by combining 3D culture, dynamic oxygen modulation, and high-resolution imaging, this platform offered a powerful tool for dissecting microenvironmental influences on cancer progression and therapeutic resistance, paving the way for more predictive models in oncology research and treatment design. A protocol by Lewis *et al.* outlined the use of developed gelatin-based, oxygen-controllable hydrogels to recreate hypoxic microenvironments *in vitro*.^265^ The hydrogel network was formed *via* a laccase-mediated crosslinking reaction, where laccase catalyzes the formation of diferulic acid (diFA), consuming oxygen in the process. Cells, including cancer and endothelial types, or tissue grafts, can be encapsulated during gel formation, enabling analysis of cellular responses to 3D hypoxic gradients and investigation of underlying mechanisms.

The study by Ravi *et al.* introduced a sophisticated strategy for engineering a 3D bioprintable hypoxia-mimicking supramolecular hydrogel (HMSG), formed *via* the photo-crosslinking of Dex-loaded PEGDA guest polymers with acryloyl β-cyclodextrin (AβCD) host monomers and cobalt nanowires (Co NWs).^266^ The resulting multivalent host–guest nanoclusters endow the hydrogel with excellent mechanical integrity and printability. HMSG enabled sustained delivery of Dex and Co^2+^, creating a supportive microenvironment for encapsulated umbilical cord-derived mesenchymal stem cells (UMSCs). HMSG hydrogels showed a higher Dex encapsulation efficiency (DEE: 96 ± 72) compared to controls (85 ± 35), with reduced burst release. While metabolic activity was similar on day 7, HMSG-treated UMSCs exhibited significantly higher activity on days 14 and 21. Live/dead staining confirmed good viability in both groups, but HMSG supported greater cell proliferation and uniform 3D distribution over time, indicating enhanced biocompatibility and sustained cellular growth. qRT-PCR analysis revealed that HMSG significantly enhanced hypoxia-mediated chondrogenic gene expression (HIF-1α, COL-2, ACAN, SOX-9, COMP, GAG) while suppressing hypertrophic markers (RUNX2, MMP13). Notably, HIF-1α, COL-2, ACAN, and SOX-9 levels peaked by day 21 in the HMSG group, whereas RUNX2 and MMP13 were markedly reduced compared to controls. These results indicate that HMSG promotes UMSC chondrocyte differentiation by modulating gene expression toward a regenerative, non-hypertrophic phenotype. This promoted matrix synthesis, suppresses catabolic signaling, and enhances M2 macrophage polarization, contributing to immunomodulation and attenuation of osteoarthritis severity. A single-layer microfluidic device was engineered to generate controlled oxygen gradients for investigating oxygen-dependent cytotoxicity of anticancer drugs.^267^ Oxygen levels within the cell culture chamber were modulated *via* a spatially confined oxygen-scavenging reaction in an adjacent microchannel. To simulate the *in vivo* oxygen microenvironment of tumor cells, NaOH and pyrogallol were used in adjacent reaction channels to generate oxygen gradients *via* chemical scavenging. Oxygen levels in the cell culture chamber were quantified, resulting in a gradient ranging from ∼2.3% to 11% oxygen, ideal for *in vitro* studies. A groove-patterned PDMS membrane enhanced bonding to the base layer, preventing leakage while enabling efficient gas exchange between channels. To demonstrate functionality, A549 lung carcinoma cells were cultured and exposed to tirapazamine (TPZ) and cisplatin under varying oxygen conditions. Live/dead staining revealed strong cell adhesion and proliferation under both normoxic (21% O_2_) and hypoxic (1% O_2_) conditions. Importantly, no significant differences in viability were observed across the gradient during a 12 h treatment, indicating that moderate hypoxia (0.5–3% O_2_) does not induce apoptosis but instead activates survival pathways in tumor cells. TPZ induced greater apoptosis under hypoxia, confirming its oxygen-sensitive cytotoxicity and potential for targeting hypoxic tumor regions. In contrast, cisplatin was more effective under normoxic conditions, reflecting reduced efficacy in hypoxia due to cellular resistance. These findings highlight the importance of incorporating oxygen tension as a variable in drug screening. The device offered excellent cell compatibility and precise gradient control, making it a valuable platform for evaluating therapeutic responses in physiologically relevant oxygen environments across biomedical applications.

Author's critical perspective: the above-discussed case studies underscore the transformative potential of hypoxia-driven microdevices in advancing drug delivery and biomedical research. Across platforms, oxygen emerges not merely as an environmental parameter but as a first-class control variable that rewires drug transport, cellular metabolism, and vascular function. Chips that deliver programmed O_2_ dynamics uncover phenotypes, such as enhanced doxorubicin penetration under cycling hypoxia, that static assays miss, while hypoxic preconditioning of endothelium reveals context-dependent protection in vaso-occlusion. Matrix-embedded approaches (laccase-gelatin, HMSG) supply self-contained hypoxic niches ideal for differentiation and printing, and hypoxia-inspired differentiation markedly improves BBB fidelity. As hypoxia continues to emerge as a central regulator in cancer, vascular disease, and regenerative medicine, these microdevices pave the way for more physiologically faithful models and targeted therapeutic strategies, bridging the gap between bench and bedside with unprecedented precision. However, the translational path forward is to standardize oxygen-dose waveforms, mechanistically decouple transporter regulation from mass transport, and derisk hypoxia mimics (*e.g.*, Co^2+^) through safer substitutes and tight release control. Platforms that combine temporal O_2_ control, 3D imaging, and disease-relevant mechanics will provide the most predictive, actionable readouts for therapy design.

### 7.6. Ionic-responsive microdevices

Ionic-responsive microdevices are emerging soft electronic platforms that harness ion transport within materials such as ionogels, hydrogels, and ionic liquids to achieve adaptive, stimuli-responsive behavior. Operating through ion migration rather than electronic charge flow, these “ionotronic” systems enable reversible modulation of conductivity, shape, and optical properties. Inspired by biological sensory mechanisms, they support applications in wearable electronics, diagnostics, soft robotics, and human–machine interfaces. Recent advances, including organohydrogels, dual-dynamic networks, and ultrathin ionogels, offer stretchability, self-healing, and strong interfacial adhesion.^268–270^ Despite these innovations, ionic-responsive microdevices remain underexplored, highlighting significant opportunities for foundational research and future technological development.

Yang *et al.* presented the synthesis and comprehensive characterization of zwitterionic poly(3-(1-(4-vinylbenzyl)-1*H*-imidazol-3-ium-3-yl)propane-1-sulfonate) (polyVBIPS) polymer brushes, developed as intelligent, salt-responsive surface coatings.^271^ These brushes were systematically evaluated for their capacity to reversibly modulate surface properties, including wettability, friction, and antifouling behaviour, in response to variations in salt concentration and ionic composition. The polyVBIPS brushes demonstrated pronounced “anti-polyelectrolyte” behaviour, transitioning between hydrophilic states in pure water and hydrophobic states in saturated NaCl solutions. Upon salt addition, the brushes exhibited enhanced hydration, significantly reduced friction, and markedly improved protein resistance, all of which were reversibly tunable through ionic condition adjustments. Under specific salt environments, the brushes effectively resisted protein adsorption and achieved substantial friction reduction. Notably, superlow fouling was attained, with protein adsorption levels falling below 0.3 ng cm^−2^. Frictional performance, assessed *via* colloidal probe atomic force microscopy (AFM), revealed a high-friction regime in water (*µ* ≫ 10^−3^) and a superlow friction regime in salt solutions (*µ* ≈ 1 × 10^−3^). Contact angle measurements and AFM data further confirmed that surface hydration was significantly elevated in saline conditions compared to pure water. The reversible switching of antifouling and frictional properties across multiple cycles underscores the robustness and repeatability of the polyVBIPS brush system, highlighting its potential for advanced smart surface applications. Taken together, salt-responsive zwitterionic polyVBIPS brushes present a promising platform for controllable, “smart” surface functionalization, switching between antifouling and fouling states and between low/high friction as a function of environmental ionic strength. Key quantitative features include <0.3 ng per cm^2^ protein adsorption and friction coefficients as low as 1 × 10^−3^ in proper salt conditions, creating opportunities for advanced applications in biomedical devices, marine coatings, and micro/nanoengineering. In the same domain, poly(vinyl alcohol) (PVA) hydrogels functionalized with zwitterionic polymer brushes, PMEDSAH and PMPC, were developed to enhance boundary lubrication and enable ion-responsive frictional tuning, emulating the tribological behaviour of articular cartilage.^272^ Both PMEDSAH and PMPC-functionalized hydrogels exhibited markedly reduced water contact angles (WCA), ranging from approximately 20° to 30°, compared to higher values observed for unmodified PVA, indicating enhanced surface hydrophilicity. AFM analyses confirmed increased microscale surface roughness post-functionalization, which facilitated the retention of hydration layers, thereby improving lubrication performance. Notably, brush functionalization significantly reduced friction under physiological ionic conditions (0.15 M NaCl), achieving coefficients of friction (*µ* ≈ 0.03–0.06) comparable to natural cartilage. In a nutshell, functionalizing PVA hydrogels with zwitterionic brushes (PMEDSAH, PMPC) yields cytocompatible surfaces with enhanced hydrophilicity and ion-responsive friction, achieving *µ* as low as 0.03–0.06 in saline. These properties closely mimic natural cartilage, supporting their potential as artificial cartilage or lubricious biomedical coatings.

Zhang *et al.* fabricated a bioinspired indwelling MN system for diabetic ulcer treatment, combining template replication and 3D transfer printing to fabricate hydrogel needle tips from ion-responsive PVA encapsulating mesenchymal stem cell (MSC) exosomes.^60^ The MNs feature a detachable medical tape base and dynamically tunable mechanical properties. Sulfate ions enhanced tip stiffness for effective skin penetration, while nitrate ions softened the tips post-insertion, promoting tissue adaptation and sustained exosome release. MSC-exosomes activated fibroblasts, endothelial cells, and macrophages, facilitating re-epithelialization, angiogenesis, and inflammation modulation. *In vivo* studies showed ∼90% wound closure by day 14 (*vs.* ∼60% in controls), with enhanced collagen deposition and >2× angiogenesis. Frictional modulus tuning enabled painless insertion and biocompatibility. Exosome release persisted >7 days, with 2–3× higher local concentrations than non-MN methods. MNs were well-tolerated, showing no adverse effects, highlighting their promise for regenerative wound therapy. In a nutshell, bioinspired indwelling MNs loaded with stem cell-derived exosomes offer tunable mechanics and controlled release for diabetic ulcer treatment by achieving wound closure, enhancing angiogenesis and collagen deposition, and maintaining high local exosome bioactivity. Their ion-responsive design supports effective, well-tolerated regenerative therapy. In a different study by Wan *et al.*, a dual stimuli-responsive wet-spun microfiber hydrogel was developed by co-extruding hot alginate (ionic-responsive) and agar (temperature-responsive) into a precooled, metal ion-rich coagulation bath.^273^ The fibre's microstructure, including anisotropic shrinkage and grooved surface patterns, was tunable *via* extrusion rate and cooling conditions. Incorporation of divalent cations (*e.g.*, Ca^2+^, Zn^2+^) enhanced mechanical strength through double-network formation and intensified blue fluorescence *via* metal–polymer complexation. Addition of metal cations significantly increased tensile strength; typical double-network enhancement was up to 2–5×, with notable anisotropic shrinkage (typically 10–50%). The crosslinking of alginates with Ca^2+^ and Zn^2+^ ions enabled instant hydrogel setting and tunable mechanical properties and hydration. The resulting dual-network structure supported pH-responsive shape memory and sustained antibacterial activity, independent of network functionality. Metal ion integration enhanced stiffness for insertion and contributed to >90% bacterial inhibition, making the hydrogel suitable for adaptive biomedical and biosensor applications. Altogether, by integrating a simple fabrication strategy with multifunctional responsiveness, these dual stimuli-responsive hydrogel fibres offer promising avenues for the development of advanced biomedical systems. The work by Ma *et al.* highlighted the fabrication of a triple-responsive zwitterionic hydrogel synthesized from equal parts l-glutamic acid and l-lysine polypeptides, crosslinked *via* EDC chemistry.^274^ Designed for enhanced biocompatibility and reduced non-specific interactions, the hydrogel responded to ionic strength, pH, and enzymatic activity, enabling precise, site-specific drug release. The hydrogel matrix effectively encapsulated and released both doxorubicin hydrochloride (DOX·HCl, cationic anti-cancer) and diclofenac sodium (anionic anti-inflammatory), demonstrating versatility in dual-drug delivery. In trypsin-rich environments, characteristic of inflamed or cancerous tissues, both drugs exhibited near-complete release within hours, highlighting the hydrogel's enzymatic responsiveness and site-specific therapeutic potential. Elevated ionic strength (*e.g.*, NaCl) further enhanced hydrogel swelling, increasing the swelling ratio by 20–40%, which accelerated drug diffusion. Importantly, the zwitterionic nature of the hydrogel minimized non-specific protein adsorption and cell attachment, improving biocompatibility and selectivity (typically <0.3 ng per cm^2^ protein adsorption). The hydrogel's enzymatic degradability ensured its clearance post-delivery, reducing long-term toxicity risks. In conclusion, this fabricated triple-responsive zwitterionic hydrogel system is positioned as a promising candidate for advanced chemotherapy and anti-inflammatory therapies with reduced side effects, by offering a versatile platform for site-specific and controlled drug delivery.

Datta *et al.* and their colleagues studied the influence of choline-geranate ionic liquids (CAGE-ILs) and choline-based ILs on the permeation of peptides, cyclosporine A (CsA) and vancomycin hydrochloride (VH), respectively. The engineered gels, CsA-CAGE and CsA-CAGE-P, were designed to overcome the skin barrier and enable both localized and systemic drug delivery.^275^ In control experiments, free CsA showed no detectable permeation across porcine skin after 48 h. Pretreatment with oleic acid and palmitic acid improved CsA permeation to 244 ± 4 µg cm^−2^ and 1236 ± 17 µg cm^−2^, respectively. Notably, the CsA-CAGE gel enhanced drug flux by 110-fold compared to the control, while the Pluronic-based CsA-CAGE-P gel achieved a 135-fold increase, underscoring the formulation's significant potential in improving transdermal delivery of challenging therapeutic peptides. The study further demonstrated the therapeutic potential of the CsA-CAGE-P organogel formulation through pharmacokinetic and pharmacodynamic evaluations. Topical application of the gel resulted in a 2.6-fold increase in peak plasma concentration (*C*_max_) and a 1.9-fold enhancement in drug exposure (AUC_0−*t*_) compared to the control, indicating improved systemic absorption. In an imiquimod-induced rat model of plaque psoriasis, topical application of the CsA-CAGE-P gel significantly reduced PASI scores, particularly erythema and scaling. Key physiological markers, skin thickness, blood flow rate, and transepidermal water loss, were normalized, confirming the gel's therapeutic efficacy and potential as a non-invasive treatment option. In the later study, by the same group, the use of choline-based ionic liquids (ILs), particularly choline geranate (CAGE), enhanced the transdermal delivery of VH.^276^ Formulations included choline bicarbonate (CB) combined with oleic acid (CO), palmitic acid (CP), and geranic acid (CAGE). CAGE showed reduced irritation potential, making it suitable for skin application. With a molecular weight of 1449 Da and high hydrophilicity, VH alone showed no detectable skin penetration after 48 h. However, CO and CP formulations significantly enhanced VH permeation across tape-stripped skin, achieving 6729 ± 437 µg cm^−2^ of drug penetration and 3892 ± 215 µg g^−1^ retention within the skin tissue. The fatty acid components likely contributed to lipid disruption and improved drug diffusion. This ionogel formulation demonstrated superior performance. It showed 369 ± 41 µg cm^−2^ and 7543 ± 585 µg cm^−2^ permeation across the intact and tape-stripped skin, indicating its strong potential for both localized and systemic delivery. The combination of ionic liquid and polymeric gel base likely facilitated deeper penetration and sustained release. Overall, the studies by the group highlighted the effectiveness of choline-based ILs, particularly CAGE-P, in overcoming the skin barrier for large-molecule drug delivery.

Author's critical perspective: across these studies, ions emerge as a unifying control knob for biointerfaces, governing hydration and fouling, friction, mechanical state, release kinetics, and barrier permeability. The response should be mapped beyond NaCl to Ca^2+^/Mg^2+^/HCO_3_^−^ and physiologic pH 6.5–7.4, because divalent ions can bridge or collapse zwitterionic chains. Chronic ulcers are characterized by highly variable ionomic conditions, including fluctuations in exudate volume, pH, and electrolyte composition, such that ion-programmed systems may experience failure of the intended stiffening-to-soften mechanical transition. To mitigate this risk, micro-reservoirs may be incorporated to locally supply conditioning ions, or ion-insensitive fallback mechanical designs may be implemented to maintain atraumatic insertion and adequate tissue conformity. Preservation of therapeutic potency should be ensured by verifying exosome or nucleic-cargo RNA/protein integrity and functional bioactivity following repeated ionic–mechanical cycling, while needle-to-needle dose variability should be quantified under GMP-aligned manufacturing conditions. For fiber- or scaffold-based components, anisotropic shrinkage should be exploited for actuation only after shrinkage profiles have been pre-programmed and their dependence on temperature and ionic environment has been rigorously qualified to maintain geometric fidelity. On the biochemical trigger front, over-reliance on trypsin should be avoided because matrix metalloproteinases (MMPs), neutrophil elastase, and cathepsins predominate in diseased human tissues; cleavage motifs should be replaced or augmented with pathophysiologically relevant substrates. Moreover, as ionic strength and pH fluctuations co-occur with proteolysis *in vivo*, it is recommended that response-surface modeling be applied across these combined stimuli to predict release kinetics, define safe operating windows, and prevent unintended burst, and that such modeling be implemented accordingly in device optimization. Among non-invasive strategies, CAGE ionogels are standout enablers for large-molecule transdermal; however, they have translation hinges on chronic safety and exposure tunability. To bridge elegant benchtop performance to clinical reliability, future work should standardize ionic operating envelopes, align tribological testing with physiologic lubricants, deconvolute multi-trigger release under realistic pathophysiology, and establish long-cycle durability/biocompatibility post-sterilization. Platforms that pair ion-aware design with mechanics-and biology co-tuning will be best positioned for translation.

## 8. From trigger to tolerance: biosafety profiling of smart microdevices

### 8.1. Biocompatibility and immunogenicity of stimuli-responsive microdevices

**Fig. 14:**
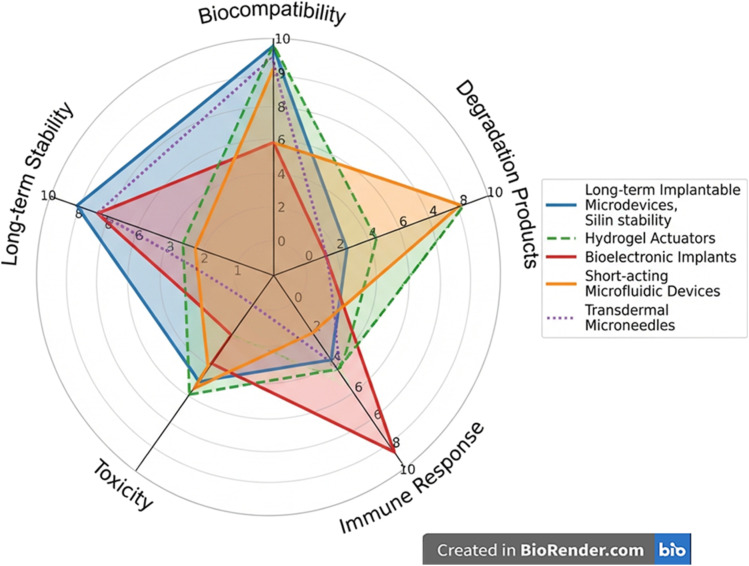
Radar plot illustrating comparative biosafety profiles of representative implantable, wearable, hydrogel actuators, microfluidic and transdermal microdevices. This comparative visualization supports device-specific biosafety analysis and emphasizes how interaction mode and duration shape risk profiles. This image has been created as a Creative Common using the BioRender software (https://www.biorender.com/).

Biocompatibility and immunogenicity are the cornerstone of biomedical engineering and a critical determinant of the safety and efficacy of stimuli-responsive microdevices. These devices, designed to interact with biological systems and respond to specific internal or external stimuli, must do so without eliciting harmful effects. Biocompatibility encompasses the ability of a material or device to perform its intended function in a living system without causing toxicity, inflammation, allergic reactions, or immune rejection. For microdevices, biocompatibility extends beyond inertness to include functional integration, minimal immune activation, and long-term stability within dynamic biological environments. ISO 10993-1:2018 provides a framework for biological evaluation, including cytotoxicity, sensitization, genotoxicity, and implantation studies.^277^ Stimuli-responsive microdevices are increasingly used in applications such as targeted drug delivery, biosensing, tissue regeneration, and minimally invasive diagnostics. Their biocompatibility is influenced by multiple factors, including material composition, surface properties, degradation behaviour, and the nature of the stimulus they respond to.^278^ Testing for biocompatibility involves a combination of *in vitro* and *in vivo* assessments. Cell viability assays, cytokine profiling, and histological evaluations help determine whether the device causes cellular damage, inflammation, or tissue disruption.^279^ For blood-contacting devices, hemocompatibility tests are essential to ensure they do not induce clotting or hemolysis.^280^ Long-term implantation studies further reveal how the body adapts to or rejects the device over time (Fig. 14).^281,282^

Biocompatibility modulates uptake indirectly by influencing protein adsorption, membrane interactions, and immune signaling.^283^ Avoiding toxicity begins with the careful selection of materials. Natural polymers like chitosan, alginate, and hyaluronic acid are favoured for their inherent compatibility with biological tissues, while synthetic polymers such as polyethylene glycol (PEG) and poly (lactic-*co*-glycolic acid) (PLGA) are engineered to minimize adverse reactions.^284,285^ Beyond hydrogels, natural silk emerges as a compelling material for biomedical microdevices, prized for its outstanding mechanical resilience and intrinsic biocompatibility. Silk-based constructs exhibit a remarkable ability to undergo dynamic mechanical transformation. Upon hydration, these devices shift from a rigid, brittle configuration to a soft, pliable state, significantly reducing mechanical stress at the tissue interface. This transition occurs rapidly, with complete softening, which facilitates smooth implantation while avoiding prolonged rigidity. Such tunable mechanics not only ease insertion but also mitigate tissue trauma, enhancing long-term biocompatibility.^286^ The surface chemistry of these devices plays a pivotal role; hydrophilic coatings and neutral charges tend to reduce protein adsorption and cellular activation, thereby lowering the risk of inflammation or immune system engagement. Additionally, the size and shape of the microdevice influence its interaction with cells; smaller particles may be internalized more readily, which can be beneficial for targeted delivery but also raises concerns about intracellular toxicity or unintended immune activation.^287^ The internalization of microdevices by cells is a critical determinant of their therapeutic efficacy and safety. Uptake mechanisms are governed by a complex interplay between device size, surface properties, and biocompatibility. These factors influence not only the route of entry but also the intracellular fate and potential for cytotoxicity or immune activation. Clathrin-mediated endocytosis or caveola-mediated uptake and micropinocytosis are the dominant uptake mechanisms for the microdevices with sizes <200 nm and ranging from 200–1000 nm, respectively. While phagocytosis favours the size >1 µm.^288^

Immunogenicity, the potential to trigger an immune response, is another critical aspect. Devices that mimic biological structures or are cloaked with biocompatible coatings can evade immune detection, whereas those with foreign or reactive surfaces may be flagged by the body's defence mechanisms.^289^ This can lead to acute inflammation, chronic immune activation, or even fibrous encapsulation, which compromises device functionality. To mitigate these risks, researchers often employ stealth strategies such as PEGylation or the use of zwitterionic materials that resist protein binding and immune cell recognition. PEG creates a hydrophilic, sterically repulsive barrier that inhibits opsonization and macrophage recruitment, thereby reducing clearance and inflammatory signaling. However, repeated exposure may lead to anti-PEG antibody formation, necessitating alternative approaches. Zwitterionic materials, such as phosphorylcholine, sulfobetaine, and carboxybetaine polymers, offer a promising substitute. These materials mimic the charge neutrality of cell membranes and exhibit ultra-low fouling characteristics, resisting nonspecific protein binding and complement activation.^290,291^ In summary, ensuring biocompatibility in stimuli-responsive microdevices is a multifaceted challenge that requires thoughtful design, rigorous testing, and a deep understanding of biological interactions. By avoiding toxic and immunogenic responses, these devices can safely integrate into the human body, unlocking their full potential in precision medicine and advanced healthcare solutions (Table 4).

**Table 4:** Comparative illustration of biosafety profiles by microdevice class

Device class (examples)	Primary host interaction mode	Predominant biosafety risks (device-specific)	Mechanistic drivers/context	Representative mitigation strategies	Ref.
Long-term implantable microdevices (drug depots, pumps, hydrogel actuators, neural/bioelectronic implants)	Chronic, subcutaneous or intracavitary/organ contact (weeks–years)	Foreign body response (FBR) → macrophage activation, FBGCs, fibrotic encapsulation; chronic low-grade inflammation; mechanical/electrical isolation; degradation/leachables and accumulation toxicity (*e.g.*, acidic oligomers from hydrolysable polymers); device failure	Mechanical and chemical mismatch *vs.* soft tissue; stiffness-sensing by myofibroblasts; surface protein adsorption; long-term hydrolysis/oxidation of polymers; miniaturized electrodes provoking glial scarring in neural tissue	Surface softening/soft overcoats to reduce stiffness cues; adhesive interfaces to eliminate suture-induced tension anisotropy; immunomodulatory coatings; geometry/material tuning (thin, compliant, low-modulus); controlled-degradation chemistries; peri-implant anti-fibrotic	292–294
Hydrogel actuators (implantable, stimuli-responsive)	Chronic soft-tissue contact; cyclic actuation (mechanical/electro/thermal)	As above (FBR, fibrosis) + material fatigue/fragmentation under cyclic actuation; degradation by-products causing local acidosis; swelling-pressure tissue injury	Hydrolysis of polyester segments; oxidative chain scission; repeated strain altering surface chemistry and particulate; sustained leachables	Use biostable or well-characterized degradable networks; neutral-by-product chemistries; mechanically matched moduli; lubrication/low-friction skins; *in situ* imaging of degradation to tune lifetime	295–297
Bioelectronic implants	Chronic neural or peripheral nerve contact; electrical stimulation/recording	FBR with fibrotic cuffing; glial scarring; signal drift/impedance rise; haemorrhage-related inflammation at insertion; encapsulation → loss of mass transport/electrical coupling	Penetrating microelectrodes damage microvasculature; high local stiffness; micromotion relative to tissue; chronic immune activation	Softening substrates, ultra-thin/mesh probes; adhesive nerve interfaces to avoid fibrosis; pharmacologic or biomaterial immunomodulation; atraumatic insertion methods	294 and 298
Short-acting microfluidic devices (catheter-coupled chips, extracorporeal or transiently blood/tissue-contacting microfluid)	Transient contact with blood or interstitial fluids; minutes to hours to a few days	Hemocompatibility risks: platelet activation, thrombosis, complement activation, hemolysis at high shear; material compatibility with biofluids; biofouling	Shear gradients in stenotic-like microchannels drive vWF-mediated platelet adhesion; geometry/topography and surface charge; stagnant zones → coagulation; high shear → SIPA/hemolysis	Hemodynamically optimized channel design; anti-thrombogenic coatings; surface smoothing/charge control; controlled shear exposure; anticoagulation protocols validated in flow assays	299–301
Transdermal MNs (solid/coated/dissolving/)	Minimally invasive, short-duration skin penetration (minutes–hours; episodic use)	Localized skin events: transient erythema, edema, irritation; mild pain/pruritus; rare infection (if asepsis fails); short-lived immune activation (intended for vaccines) with low systemic toxicity	Microchannel formation across stratum corneum; material residues (poorly dissolved shafts); operator/application variables; adjuvanted formulations in skin; however, minimally safe	Sterile manufacturing; fast-dissolving, biocompatible matrices; patch wear-time control; skin prep/after-care; adjuvant dose control	302–305

### 8.2. Stimulus-responsive microdevices and the tissue toll

Stimulus-responsive microdevices represent a transformative leap in biomedical engineering, offering precise, on-demand drug delivery tailored to physiological or external cues. These devices are designed to respond to stimuli such as pH, temperature, enzymes, magnetic fields, or light, enabling controlled therapeutic release at targeted sites. However, their interaction with biological tissues, often termed the “tissue toll”, remains a critical challenge, particularly in long-term implantable applications. While implantable microdevices hold immense potential for targeted and minimally invasive drug delivery, their long-term success is often compromised by the body's natural defence mechanisms. One of the most significant challenges is the foreign body response (FBR), a complex cascade of immune reactions triggered by the presence of a non-native object in biological tissue.^306^

When a biomedical microdevice is implanted into the body, the immune system perceives it as a foreign entity and initiates a defensive response aimed at isolating the device from surrounding tissues. One of the most prominent outcomes of this response is fibrotic encapsulation, the formation of a dense, collagen-rich fibrous tissue layer around the implant.^307^ This process is primarily driven by the activation of immune cells, notably macrophages and fibroblasts, which release signaling molecules that promote collagen deposition and extracellular matrix remodeling. The resulting fibrous capsule acts as a semi-permeable barrier, significantly impeding the diffusion of therapeutic agents from the device into adjacent tissues. Consequently, the bioavailability of the drug at the target site is reduced, and the drug release profile becomes unpredictable. Instead of a controlled and consistent release, the drug may be delivered more slowly, unevenly, or in diminished quantities, compromising the intended pharmacological effect. This inefficiency is particularly critical in therapies requiring precise dosing, such as chemotherapy, hormone replacement, or insulin delivery.^308^ In severe cases, the altered performance of the device may necessitate recalibration or even surgical removal and replacement, thereby increasing patient risk and healthcare costs.

Next, when a biomedical microdevice is implanted into the body, it inevitably disrupts the local tissue microenvironment and is promptly identified as a foreign entity. This recognition initiates a complex cascade of immune responses aimed at neutralizing or isolating the perceived threat. If this response is not effectively resolved, it can progress into chronic inflammation, a sustained and maladaptive immune state that poses significant risks to both device functionality and surrounding tissue health.^309^ The initial immune response is characterized by the rapid recruitment of macrophages, which attempt to phagocytose the device or wall it off from the surrounding tissue. However, when the device remains in place and lacks sufficient biocompatibility, macrophages become chronically activated. These cells begin to secrete pro-inflammatory cytokines such as TNF-α, IL-1β, and IL-6, which amplify the immune response and attract additional immune cells, including neutrophils, fibroblasts, and lymphocytes.^306^ Over time, this persistent inflammatory signaling leads to fibroblast proliferation and excessive ECM deposition, particularly of collagen, culminating in fibrosis. Fibrosis replaces normal tissue architecture with dense, fibrous connective tissue, resulting in: (a) mechanical displacement or compression of the device; (b) altered chemical and mechanical interface between the device and host tissue; (c) impaired drug diffusion; and (d) reduced sensor accuracy.^306,308^ This remodeled tissue environment can severely compromise the intended therapeutic or diagnostic function of the device. For drug delivery systems, fibrotic encapsulation may hinder drug release, leading to subtherapeutic dosing or unpredictable pharmacokinetics.^310^ For sensing or stimulation devices, tissue stiffening and scarring can dampen signal transmission, reduce sensitivity, and even cause mechanical failure. In extreme cases, the device may need to be surgically removed or replaced, increasing patient risk and contributing to higher healthcare costs. Thus, mitigating the foreign body response is critical to ensuring the long-term success and safety of implantable biomedical microdevices.

A notable influence of stimulus-responsive microdevices on the tissue toll includes biofouling, an undesirable accumulation of biological materials, such as proteins, cells, and ECM components, on the surface of implanted devices.^311^ This phenomenon begins almost immediately after implantation, as the device encounters bodily fluids and tissues. Within seconds to minutes, plasma proteins like albumin, fibrinogen, and immunoglobulins adsorb onto the device surface. This protein layer alters the surface chemistry and can trigger immune cell adhesion. Macrophages, neutrophils, and fibroblasts are recruited to the site and adhere to the protein-coated surface. These cells may attempt to degrade or isolate the device, contributing to inflammation and fibrosis. Over time, cells secrete ECM proteins, including collagen and fibronectin, forming a dense biofilm or fibrotic capsule.^312,313^ Biofouling can clog microchannels or release ports, impeding drug diffusion. Accumulated biological material can interfere with moving parts, degrade conductive surfaces, and increase device impedance. This affects devices like microvalves, pumps, and electrodes. This leads to inconsistent dosing, delayed therapeutic effects, or complete delivery failure.^314^

Taken together, stimulus-responsive microdevices offer precise, minimally invasive therapeutic solutions by adapting drug release or sensing to real-time physiological cues. However, their long-term performance is often compromised by FBR, a cascade of immune reactions that leads to chronic inflammation, fibrotic encapsulation, and biofouling. These tissue-level changes can impair drug diffusion, distort pharmacokinetics, and degrade sensor accuracy, ultimately threatening device efficacy and longevity. To overcome these challenges, future designs must integrate biocompatible materials, anti-fouling surfaces, and immune-modulating strategies. A synergistic approach that combines smart responsiveness with tissue integration is essential for the sustained clinical success of implantable microdevices.

### 8.3. Accumulation-induced toxicity in stimuli-driven microdevices

In the rapidly evolving landscape of biomedical engineering, stimuli-driven microdevices have emerged as transformative tools in diagnostics, targeted drug delivery, and minimally invasive therapies. Their ability to navigate complex biological environments and deliver payloads with spatial and temporal accuracy has positioned them at the forefront of personalized medicine and nanotherapeutics. However, alongside their immense potential lies a growing concern: accumulation-induced toxicity. Despite the growing interest in stimuli-responsive microdevices for targeted drug delivery, there remains a notable scarcity of comprehensive toxicity studies in the existing literature. Most available data focus primarily on their design, responsiveness, and therapeutic efficacy, while systematic evaluations of their biocompatibility, long-term safety, and potential off-target effects are limited. This gap underscores the need for more rigorous preclinical and clinical investigations to ensure the safe translation into biomedical applications. In this context, understanding the toxicity is critical for advancing the safe and effective use in biomedical applications. Despite significant progress in developing diverse material platforms, including natural and synthetic polymers, inorganic nanomaterials, and organic nanomaterials, comparative analyses of their distinct toxicity mechanisms remain limited. Natural polymers often exhibit superior biocompatibility, yet their degradation products can trigger immune responses, whereas synthetic polymers may introduce concerns related to non-biodegradability or accumulation. Similarly, inorganic nanomaterials differ fundamentally from organic nanomaterials in terms of surface chemistry, biodistribution, and reactivity, leading to divergent toxicity pathways. Current discussions surrounding material design approaches to mitigate immunogenicity frequently remain broad, without detailing how specific physicochemical attributes influence biological interactions. Parameters such as degradation rate, surface charge, particle size, and chemical composition critically determine cellular uptake, inflammatory signaling, and long-term tissue responses (Table 5). However, the lack of systematic evaluation of these properties obscures the underlying mechanisms governing toxicity and hinders the formulation of rational design strategies. A clearer understanding of how material characteristics shape toxicity pathways is therefore essential to guide the selection, optimization, and development of microdevices with improved safety profiles for clinical translation.

**Table 5:** Comparative summary of major material classes highlighting their design flexibility, chemical composition, surface charge characteristics, degradation behaviour, toxicity profiles, and immunogenicity^a^

Materials	Material design	Chemical composition	Surface charge	Degradation rate	Toxicity	Immunogenicity	Ref.
Natural polymers	Can be engineered into hydrogels, microgels, films, microneedles, microcapsules, and scaffolds, responsive to pH, enzymes, redox, or ionic changes	Polysaccharides or proteins composed of carbohydrate monomers, including uronic acids, glucose units, or amino acids with reactive functional groups	Typically, neutral to negatively charged due to hydroxyl, carboxyl, or sulfate groups, some may be slightly cationic depending on functionalization	Generally biodegradable *via* enzymatic, hydrolytic, or oxidative pathways, the rate ranges from fast (highly hydrophilic polymers) to slow (protein-based structured polymers)	Generally low toxicity due to their biological origin and biocompatibility, degradation products are typically non-toxic	Mostly low immunogenicity, some variability depending on source, purity, and presence of protein or polysaccharide sub-units	315–321
Synthetic polymers	Highly tunable, can be engineered into hydrogels, micelles, nanogels, microneedles, microcapsules, allowing precise control of responsiveness (pH, redox, temperature)	Composed of covalently linked monomers (esters, amides, vinyl groups, acrylates), functionalizable side chains	Can be neutral, cationic, or anionic depending on the monomer or functional group	Tunable from rapid to very slow depending on polymer architecture (ester content, hydrophobicity, crosslinking)	Generally low to moderate, some cationic polymers may show dose-dependent cytotoxicity	Generally low, may trigger mild responses based on surface charge or residual monomers	322–327
Inorganic nanomaterials	Can be formulated as nanoparticles, porous carriers, microcapsules, or microrobots, responsive to pH, enzymes, magnetic fields, redox conditions, and ionic concentrations	Made of metals (Au, Ag, Pt), metal oxides (TiO_2_, ZnO, Fe_2_O_3_), silica, calcium salts, or layered inorganic structures	Often negative or neutral, the surface can be functionalized to carry positive/targeting groups	Highly variable, some degrade rapidly in acidic or enzymatic conditions, others slowly dissolve or are largely persistent	Ranges from low to moderate, depending on composition, dose, and dissolution products	Generally low, some inorganic particulates may provoke mild inflammatory responses depending on size and surface	328–333
Organic nanomaterials	Used as small-molecule carriers, lipid-based microstructures, organogels, metal organic framework (MOFs)/covalent organic framework (COFs), can be designed for redox, enzyme, or pH-triggered release	Prepared from organic molecules, lipids, aromatic linkers, or covalent frameworks with reactive functional groups	Mostly neutral or slightly negative, tunable through head-group modification	Usually fast to moderate degradation through hydrolysis, oxidation, or enzymatic cleavage	Generally, low, lipid-based and small-molecule systems are well tolerated, and degradation products are typically benign	Very low, organic frameworks and lipophilic carriers rarely elicit immune responses unless impurities remain	334–337

a Natural polymers – hyaluronic acid, chitosan, silk fibroin; synthetic polymers – polydimethylsiloxane (PDMS), poly(methyl methacrylate) (PMMA), poly(*N*-isopropylacrylamide) (PNIPAAm), polyvinyl alcohol (PVA), polyethyleneimine (PEI), polyvinylpyrrolidone (PVP); inorganic nanomaterials – Fe_2_O_3_, ZnO, Zn_2_GeO_4_; organic nanomaterials – metal organic framework (MOFs), covalent organic framework (COFs).

When microdevices are introduced into the body, their long-term fate becomes a critical consideration. Unlike conventional pharmaceuticals, which are typically metabolized and excreted, microdevices, particularly those composed of non-biodegradable or slowly degrading materials, may persist within tissues and organs over extended periods. To this end, important concerns regarding their biocompatibility, potential accumulation, and long-term safety necessitate thorough investigation during the development process. The prolonged presence of microdevices in biological systems may trigger unintended biological responses, including chronic inflammation, oxidative stress, and potential organ dysfunction. These adverse interactions underscore the importance of evaluating long-term biocompatibility and safety during device development. The liver, spleen, kidneys, and even the brain are particularly vulnerable to such accumulation due to their roles in filtration and immune surveillance.^338,339^ Moreover, repeated administration of microdevices, often necessary for chronic conditions, exacerbates the risk of bioaccumulation.^340^ The complexity of biological systems means that even devices designed for targeted delivery can exhibit off-target localization, further increasing the risk of toxicity. Factors such as particle size, surface charge, hydrophobicity, and material composition significantly influence biodistribution and clearance, making the design of these devices a delicate balance between functionality and safety.^341^ Hydrophobic microdevices are more prone to non-specific protein binding, forming a protein corona that flags them for immune removal. They may also aggregate, reducing targeting precision and increasing the risk of vascular blockage. A study on biomaterials used in medical devices found that hydrophobic surfaces promoted biofilm formation by pathogens like *Staphylococcus epidermidis* and *Pseudomonas aeruginosa*, increasing infection risk and device-related toxicity.^342^

The review by Wang *et al.* highlights that stimuli-responsive delivery systems, while enhancing therapeutic efficacy, can accumulate in inflamed tissues due to their responsiveness to pathological stimuli (*e.g.*, acidic pH, overexpressed enzymes). This targeted accumulation, if uncontrolled, may result in off-target effects and organ stress, particularly in the liver and spleen.^343^ A study of multiple microbubble formulations in cardiac tissue exposed to diagnostic ultrasound revealed significant capillary damage, proportional to the mechanical index (MI) used.^344^ No damage was seen with ultrasound or microbubbles alone, but combined exposure caused microvascular haemorrhage and endothelial injury. Myocardial lactate release exhibited a marked increase with rising MI levels. Macroscopic examination revealed areas of intramural haemorrhage, particularly over the beam elevation regions in hearts exposed to both contrast agents and high-MI ultrasound. Light microscopic analysis further confirmed the presence of capillary ruptures, erythrocyte extravasation, and endothelial cell damage. At an MI of 1.6, the mean percentage of ruptured capillaries was observed to be 3.6 ± 1.4%, indicating a significant degree of microvascular injury. In contrast, sulfur hexafluoride microbubbles, used in echocardiographic imaging, demonstrated very good safety profiles with rare adverse reactions (*e.g.*, mild allergic symptoms, transient nausea, self-resolving back pain). No severe toxicity or organ dysfunction was noted in large clinical series.^345^

The cytotoxicity studies by Willis *et al.* primarily examined the delivery of the chemotherapeutic agent etoposide to cancer cell lines using rotating magnetic nanoparticle (MNP) clusters as microdevices, with a specific focus on evaluating cellular viability post-treatment.^346^ Etoposide was magnetically delivered *via* MNP clusters to U138 glioblastoma and H2122 lung adenocarcinoma cells over a 10 cm biomimetic channel. Cell viability, assessed by MTT and trypan blue assays, showed that direct exposure to 100 µM etoposide for 24 h reduced U138 cell viability to ∼23%, indicating strong cytotoxicity. The cytotoxic effect of magnet-propelled etoposide was statistically significant (*p* < 0.05) in comparison to controls, demonstrating that drug efficacy was retained using this delivery approach. In another study by Alon *et al.*, a miniaturized magnetic device, an array of permalloy (Ni_80_Fe_20_) bars sputter-deposited on glass, that generated highly localized, tunable magnetic fields to control neuron-like cell migration and organization at the microscale.^347^ The cytotoxicity studies demonstrated that the iron oxide nanoparticles (MNPs) used to induce magnetic sensitivity in PC12 cells were found to be highly biocompatible under the experimental conditions. After incubation with MNPs, XTT cell viability assays showed that approximately 99% of the PC12 cells remained viable after 5 days, with the results normalized to non-treated control cells. This indicated minimal cytotoxicity associated with the nanoparticle uptake at the studied concentrations and timeframes.

Photothermal-based microdevices for therapeutic applications often utilize photothermal agents that generate localized heating when exposed to a specific wavelength of light, resulting in targeted cell destruction.^348^ Cytotoxicity studies for these devices generally focus on evaluating the safety and efficacy of the photothermal agents and the induced heat on cells. The cytotoxicity studies by Lee *et al.* focused on evaluating the safety and photothermal therapy (PTT) effects of a reduced graphene oxide-branched polyethyleneimine–polyethylene glycol (rGO-BPEI–PEG) nanocomposite on 3D brain tumor spheroids generated by the microfluidic device.^349^ The cytotoxicity of rGO-BPEI–PEG nanocomposites was evaluated in U87MG brain tumor cells treated with varying concentrations (20–60 µg mL^−1^) for 4 h, followed by 808 nm NIR laser irradiation (1 W cm^−2^). Without irradiation, cell viability remained ∼90%, confirming good biocompatibility. Post-irradiation, viability dropped below 60% at 60 µg mL^−1^, indicating effective photothermal killing of cancer cells. Confocal microscopy confirmed cellular uptake in both single cells and spheroids, essential for efficient photothermal therapy. In a nutshell, rGO-BPEI–PEG nanocomposites in the photothermal microdevice showed low inherent toxicity but effectively induced tumor spheroid cell death upon NIR laser exposure, demonstrating their safety and efficacy as PTT agents in 3D tumor models generated *via* droplet-based microfluidics. In a separate finding by Song *et al.*, the cytotoxicity studies demonstrated the biocompatibility and therapeutic efficacy of the Cu-doped polydopamine (Cu-PDA) nanoparticles delivered by MNs.^350^ Cu-PDA NPs delivered *via* dissolving MNs enabled effective PTT under NIR laser irradiation, rapidly raising local temperature to ∼50 °C and inducing tumor cell apoptosis. Without NIR exposure, the NPs showed minimal toxicity, confirming skin compatibility. *In vivo* studies on B16 melanoma-bearing mice demonstrated significant tumor suppression with minimal systemic toxicity, driven primarily by NIR-triggered photothermal effects. Taken together, Cu-doped polydopamine NPs delivered *via* dissolving MNs are biocompatible and safe without irradiation, but under NIR light, they enable synergistic photothermal and chemodynamic therapy for minimally invasive and effective treatment of skin melanoma with low side effects. The cytotoxicity studies evaluated by Ge *et al.* highlighted the biocompatibility and safety of the theranostic separable double-layer microneedle (DLMN) patch designed for diabetes management.^350^ Cytotoxicity evaluation using human skin fibroblasts (HSF) showed that MN patches, comprising a GelMA base and PCM arrowheads loaded with melanin NPs and metformin, were biocompatible and safe. After 24 h incubation, CCK-8 assays indicated high cell viability, with no significant toxicity. Cell proliferation assays on days 4 and 7 confirmed sustained cell growth, supporting the long-term safety of the microneedle components. Lee *et al.* developed a photo-crosslinkable GelMA-based microfluidic co-culture device to study photothermal therapy and cancer cell migration. Using 10% w/v GelMA hydrogels as semi-permeable barriers, MCF7 breast carcinoma and U87MG glioblastoma cells were cultured.^351^ Core–shell microcapsules were fabricated in a microfluidic device, featuring ethyl cellulose shells encapsulating gold nanorod-loaded pNIPAAm NPs for NIR laser-based photothermal therapy applications. The device demonstrated the photothermal effects of gold nanorods on cancer cells. Gold nanorod cytotoxicity was concentration-dependent; optimal doses enabled effective photothermal tumor cell ablation without harming co-cultured cells. NIR laser irradiation reduced cancer cell viability to below 10%, regardless of cell type, confirming the strong photothermal efficacy of the treatment. Cancer cell migration was studied in a microfluidic co-culture device, revealing that the tumor microenvironment influenced endothelial cell behaviour. Cancer cells attracted endothelial cells to form capillary-like vascular networks and migrated toward collagen gel-embedded microchannels. Collectively, cytotoxicity data confirmed that the hydrogel microfluidic device can offer a biocompatible environment for cancer cell culture, and that gold nanorods can selectively induce cell death upon NIR activation without inherent toxicity at therapeutic doses.

Overall, stimuli-responsive microdevices represent a promising frontier in precision medicine, offering controlled and site-specific therapeutic delivery. However, their potential to induce toxicity remains a critical concern. The dynamic behaviour of these devices, triggered by internal or external stimuli, can lead to unintended biological interactions, including chronic inflammation, oxidative stress, and tissue damage. Current literature on their toxicological profiles is limited, highlighting the urgent need for comprehensive *in vivo* studies and standardized safety assessments. Addressing these gaps is essential to ensure the safe clinical translation of stimuli-responsive microdevices and to fully harness their therapeutic potential.

## 9. Translational regulatory and AI-driven barriers

Smart endogenous-stimuli microdevices are miniaturized implantable, intra-tissue, or luminal systems that release drugs in response to internal biochemical cues such as pH, redox gradients, enzymatic activity, or hypoxia, without requiring external actuation. Many are microfabricated polymeric or hybrid micro/nano constructs and consequently fall under drug–device combination pathways. In the United States, such products are treated as combination products, comprising drug and device constituent parts reviewed together, with regulatory jurisdiction determined by the primary mode of action (PMOA) under 21 CFR Part 3 and overseen by the FDA's Office of Combination Products (OCP). In the European Union, although “combination product” is not a standalone legal category, drug-led integral products are governed *via* MDR Article 117, while device-led products containing ancillary medicinal substances are regulated under MDR Rule 14. In India, most hardware-based platforms are classified as medical devices under the Drugs & Cosmetics Act and the Medical Devices Rules (MDR) 2017, with risk-based classes (A–D) defining dossier requirements and licensing routes.^352–354^ Some endogenous-responsive platforms border on micro/nanorobotics (*e.g.*, catalytic micromotors that respond to local biochemistry) and will likely trigger additional safety and control questions around navigation, retrieval, and biodegradation; recent reviews highlight translational barriers and the need for standardized control and safety taxonomies. Electrically or magnetically influenced micro/nanorobots used for targeted release introduce energy-coupling and software validation concerns akin to SaMD.^355,356^ The succinct list of FDA-approved or authorized microdevices with practical regulatory details are enlisted in Table 6.

**Table 6:** Additional FDA-approved or authorized, stimulus-responsive microdevices (with practical regulatory details)

Platform (sponsor)	Responsiveness	U.S. pathway/classification	Key approval indicators are commonly referenced	Primary source(s)
iLet bionic pancreas (beta bionics)	Closed-loop insulin dosing driven by interstitial glucose (endogenous cue)	Clearance (May 19, 2023) for ACE pump + dosing software components; interoperable AID system assembled from iCGM + ACE + iAGC categories (CDRH)	Time-in-range (70–180 mg dL^−1^), HbA1c change, hypoglycaemia metrics, human-factors/usability	FDA Press Announcement (May 19, 2023)^357^
CamAPS FX (CamDiab)	An interoperable automated glycaemic controller (iAGC) app that adapts insulin delivery in real time from CGM signals	FDA authorization (May 24, 2024) as iAGC; supports pregnancy indication in U.S. labeling	Time-in-range, HbA1c; pregnancy subgroup evidence (AiDAPT RCT)	Healio coverage (May 24, 2024); Drug Delivery Business News (May 24, 2024)^358^
Tandem Control-IQ (tandem diabetes care)	The algorithm automatically adjusts the basal and gives correction boluses based on the CGM trends	*De novo* (2019) (first interoperable automated dosing controller); builds an AID with ACE pump + iCGM	Time-in-range improvement *vs.* standard care; avoidance of hypoglycaemia; ecosystem interoperability	Fierce Biotech MedTech summary of FDA authorization (dec 16, 2019)^359^
NeuroPace RNS® system	Closed-loop, responsive neurostimulation detects epileptiform activity and delivers stimulation on-demand	PMA (P100026; 2013)	Median % reduction in seizure frequency; safety endpoints; chronic implant performance	FDA SSED (P100026); Cleveland Clinic; Epilepsy Foundation (program overviews)^360^
Vagus nerve stimulation (VNS therapy)	Periodic + on-demand stimulation to modulate neural activity; “magnet dose” responsive to symptom onset (patient-triggered)	Legacy approvals (device class III with PMA; adjunctive therapy for drug-resistant focal epilepsy)	Seizure-frequency reduction; safety and tolerability	CURE Epilepsy overview (summarizes FDA-approved neurostimulation devices)^361^
Deep brain stimulation (DBS) for epilepsy	Programmed stimulation of specific targets to reduce seizure frequency (clinician-tuned, periodic; not fully closed-loop)	PMA-regulated neurostimulator systems (device class III)	≥50% responder rate over time; safety in long-term implant	CURE epilepsy overview (FDA-approved devices: VNS, RNS, DBS)^362,363^
FDA-authorized microneedling devices (general and plastic surgery)	Mechanically responsive (not endogenous biochemical), but relevant for transdermal and patch hardware precedent (*e.g.*, skin penetration depth control)	*De novo*/510(k) pathways; product code QAI; limited to aesthetic indications	Mechanical integrity; skin safety endpoints; labeling controls	FDA Microneedling Devices page (product code QAI; *de novo* and 510(k) links)^364^

Chemistry, Manufacturing, and Controls (CMC) requirements scale with the development phase but must consistently establish the identity, purity, strength, quality, and stability of both drug and device components, with defined control of critical design attributes such as microreservoir geometry and stimuli-responsive polymer transitions. Recent FDA training materials highlight phase-appropriate IND expectations, emphasizing early control of raw materials and incorporation of functionality testing for dose-delivery systems in alignment with ICH Q1/Q6A.^365,366^ For combination devices, FDA quality reviewers additionally expect reproducible dose-delivery performance and functionality assessments aligned with CTD structure and ICH stability principles, including for injectors and implants. NIH SEED's combination products guide further clarifies PMOA determination, constituent-part interactions, and lifecycle considerations. A persistent regulatory hurdle is translating device design controls into drug CMC dossiers (and *vice versa*), ensuring that stimuli-triggered release kinetics are articulated as essential drug-delivery outputs supported by appropriate verification and validation plans.^367,368^

Endogenous-responsive microdevices often incorporate nanostructures or release nano-objects upon degradation, requiring a risk-based biocompatibility strategy aligned with ISO 10993-1:2025 and ISO 14971, alongside FDA's 2023 guidance, which outlines expectations for nano/sub-micron components, *in situ*-polymerizing or absorbable materials, and detailed chemical characterization.^369^ ISO/TR 10993-22 further defines nanomaterial-specific requirements, including characterization, toxicokinetics, release behaviour, and risk assessment, highly relevant for devices employing nano-engineered triggers.^370^ Practical analyses emphasize common pitfalls such as agglomeration, protein-corona formation, and unintended internal exposure when nano-objects are released. A key regulatory hurdle remains demonstrating biological equivalence and safety of stimuli-responsive chemistries across heterogeneous disease microenvironments, supported by orthogonal chemical characterization and extractables/leachables assessments, which become more critical as microdevice volumes shrink.^371,372^

Smart microdevices often contain polymers, sensors, or payload-sensitive architectures that cannot tolerate steam sterilization, making radiation (ISO 11137-1:2025) and ethylene oxide (EO; ISO 11135) the primary alternatives, each requiring validated doses, residual-controls, and demonstrated material compatibility.^373,374^ The 2025 revision of ISO 11137-1 introduces higher allowable energy limits, more flexible audit intervals, and simplified dosimetry language, improving feasibility for complex microfabricated configurations.^375^ Comparative overviews of EO *versus* radiation methods highlight critical considerations for delicate devices, including validation burdens and risks of material degradation. A key regulatory hurdle is achieving sterility assurance without compromising stimuli-responsive chemistries (*e.g.*, acid-labile linkers) or altering microchannel geometry, while also controlling EO residuals or radiation-induced polymer changes that may shift release kinetics. For drug-led products employing user-interfaced components (*e.g.*, implant delivery kits, on-body reservoirs), regulators expect human factors validation and functionality testing demonstrating accurate dose delivery; FDA's drug-device quality tips cite ICH Q6A and M4Q to justify inclusion of delivery-system functionality in specifications and stability. The draft FDA EDDO guidance is intended to standardize what performance data are essential across product families.

AI- and ML-driven fabrication of endogenous-responsive microdevices for drug delivery faces several key barriers, including insufficient high-quality training datasets that limit model accuracy for predicting complex microenvironmental responses, as highlighted in reviews on AI-enabled micro/nanorobotics that emphasize challenges in data standardization and real-time closed-loop control integration.^376^ Another major barrier is the difficulty in translating AI-guided optimization of material synthesis and device architecture into reproducible, scalable microfabrication workflows, as noted in analyses of AI-enhanced micro/nanorobots, where practical deployment is hindered by constraints in fabrication precision and environmental sensing reliability.^377^ Furthermore, endogenous-responsive systems require accurate modeling of dynamic biochemical signals, yet current AI/ML frameworks often fail to capture patient-specific heterogeneity in drug release profiles and microenvironmental variability, reflecting broader limitations identified in AI-guided nanomedicine where heterogeneous *in vivo* performance and underutilization of mechanistic data impede smart-device translation.^378,379^ Finally, integrating AI-assisted additive manufacturing methods (*e.g.*, 3D-printed microdevices) remains constrained by unresolved issues in autonomous quality control and prediction of biomaterial behaviour, as indicated in AI-assisted microneedle fabrication studies that stress lingering gaps in AI-driven process reliability and product consistency.^380^ When microdevices include embedded sensors and adaptive algorithms, such as systems that detect endogenous biological signals and automatically adjust dosing, their software components may fall under regulatory categories like Software as a Medical Device (SaMD) or device-embedded software. The FDA's Digital Health Center of Excellence provides guidance on how AI/ML-enabled devices are evaluated, including new lifecycle approaches such as predetermined change control plans that allow regulated updates to learning algorithms. Policy analyses summarize the shift from static to learning systems and the prevalence of AI/ML devices cleared *via* 510(k).^381–383^ India's draft guidance on medical device software clarifies expectations across the software lifecycle under the existing Medical Devices Rules. A key regulatory hurdle is explaining algorithm transparency, dataset representativeness, and change-management for any adaptive control tied to endogenous bio signals, ensuring the software's role in dose decisions remains auditable.

Taken together, from the author's viewpoint, smart microdevices face multiple materials and analytical pitfalls: stimuli-responsive chemistries (pH, redox, and enzymatic triggers, among others) require validation that confirms trigger specificity within diseased-tissue ranges while limiting off-target release in healthy physiology, with regulators closely examining preconditioning and accelerated-ageing steps per FDA's essential drug-delivery output expectations. Micro- and nanostructured components introduce additional complexity, as ISO/TR 10993-22 mandates characterization of nano-object release, toxicokinetics, and degradation products, issues particularly critical for bioresorbable microcapsules and nanoporous membranes. Sterilization can further alter performance: radiation may induce chain scission or cross-linking that shifts swelling behaviour and release kinetics, and although updated ISO 11137-1 parameters offer more flexible validation conditions, they still require robust material-compatibility data to ensure function is preserved. Finally, extractables and leachables remain a prominent risk in small-volume microdevices with high surface-area-to-volume ratios; FDA's ISO 10993 guidance emphasizes detailed chemical characterization and exposure-driven risk assessment to prevent clinically meaningful leachable burdens.

## 10. Challenges and future trends

Biomedical microdevices for regulated drug delivery and theranostics signify the forthcoming evolution of delivery methods that combine miniaturization, cost-effectiveness, batch manufacturability, reproducibility, and integration with very large-scale integration electronics, facilitating programmable and active modulation of drug release. A comprehensive assessment of the technology readiness levels (TRLs) for these systems is presented in Table S1, which summarizes the current developmental status of laboratory-scale innovations reported between 2008 and 2026. The current advancement of drug-delivery microdevices is in its early stages, with most technologies remaining in the proof-of-concept phase. However, several factors contribute to the continued presence of certain stimulus-responsive microdevices in the drug delivery pipeline.

While research has offered several inspirations for the design and development of new materials, the creation of synthetic systems that can respond to stimuli in a controllable and predictable manner poses considerable hurdles. Specific obstacles exist in replicating biological systems that require structural and compositional gradients over multiple length scales for coordinated and systematic responsive actions. To address these issues, numerous stimuli-responsive systems have been developed, primarily focusing on polymeric solutions, gels, surfaces, and interfaces, with a lesser emphasis on polymeric solids. The states of matter impose varying degrees of limitations on the mobility of polymeric segments or chains, facilitating dimensional responsiveness in systems with elevated solvent content and minimal energy inputs. The difficulty in constructing these stimuli-responsive microdevices lies in developing networks that can elicit subtle molecular alterations, which in turn produce substantial physicochemical responses to external or internal stimuli. In a nutshell, the challenges can be summarized as including material constraints, feature size reduction, complexity of patterns, precise control over particle characteristics, and limitations in microfluidic particle fabrication, among others.^384,385^

Moreover, one of the challenges involves engineering stimuli-responsive interfaces with diverse molecular architectures at the interfacial areas that demonstrate responsiveness to stimuli. Each chain of the interface must incorporate stimuli-responsive components to elicit significant responses detectable by both sides of the interfacial anchoring points. Additionally, it is essential to regulate the interfacial chain lengths, molecular weights, and chain stiffness. Despite substantial advancements in the development of precisely regulated polymerization techniques that provide well-defined macromolecular blocks with stimuli-responsive properties, understanding the physicochemical elements of these systems remains a challenge. While stimulus-responsiveness in solutions is generally accessible, the regulation of responsive ranges, the influence of solvent–solute interactions, and the mechanorheological behaviour in response to stimuli remain inadequately understood. These interactions are crucial in micro- and nanofluidics, as well as in other aspects of polymer rheology. Colloidal dispersions at sub-nanometer diameters offer extensive possibilities for developing water-based stimuli-responsive systems, wherein colloidal nanoparticles with diverse morphologies, forms, and bioactive properties can be produced.

Stimuli-responsive surfaces and interfaces pose significant problems, necessitating coordinated synthetic and design efforts to manage surface and interfacial density, control the chain length of anchoring macromolecules, and ensure their flexibility. The advancement of polymeric surfaces and interfaces that exhibit exact selectivity and self-repairing capabilities is of significant interest, and recent research indicates a need for further progress and a deeper understanding of various aspects of stimuli-responsive polymeric surfaces and interfaces. Moreover, improved mechanical integrity is crucial for enhancing traditionally fragile polymeric materials. Achieving a balance between mechanical stability and rapid response times, reversibility, and processing conditions will be vital for numerous novel applications, particularly in biomedical systems. A deeper understanding of the inclusive modifications in copolymers derived from natural building components, including saccharides and peptides, will open new pathways for regenerative medicine and tissue engineering, particularly in relation to cell adhesion and differentiation. Addressing the challenges of biocompatibility, biodegradability, and non-toxicity is crucial across all physical states. The advancement of various stimuli-responsive macromolecules that offer diverse mutual impregnability responses will be vital for future applications. The reversibility and rapidity of stimuli-responsiveness in these states, particularly within solid networks, are crucial. The design of appropriate chemical structures to regulate metastable equilibrium energy states will establish the conditions necessary for the creation of coordinated heterogeneous networks.

### 10.1. Clinical challenges

From a clinical perspective, there must be a distinct and recognized unmet clinical need where existing remedies remain inadequate. Clinical implementation of endogenous stimuli-responsive microdevices faces multifaceted challenges rooted in the inherent complexity and variability of human physiology. One of the foremost difficulties is achieving reliable *in vivo* biocompatibility, as endogenous biochemical cues, such as pH, enzymatic activity, and redox gradients, vary widely across organs, disease states, and even within microenvironments of the same tissue, resulting in inconsistent device activation and unpredictable therapeutic release.^386,387^ Studies of biopolymer-based and nanocomposite carriers show that endogenous stimuli often lack the uniformity and stability necessary for consistent trigger-dependent behaviour, which complicates their translation from controlled laboratory conditions to heterogeneous clinical settings.^388,389^ Patient-to-patient variability further exacerbates these issues, making it difficult to calibrate activation thresholds that reliably distinguish pathological signals from physiological noise without risking suboptimal release or inadvertent overdose.^390^ Long-term implantation adds additional challenges, including chronic inflammation, unpredictable degradation kinetics, and the potential toxicity of breakdown products, factors that persist despite advances in polymeric microdevice architectures aimed at improving durability and tissue integration.^391,392^ Case studies in related stimuli-responsive systems highlight these translational obstacles: for instance, pH-responsive nanospheres exhibit effective release profiles under controlled conditions but demonstrate inconsistent behaviour in *in vivo* tumor environments due to spatial and temporal heterogeneity of acidity.^393^ Likewise, endogenous-responsive nanocarriers developed for myocardial infarction therapy show therapeutic promise but require meticulous optimization to accommodate rapidly shifting biochemical and enzymatic landscapes in the injured myocardium.^394^ Collectively, these findings underscore that while endogenous stimuli-responsive microdevices carry significant therapeutic potential, their clinical translation depends on a deeper mechanistic understanding of physiological variability, rigorous long-term biocompatibility evaluation, and comprehensive *in vivo* validation to ensure consistent and safe performance across diverse patient populations. In this context, no endogenously-based microdevices have progressed to any phase of clinical evaluation. The literature (https://clinicaltrials.gov/) includes only a few mentions of microdevice technologies overall, underscoring their scarcity (Table 7).

**Table 7:** Summary of ongoing clinical trials evaluating microdevice platforms across various interventions

Clinical trial identifier	Microdevice type	Drug(s)	Intervention/treatment	Clinical phases and study description	Study start (actual) and study completion (estimated)	Ref.
aNCT04135807	Implantable device	FDA-approved drugs used systemically for the treatment of gliomas	Primary brain tumors	Early Phase I: this study evaluates drugs delivered through a needle-sized microdevice that is inserted into the tumor at the start of surgery and removed at its conclusion. The goal is to determine whether such microdevices can identify which agents demonstrate the most effective therapeutic response in malignant brain tumors	Study start – 2020-03-03	395
					Study completion – 2028-06-01	
bNCT07193862	Implantable device	FDA-approved combination drug products	*In situ* evaluation of drug response in patients with colorectal liver metastasis	Early Phase I: this prospective phase 1 safety study evaluates percutaneous implantation and surgical retrieval of a microdevice in patients with colorectal cancer liver metastases. The microdevice is inserted 3–5 days before the scheduled hepatic resection and removed en bloc with the tumor. Patients are monitored for 14 days postoperatively to ensure that device placement and retrieval do not increase complication rates. Feasibility will be assessed by analyzing tissue adjacent to the device to evaluate drug diffusion and the localized therapeutic effects of chemotherapy with or without immune-modulating agents	Study start – 2025-10-15	396
					Study completion – 2026-04-15	
cNCT04045470	Implantable device	19 FDA-approved anti-cancer drugs	Cutaneous T cell lymphoma and peripheral T cell lymphoma	Phase I: this study evaluates the safety of inserting and removing a microdevice loaded with 19 drugs into a cancerous skin lesion. The microdevice is designed to screen multiple approved and investigational agents directly within a patient's tumor to identify the most effective therapeutic options	Study start – 2019-12-11	396
					Study completion – 2027-01-01	
dNCT04199026	Implantable device	10 FDA-approved anti-tumor drugs	Metastatic or recurrent sarcoma	Phase I: patients undergo percutaneous implantation of up to three drug-delivery microdevices up to two days before standard-of-care surgery. The microdevices release multiple drugs in the absence of unacceptable toxicity. During surgery (approximately two days later), the implanted microdevices are removed	Study start – 2025-02-25	397
					Study completion – 2028-12-31	
eNCT04399876	Implantable device	20 FDA-approved anti-cancer drugs	*In situ* in prostate cancer	Phase I: this pilot study represents the first evaluation of this investigational approach. It assesses the feasibility of using an MR-guided implantable microdevice to measure localized tumor responses to chemotherapy and other clinically relevant agents in participants with prostate cancer scheduled for radical prostatectomy	Study start – 2020-06-22	398
					Study completion – 2026-03-01	
fCT04701645	Implantable device	—	Ovarian, fallopian tube, and peritoneal cancer	Phase I: participants will undergo percutaneous placement of several microdevices into a selected tumor deposit prior to surgery. The microdevices will remain in the tumor for approximately 24 ± 8 h to allow localized drug–tissue interactions. During the planned, clinically indicated surgical procedure, the tumor mass and the implanted microdevices will be resected	Study start – 2022-11-01	399
					Study completion – 2026-06-30	

a NCT04135807 – FDA-approved drugs (Temozolomide, Lomustine, Irinotecan, Carboplatin, Lapatinib, Osimertinib, Abenaciclib, and Everolimus, among others).

b NCT07193862 – FDA-approved combination drug products (Doxorubicin, FOLFOX, Botensilimab, Balstilimab, Bevacizumab, FOLFIRI, FOLFIRINOX, AGEN2373).

c NCT04045470 – FDA-approved anti-cancer drugs (Romidepsin, vorinostat, bexarotene, brentuximab vedotin, pralatrexate, and mogamulizumab, among others).

d NCT04199026 – FDA-approved drugs (doxorubicin hydrochloride, ifosfamide, vincristine, irinotecan, temozolomide, pazopanib, everolimus, polyethylene glycol, ganitumab, and temsirolimus).

e NCT04399876 – FDA-approved anti-cancer drugs (Abiraterone, Enzalutamide, Pembrolizumab, Ipilimumab, Carboplatin, Docetaxel, and Olaparib, among others as well as combinations).

f CT04701645 – not applicabile.

From the author's viewpoint, addressing the clinical limitations of endogenous stimuli-responsive microdevices requires a multifaceted strategy that integrates material innovation, patient-specific optimization, and improved biological modeling. Enhancing *in vivo* biocompatibility can be achieved through the development of advanced polymeric and hybrid biomaterials engineered to minimize inflammatory responses, reduce degradation-product toxicity, and maintain functional stability across diverse physiological conditions. To counteract variability in endogenous cues such as pH, enzymes, and redox gradients, microdevices can incorporate multimodal or hierarchical sensing mechanisms capable of integrating multiple biomarkers rather than relying on a single fluctuating trigger. This increases activation fidelity even in heterogeneous tissue environments. Patient-to-patient variability may be managed by incorporating adaptive or tunable architectures, such as adjustable thresholding mechanisms, feedback-controlled release modules, or personalized calibration based on individual biochemical profiles collected through pre-treatment diagnostics. Long-term implantation issues can be mitigated by using materials with predictable degradation kinetics, surface modifications that reduce fibrotic encapsulation, and coatings that enhance tissue integration. Improved *in vitro* and *in vivo* modeling systems, such as organ-on-chip platforms and physiologically relevant disease models, can also help predict device performance across complex environments before clinical deployment. Furthermore, implementing iterative preclinical evaluation pipelines, including chronic implantation studies, real-time biosensing assays, and longitudinal biocompatibility testing, can substantially reduce translational uncertainty. Collectively, these strategies provide a roadmap for improving safety, reliability, and clinical robustness in future generations of endogenous stimuli-responsive microdevices.

### 10.2. Industrialization challenges

Industrialization of endogenous stimuli-responsive microdevices is constrained by intertwined manufacturing, quality, and sterilization hurdles that are distinct from bench-scale prototyping. First, scalable nanofabrication must balance resolution *versus* throughput while preserving device geometry and materials performance. Recent reviews of microneedle and microsystem manufacturing highlight persistent trade-offs, limited testing standardization, and reproducibility gaps that complicate mass production and technology transfer. Second, achieving batch-to-batch consistency for stimuli-responsive materials demands GMP/QbD frameworks that define CQAs (*e.g.*, trigger threshold, release rate), embed PAT/real-time controls, and qualify continuous or microfluidic processes; EMA/ICH guidance on QbD and recent analyses of nanomedicine scale-up emphasize these control strategies as essential for industrial robustness. Third, terminal sterilization can alter polymeric matrices and sensing layers (chain scission, oxidation, cross-linking), shifting activation thresholds or payload stability; comparisons of γ, e-beam, and X-ray modalities and FDA materials guidance underscore modality-specific effects and the need for dose windows compatible with functionally sensitive microdevices. Fourth, integration as drug–device combination products introduce additional validation burdens (co-packaged/single-entity controls, design changes, ISO 10993 biocompatibility evidence) that must be addressed within the Office of Combination Products' framework during scale-up. Case studies illustrate both progress and gaps: rapidly manufacturable glucose-responsive microneedle patches show promising closed-loop performance but still face durability, stability, and standardized production challenges, while patents and protocols demonstrate conceptual scalability yet leave industrial validation open. Parallel advances in high-throughput particle manufacturing (*e.g.*, microfluidic and sequential nanoprecipitation approaches) directly target uniformity and scale, offering templates for stimuli-responsive payload fabrication and downstream purification at industrial volumes. Collectively, industrial translation will hinge on design-for-manufacture early in R&D, modality-aware sterilization validation, and QbD-anchored control strategies that link stimulus response to measurable CQAs across scale.

From the author's viewpoint, addressing the industrialization challenges of endogenous stimuli-responsive microdevices requires a coordinated strategy that integrates advances in scalable fabrication, quality-by-design control systems, and sterilization-aware materials engineering. To overcome the resolution–throughput trade-offs inherent in nanofabrication, emerging high-throughput 3D-printing and microfabrication methods, such as digital-light processing, hybrid additive manufacturing, and optimized microneedle architectures, offer promising routes for scalable, reproducible mass production while retaining microscale structural precision, as highlighted in recent analyses of 3D-printed microneedle arrays and microsystem manufacturing. Achieving batch-to-batch consistency can be strengthened by embedding Good Manufacturing Practice (GMP) and Quality-by-Design (QbD) principles early in development; regulatory frameworks from EMA and ICH emphasize defining Critical Quality Attributes (CQAs), real-time analytics, and continuous manufacturing as mechanisms to maintain process uniformity during scale-up, consistent with findings from nanomedicine manufacturing reviews that highlight reproducibility and quality-control challenges at industrial scale. Sterilization-related degradation, a major barrier for polymer-based microdevices, can be mitigated by selecting radiation-resilient polymers, integrating antioxidant stabilizers, and tailoring dose windows through comparative evaluations of γ-irradiation, e-beam, and X-ray modalities, which reveal distinct oxidative and structural impacts on device materials, and therefore guide modality-specific sterilization protocols that preserve device responsiveness. For products that qualify as drug–device combination systems, developers can streamline industrial readiness by aligning fabrication workflows with the FDA's combination-product guidance, which clarifies requirements for coordinated validation, biocompatibility assessment (ISO 10993), and controlled component integration during scale-up. Case studies of glucose-responsive microneedle patches demonstrate how manufacturability can be improved through modular integration of printed sensors, stable fluidic pumps, and scalable materials processing, yet also illustrate the need for durability testing and standardized fabrication pipelines to transition from prototype to industrial reproducibility.^400,401^ Parallel advances in microfluidic-assisted nanoparticle manufacturing and sequential nanoprecipitation further address scaling limitations by enabling continuous, uniform production of stimuli-responsive carriers, offering blueprints for translating controlled lab-scale synthesis into industrial-scale, high-purity manufacturing workflows.^402,403^ Collectively, these strategies, design for manufacturability, QbD-driven process control, sterilization-conscious material selection, and regulatory-aligned integration, provide a pathway to overcome current industrialization barriers and accelerate the translation of endogenous stimuli-responsive microdevices into reliable, scalable medical products.

However, considering the strengths and constraints outlined above, the following recent studies discussed herein provide substantive, forward-looking perspectives on the design and translational potential of microdevices or endogenous-responsive microdevices for biomedical use. In the work by Su *et al.*,^404^ the promise of wearable microbial fuel cells as autonomous power suppliers for electronic skins by using the biofuels in human sweat was discussed, with exceptional long-term robustness, sustainable power delivery, low material and fabrication cost that eliminates the need for purified enzymes, utilization of native skin microbiota and improved system resilience as microbial metabolism adapts to fluctuating sweat composition and environmental conditions. However, the concept is still in its infancy with the probable challenges that include lower power density than enzymatic systems, lack of standardized architecture, start-up time for the microbial colonization and biofilm formation that requires an initial conditioning period, health and safety considerations that require careful control of biocompatibility and hygiene and integration with e-skin electronics, among others. Resolving these limitations is critical to realizing wearable MFCs as dependable, autonomous “living” power generators for the sustained operation of e-skin platforms. Microfluidics enables precise manipulation of microliter-scale fluids within microchannels (1–1000 µm), where strictly laminar flow allows accurate control of molecular gradients and cellular microenvironments. Leveraging these advantages, Ravi *et al.* developed a 3D tumor-on-a-chip platform integrated with single-cell RNA sequencing to dissect tumor–stroma–immune interactions, revealing macrophage polarization as a key regulator of cancer invasion.^405^ Similarly, Lupsa *et al.* employed a microfluidic model to study cancer-associated fibroblast (CAF) interactions with prostate cancer spheroids, demonstrating that CAF-mediated spatial and temporal cues significantly influence therapeutic responses to docetaxel and darolutamide.^406^

Microelectrode arrays (MEAs) engineered for interfacing with the central nervous system enable high-resolution neural recording and targeted electrical stimulation, as exemplified by cochlear implants and deep-brain stimulation systems.^407^ Bennett *et al.* investigated blood–brain barrier (BBB) disruption resulting from stab injuries and implantation of Utah MEAs.^408^ Both injury paradigms induced upregulation of proinflammatory gene expression alongside downregulation of genes associated with tight and adherent junction proteins, collectively indicating compromised BBB integrity. Microcoil arrays exploiting magnetic fields, largely insensitive to biological material properties, have been developed to enhance spatial selectivity.^409^ These parylene-C-coated, four-turn solenoidal microcoils (250 µm turn radius, 31.75 µm wire radius) demonstrated highly localized stimulation, with ∼99.8% power attenuation over a 100 µm separation. Finite-element modeling predicted safe operation at 60 mA and 5 kHz for up to 10 min, while Rattay's activating function confirmed confined neural activation. Each microcoil occupied ∼400 µm per stimulation channel, approximately 84% narrower than conventional cochlear implant electrodes, indicating substantially improved spatial selectivity. However, a key limitation of these materials is the pronounced mismatch in Young's modulus between the MEAs and brain tissue, which is further exacerbated by unfavourable surface chemistry that impairs cell adhesion and proliferation. Poor tissue integration allows MEAs to micro-move within the brain, inducing mechanical strain, exacerbating tissue damage, and ultimately degrading device performance over time. For cochlear implants, limitations include biocompatibility and hermetic packaging, power management and thermal dissipation, spatial selectivity and electrode Interaction, neural coding and latency, among others.^410^

Recent advances in artificial intelligence (AI) and machine learning (ML) have substantially accelerated the design, property prediction, optimization, and high-throughput screening of polymeric systems as well as nanocarriers intended for microdevice fabrication. These data-driven approaches enable more efficient exploration of complex formulation spaces and facilitate rapid identification of material candidates with desired functional attributes. However, despite these developments, the direct application of AI or ML in the fabrication of microdevices, particularly endogenous or biologically integrated microdevices in the field of drug delivery, remains underexplored. Consequently, the present discussion adopts a broader, generalized perspective, acknowledging the emerging potential of AI-guided methodologies while recognizing the current limitations in reported implementations specific to microdevice manufacturing. ML was employed to represent multiscale polymer structures and perform virtual polymer screening, while complementary simulations were integrated with ML workflows to validate the predicted responsive polymer properties.^411^ ML predicted composite behaviour under external stimuli and accelerated novel formulation discovery.^412^ Xu *et al.* employed a Bayesian optimization algorithm to design a series of experimental plans, which were subsequently validated through systematic experimentation.^413^ Through iterative refinement, the optimized parameters yielded a hydrogel exhibiting markedly enhanced overall performance, including improved strain sensitivity and flexibility. Hamel *et al.* employed an ML-based framework to design thermally activated, responsive composite beam structures by addressing the nonlinear mechanical behaviour inherent to such systems.^414^ Their approach integrated an evolutionary algorithm (EA) with finite element (FE) simulations to strategically optimize the placement of simple active components within the beam, enabling targeted shape-morphing when subjected to heating. Similarly, Carrico *et al.* utilized 3D printing in combination with a Bayesian-driven AI methodology to develop soft ionic polymer–metal composite (IPMC) actuators for soft robotics.^415^ By leveraging ionomeric precursor materials, the additive manufacturing process facilitated the fabrication of monolithic 3D IPMC devices with embedded sensing and actuation functionalities.

Taken together, form author's perspectives, in endogenous stimuli-responsive microdevices, AI and ML face significant barriers, including the lack of standardized datasets, challenges in modeling complex biological triggers, limited real-time adaptability, and substantial regulatory and translational gaps. Smart microdevices and microrobots for drug delivery still face translational challenges, particularly in integrating responsive materials with reliable fabrication workflows. Thus, applying AI/ML directly to microdevice fabrication for endogenous responsiveness remains limited. ML models may propose theoretically optimal designs, but achieving regulatory approval requires rigorous, reproducible clinical evidence that is currently lacking. While AI-assisted design holds promise, current applications in actual microdevice fabrication and *in vivo* deployment remain limited and largely conceptual according to the literature.

In recent years, advances in microfabrication have driven significant research toward integrated microsystems, known as micro-total analysis systems (µ-TAS) or lab-on-a-chip (LOC) platforms. These systems integrate sampling, sample preparation, detection, and data processing within a single miniaturized device, enabling functionalities such as cell sorting, lysis, single-cell analysis, and non-destructive cellular studies in individual microreactors. In parallel, tissue engineering has emerged as a promising application area, where microdevices provide controlled microenvironments to regulate cell migration, proliferation, and differentiation, supporting the development of engineered tissues and organ-like constructs for potential implantation.^416^ Lundeberg and co-workers developed a high-throughput, microarray-based barcoding strategy for single-cell RNA sequencing that enables cost-effective profiling of gene expression at single-cell resolution, thereby advancing the study of complex biological processes such as cancer heterogeneity.^417^ Complementarily, Zhang *et al.* developed plasmonic gold-based antibody arrays capable of detecting antigens in as little as 10 µL of human serum using near-infrared fluorescence-enhanced (NIR-FE) detection, achieving superior limits of detection, quantification, and reproducibility compared with the clinically used Luminex platform.^418^ Because precise temperature control underpins the polymerase chain reaction (PCR), significant effort has been devoted to thermal modeling and experimental characterization of temperature fields in PCR microdevices.^419^ While early studies placed limited emphasis on thermal optimization, finite-element modeling has since emerged as a key tool for rational thermal design of PCR microsystems, with subsequent work incorporating genetic algorithms to accelerate optimization of convective PCR devices.^420^ Complementary experimental approaches, including embedded thermocouples, temperature-sensitive liquid crystals, and in-chamber thermal indicators, have enabled validation of modeled temperature distributions and improved control of microscale thermal cycling.^421,422^ However, evidence supporting the successful *in vivo* application of this technique remains limited. Taken together, these studies chart a path toward adaptive, self-powered, and precision microdevices; however, the lack of long-term *in vivo* validation currently limits their translational applicability. With time, systematic biological evaluation and engineering refinement may enable their successful clinical realization.

## 11. Conclusion

Stimuli-responsive microdevices have emerged as transformative tools in drug delivery and biomedical engineering, offering unprecedented control over therapeutic release, spatial targeting, and dynamic responsiveness to physiological cues. By harnessing endogenous triggers such as pH variations, enzymatic activity, and redox gradients, among others, these systems enable on-demand, site-specific drug release, minimizing systemic toxicity and enhancing therapeutic efficacy. Recent innovations in material science, including smart polymers, nanofibers, and hybrid composites, have expanded the functional versatility of these devices, allowing integration with biosensors, wearable platforms, and implantable systems. Their adaptability across diverse clinical contexts, from oncology and wound healing to tissue regeneration and personalized medicine, underscores their translational potential. Integration into clinical workflows is increasingly supported by advances in miniaturization, biocompatible materials, and wireless actuation. In oncology, devices leveraging tumor-specific microenvironments have demonstrated enhanced efficacy and reduced off-target toxicity. In chronic wound care and diabetes management, microdevices enable real-time responsiveness to biochemical fluctuations, improving outcomes through dynamic dosing and localized intervention. Moreover, implantable and wearable formats are being explored for long-term management of neurological and endocrine disorders, with promising results in early-phase trials.

Despite remarkable progress, challenges persist in scaling up fabrication, ensuring long-term biocompatibility, and obtaining regulatory approval. Also, clinical translation faces hurdles, including scalable manufacturing and long-term biosafety. Addressing these challenges requires interdisciplinary collaboration and robust validation frameworks. Future research should focus on multi-stimuli integration, real-time feedback mechanisms, and patient-specific customisation to fully realise the promise of these intelligent systems. As personalized medicine gains momentum, stimuli-responsive microdevices stand poised to become cornerstones of next-generation therapeutics, offering not just treatment but intelligent, adaptive care tailored to individual patient profiles. In sum, stimulus-responsive microdevices represent a paradigm shift toward precision therapeutics, where treatment is not only targeted but also intelligently timed and dynamically tuned to the patient's unique biological landscape.

## Author contributions

Deepanjan Datta: conceptualization, data curation, formal analysis, investigation, project administration, resources software, supervision, validation, visualization, writing – original draft, writing – review & editing. Viola Colaco: data curation, investigation, writing – original draft. Maria Nison: data curation, investigation, writing – original draft, writing – review & editing. Ananya Prabha H: data curation, investigation, writing – original draft. Sony Priyanka Bandi: data curation, investigation, writing—review & editing. Namdev Dhas: formal analysis, visualization. Vasudev R Pai: visualization. Praveen Halagali: data curation, investigation, writing – original draft. Vamshi Krishna Tippavajhala: visualization. Sudarshan Singh: visualization. Lalitkumar K. Vora: conceptualization, formal analysis, visualization.

## Conflicts of interest

The authors declare no competing interests. The views and opinions expressed in this article are purely those of the authors.

## Supplementary Material

RA-016-D5RA09767C-s001

## Data Availability

No primary research results, software or code have been included, and no new data were generated or analysed as part of this review. Supplementary information (SI): Section S4 summarizes fabrication methods that enable the precise structuring of microdevices with complex geometries. Active microdevices and passive microdevices incorporating components that respond to external stimuli or have moving parts for controlled actuation and sensing, and rely on inherent material properties and structural design, functioning without external energy input, typically for filtration, diffusion, or static sensing, respectively, are discussed in Sections S5.1–S5.2. Table S1 contains the summary of stimuli-responsive microdevices and delivery systems categorized by trigger type, device format, and Technology Readiness Level (TRL). The SI file includes the additional references relevant to the discussed content. See DOI: https://doi.org/10.1039/d5ra09767c.
