# Supplementary material for: Smart microdevices for biomedical drug delivery: endogenous stimuli as the key to safer therapeutics
Source: RSC Adv. 2026 Mar 18;16(16):14878–935. doi: 10.1039/d5ra09767c (PMC12997546; doi:10.1039/d5ra09767c)
Supplement: RA-016-D5RA09767C-s001 [file RA-016-D5RA09767C-s001.pdf]

Supplementary Information (SI) for RSC Advances.
This journal is © The Royal Society of Chemistry 2026

Smart Microdevices for Biomedical Drug Delivery: Endogenous Stimuli as the Key to Safer Therapeutics

Deepanjan Datta[1*], Viola Colaco[1], Maria Nison[2], Ananya Prabha H[1], Sony Priyanka Bandi[3], Namdev Dhas[1], Vasudev R Pai[2], Praveen Halagali[1], Vamshi Krishna Tippavajhala[1], Sudarshan Singh[4,5], Lalitkumar K. Vora[6]

[1]Department of Pharmaceutics, Manipal College of Pharmaceutical Sciences, Manipal Academy of Higher Education, Manipal, India

[2]Department of Pharmacognosy, Manipal College of Pharmaceutical Sciences, Manipal Academy of Higher Education, Manipal, India

[3]Department of Pharmacy, Birla Institute of Technology and Science (BITS) Pilani, Hyderabad Campus, Hyderabad 500 078, Telangana State, India

[4]Faculty of Pharmacy, Chiang Mai University, Chiang Mai 50200, Thailand

[5]Office of Research Administration, Chiang Mai University, Chiang Mai 50200, Thailand

[6]School of Pharmacy, Queen's University Belfast, 97 Lisburn Road, Belfast BT9 7BL, UK

***Authors to whom correspondence is to be addressed**

Deepanjan Datta, PhD
Assistant Professor
Department of Pharmaceutics
Manipal College of Pharmaceutical Sciences
Manipal Academy of Higher Education, Manipal 576 104, Karnataka State, India
Email: deepanjan.datta@manipal.edu; deepanjandtt@gmail.com

SUPPLEMENTARY INFORMATION

S4.1. Continuous liquid interface production (CLIP)

CLIP is a photopolymerization-based 3D printing technique that eliminates the recoating step by using an oxygen-permeable window to form a "dead zone", allowing continuous polymerization and rapid vertical layer construction at speeds up to 30 cm/h. Despite its speed and ability to reduce the staircase effect, it has limitations, including lower resolution (75 μm) and the need for lower-viscosity resins, restricting material choices [1–3].

S4.2. Laser-induced forward transfer (LIFT)

LIFT is a precise material transfer technique using a pulsed laser to propel material from a donor ink layer to an acceptor substrate, enabling high-resolution patterning and 3D structure fabrication. The donor layer can be liquid, solid, or paste, allowing the use of various materials such as metal nanoparticles, hydrogels and biomaterials. LIFT offers resolution up to 10 μm and supports 2D/3D patterning. It has expanded to applications in sensors, capacitors, and bioprinting. Challenges include maintaining a minimal donor-to-acceptor distance and weak adhesion of transferred materials, limiting its use in microrobotics. Nonetheless, LIFT's broad material compatibility, high resolution, and versatility make it a promising tool for advanced technologies [4–6].

S4.3. Inkjet printing (IJP)

IJP is a non-contact fabrication method that selectively deposits droplets (3-100 pL) onto substrates, allowing rapid evaporation to immobilize materials. It supports diverse inks, including piezoelectric, conductive, insulating, biocompatible, and enzyme-based formulations, making it adaptable for various applications. However, challenges like limited resolution (~50 μm), poor droplet placement control, and low interlayer adhesion restrict its utility for sub-10-micron precision tasks [4,5,7].

S4.4. Stereolithography (SLA)

SLA is a foundational laser-based microfabrication technique that operates by utilizing a UV laser to cure a liquid monomeric resin into a solid structure. The process involves directing a laser beam at a tank containing photocurable liquid resin while a motorized stage facilitates vertical movement. The laser-induced cross-linking polymerization in the resin solidifies a predetermined pattern layer-by-layer. After fabrication, the unpolarized resin is removed

during a post-print development step [8,9]. Despite its high resolution (100 nm) and compatibility with biodegradable resins, limitations in accuracy, material diversity, and fabrication speed restrict its direct application in microrobot production [10]M. Valentin et al. demonstrated SLA 3D printing of alginate hydrogel using ionic crosslinking with divalent cations (Ba^{2+}, Ca^{2+}) to achieve tunable degradation, mechanical properties, and pattern fidelity [11]. In another study by Ziesmer and colleagues, they presented a method for fabricating photothermal MNs using a low-cost SLA printer with plasmonic Ag/SiO_2 nanoaggregates in ultraviolet photocurable resin [12]. The resulting MN arrays demonstrated sufficient mechanical strength, heating efficiency, and the ability to increase intradermal temperature to clinically relevant levels under NIR irradiation. The photothermal MNs showed effectively reduced bacterial growth in skin infections and can be customized in size, shape, and needle geometry, offering promise in treating bacterial skin infections (**Figure S1)**.

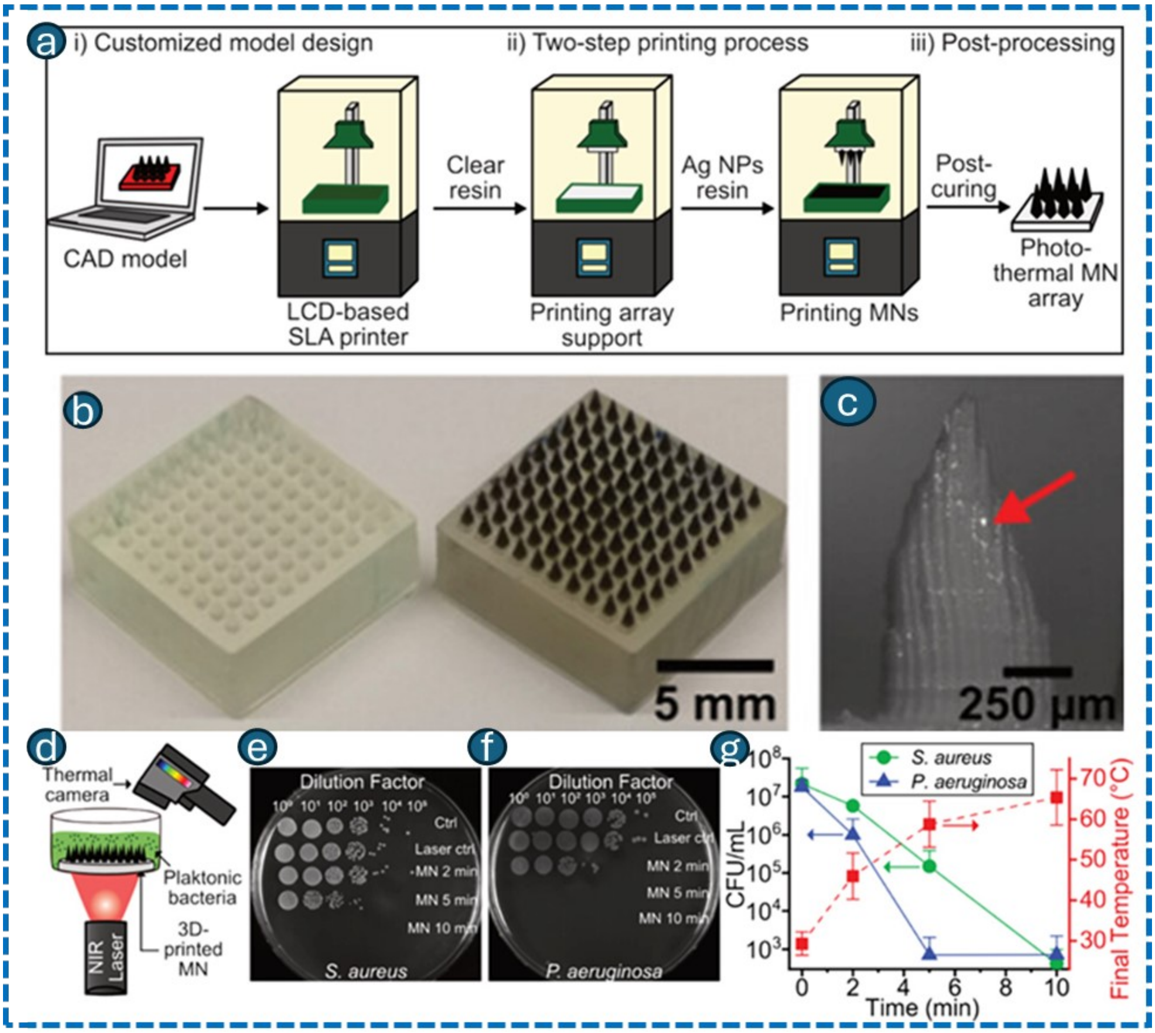

Figure S1. Schematic illustration of the two-step 3D printing process for photothermal microneedle (MN) arrays: configuration of a customizable MN design onto an LCD-based SLA

printer (a.i), array support printing with clear UV resin and needles with plasmonic Ag/SiO_2 (2 wt% SiO_2) NP suspended in UV resin (a.ii), and post-curing in UV for 10 min (a.iii). Digital images of different-shaped 3D-printed photothermal MN arrays with an Ag/SiO_2 NP-free MN array (b). SEM image of a single photothermal MN indicating regions with silver-rich surface (red arrow) (c). Antibacterial activity evaluation noted: schematic of the arrangement for evaluation of temperature and antibacterial effects under 808 nm NIR irradiation (1 W cm^{-2}) (d), agar plates showing serial dilutions of *S. aureus* (e) and *P. aeruginosa* (f) treated with MN arrays at different time durations (0, 2, 5, 10 min), bacterial quantity (CFU mL^{-1}) of S. aureus (green) and P. aeruginosa (blue) irradiated continuously along the final temperature profile (red, n = 6) (g). The data are indicated as mean ± SD (n = 3). Reproduced from [12]. Copyright 2024, American Chemical Society.

S4.5. Digital light processing (DLP)

DLP is a photopolymerization-based microfabrication technique that rapidly creates 3D structures by projecting UV light onto entire resin layers simultaneously. Unlike SLA, this approach enhances printing speed and reduces oxygen interference. The limitations of DLP include restricted choices of materials and a low-resolution range (25-100 μm) [12–14]. DLP has been widely used in microfluidic devices [15] and allows the incorporation of functional nanoparticles, such as carbon nanotubes, to enhance material properties [55]. A study by Wang et al. developed poly(acrylic acid) (PAA)-based electroactive actuators fabricated using DL for soft robotic applications [15]. The actuators respond to a low electric field (4-6 V), exhibiting large deformation (43°), fast actuation (1.08 °/s), and stable performance. Enhanced actuation was achieved with higher carboxyl group concentrations and thinner geometries, while high cross-linking density increased mechanical strength and viscoelastic behaviour. A functional membrane valve actuator was demonstrated, showcasing the potential of these tunable, cost-effective materials for developing complex 3D-printed actuators for artificial muscles and soft robotic systems. In another study, Gugulothu and Chatterjee introduced a cytocompatibility bioink based on gelatin methacryloyl (GelMA) and poly(ethylene glycol) dimethacrylate (PEGDM) for DLP-based 4D bioprinting using visible light (405 nm) photopolymerization (Gugulothu and Chatterjee, 2023). The bioink enables hydration-induced shape morphing, driven by differential cross-linking through photoabsorbers. The printed constructs mimic soft tissue mechanics, support complex geometries, and maintain high cell viability and proliferation, with NIH/3T3 cells attaining fibroblastic morphology. The findings position the

constructs as promising candidates for tissue engineering applications, including vascular grafts, regeneration models and soft robotics **(Figure S2)**.

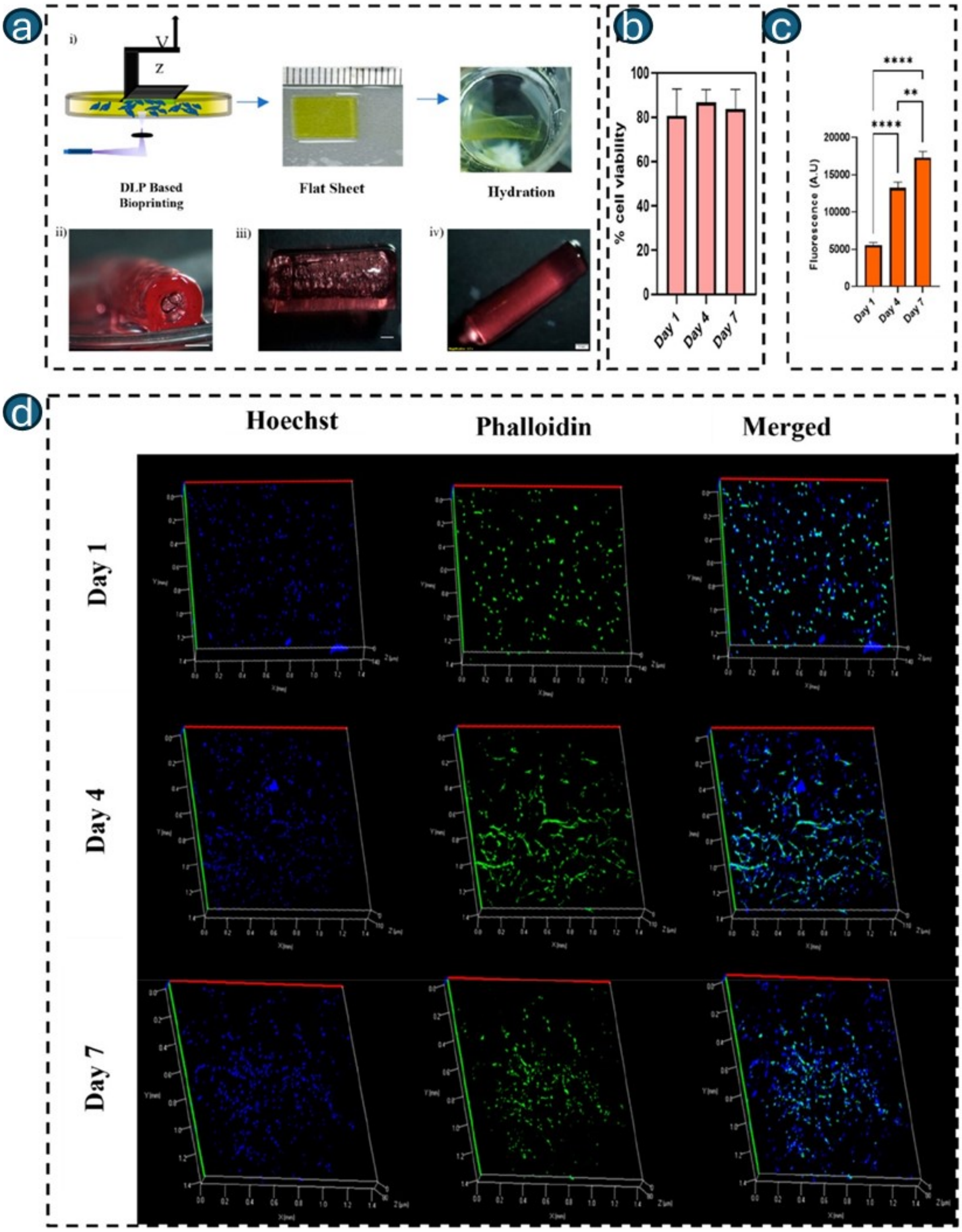

Figure S2. Biological characterization and Shape Deformability of 4D-Bioprinted Scaffolds: Schematic representation of 3D bioprinting of a 10 × 10 × 0.8 mm^3 gel using P2.5G12.5 bioink with NIH/3T3 cells (a. i), and shape transformation after 1-hour equilibration in media (scale bar = 1 mm) (a.ii-iv). Metabolic activity of NIH/3T3 cells monitored over 7 days (b). The percentage of living cells at various times was assessed via their Calcein AM (green) and ethidium homodimer staining (c). 3D fluorescence imaging showing cytoskeleton (green) and

nucleus (blue), illustrating the morphological transition from spherical to fibroblastic shape (d). Reproduced from [16]. Copyright 2023, American Chemical Society.

S4.6. Two-photon polymerization (TPP)

TPP, also known as direct laser writing (DLW), is a high-resolution 3D microfabrication technique using an 800 nm femtosecond laser to induce nonlinear two-photon absorption in photopolymer. This allows precise, localized polymerization at the laser's focal point, enabling submicron resolution (<50 nm feature size and <150 nm lateral resolution) and exceptional geometric flexibility [17,18]. TPP supports both additive and subtractive manufacturing and has applications in microrobotics [19] and micro-electro-mechanical systems (MEMS) [20]. However, challenges include slow fabrication speed, high costs, and limited structure height (<1mm). Despite these, TPP shows promise in fields like drug delivery, cell manipulation, and microsurgery, with ongoing efforts to enhance scalability and efficiency [5]. Isaakidou et al. developed and characterized cochlear implants with anatomically relevant dimensions and tunable microporous structures for localized drug delivery [21]. Using TPP with IP-Q resin, implants with rectangular and cylindrical structures were fabricated, featuring precise pore sizes (20 μm and 60 μm), partial hydrophilicity (contact angle: 72.3), and high polymerization efficiency. The implants demonstrated excellent cytocompatibility, offering a novel approach for targeted, personalized hearing loss treatment.

S4.7. Selective laser sintering (SLS)

SLS uses a laser to sinter fine powder particles below their melting point, fabricating 3D structures layer-by-layer with isotropic mechanical properties. The technique involves three key steps: powder deposition, laser-induced powder solidification, and vertical lowering of the fabrication platform by one layer thickness. These steps are repeated iteratively to build the desired 3D object [7,22,23]. Reusable powder particles enable 3D structures with 45 μm resolution. SLS resolution depends on particle size, laser intensity, and scanning precision, with conventional systems achieving 100-250 μm and advanced micro-SLS systems below 5 μm [6]. The limitations of the SLS technique include limited material options, high porosity, low biocompatibility, rough surfaces, and elevated power and temperature requirements. Despite limitations, SLS offers minimal anisotropy, complex structural fabrication, and reusable support powders, showing potential for high-resolution microrobotics and biomedical applications with ongoing advancements [4,7,24]. Mei and colleagues fabricated thermoplastic polyamide elastomer (TAPE) film using SLS technology and demonstrated stable, repeatable,

and responsive behaviour for organic vapour sensing [25]. The microphase-separated structure enabled vapour absorption, forming ionic pathways and reducing resistance, particularly for polar solvents. These films also detected ethanol in breath monitoring, highlighting the potential in wearable sensors and 4D printing.

S4.8. Fused deposition modeling (FDM)

FDM involves melting thermoplastic materials and extruding them through a nozzle to fabricate 3D structures. It enables the cost-effective construction of complex shapes and allows multi-material fabrication using multi-nozzle systems. FDM's limitations encompass reduced resolution, elevated printing temperatures, limited material options, and a layer-by-layer finish [4,26,27]. Lin et al. developed a 4D-printed, patient-specific, bioinspired absorbable left atrial appendage occluder (LAAO) to address mechanical mismatches and biocompatibility issues of traditional devices [28]. The actuator was composed of PLA and Fe3O4 nanoparticles. The LAAO mimics LAA tissue mechanics using an optimized network design, offering tailored geometry and a shape-memory transformation activated by minimally invasive magnetic field implantation. The *in vivo* biocompatibility of sterilized 10PLA-MNC occluders was evaluated by subcutaneous implantation in male Sprague-Dawley mice for 48 weeks. It demonstrated excellent durability, biocompatibility after 48 weeks of implantation, and feasibility in preclinical swine heart tests. Another study by Mousavi et al. presented a 3D-printed multidirectional strain sensor for soft robotics [29]. Using a PLA-CNT composite, sensors and conductive interconnects were directly printed with high sensitivity (gauge factor ~1342) and selectivity (31.4x), enabled by a weak bond track design. Customization of sensitivity and anisotropy is achieved by modifying air gaps, infill density, and build orientation. The sensors can distinguish tensile and compressive bending, integrate seamlessly into soft robots, and bypass the challenges of traditional mounting techniques. Despite low stretchability due to material brittleness, placement near the axis mitigates this issue, paving the way for cost-effective, multifunctional soft robotic systems.

S4.9. Microextrusion 3D printing

Microextrusion 3D printing constructs layer-by-layer structures with at least one dimension in the micrometre range. The printable materials are extruded through a nozzle via mechanical, electrical, or acoustic actuators, enabling continuous filament deposition. The advantages include cost-effectiveness, solvent-free operation, high material viscosity compatibility, and suitability for high cell density applications. However, it has limitations like cell damage during

printing, and restricted resolution hinders its application for finer structures [4,7]. Zhang et al. developed a 3D-printed gelatin/bioceramics core/shell scaffold for treating an osteosarcoma-related bone defect [30]. This study presented the development of 3D-printed scaffolds made of bioceramics and gelatin, with doxorubicin (DOX)-loaded gelatin serving as the core and SrCuSi4O10 (SC) nanosheets/beta-tricalcium phosphate (β-TCP) serving as the shell. SC nanosheets provided photothermal treatment to the scaffolds when exposed to NIR-II laser light. At the same time, the produced extreme heat might cause the gel-to-sol transition of the gelatin in the filaments' central core, which in turn causes the loosening of the gelatin to release DOX on demand, allowing for chemo-photothermal treatment. Synergistic anti-tumor effects were shown by *in vitro* and *in vivo* studies demonstrating chemo-photothermal therapy **(Figure S3)**. Furthermore, the scaffold's hollow channels were formed by the disintegration and release of gelatin from the filaments, which served as visible architectural signals to encourage the ingrowth of bone tissues. Degradation of SC nanosheets also helped to liberate bioactive ions (Sr, Cu, and Si) over time, which boosted vascularized bone regeneration even more. These findings pointed to the promising future of 3D-printed DOX-loaded gelatin-TCP/SC scaffolds as a tool in the fight against osteosarcoma by destroying cancer cells and healing tumor-induced bone deformities. Overall, the sequential treatment approach (anti-tumor therapy followed by bone defect reconstruction), closely resembling the real clinical management of osteosarcoma and aligned with the phased treatment protocols employed by orthopaedic surgeons, was underscored in this study.

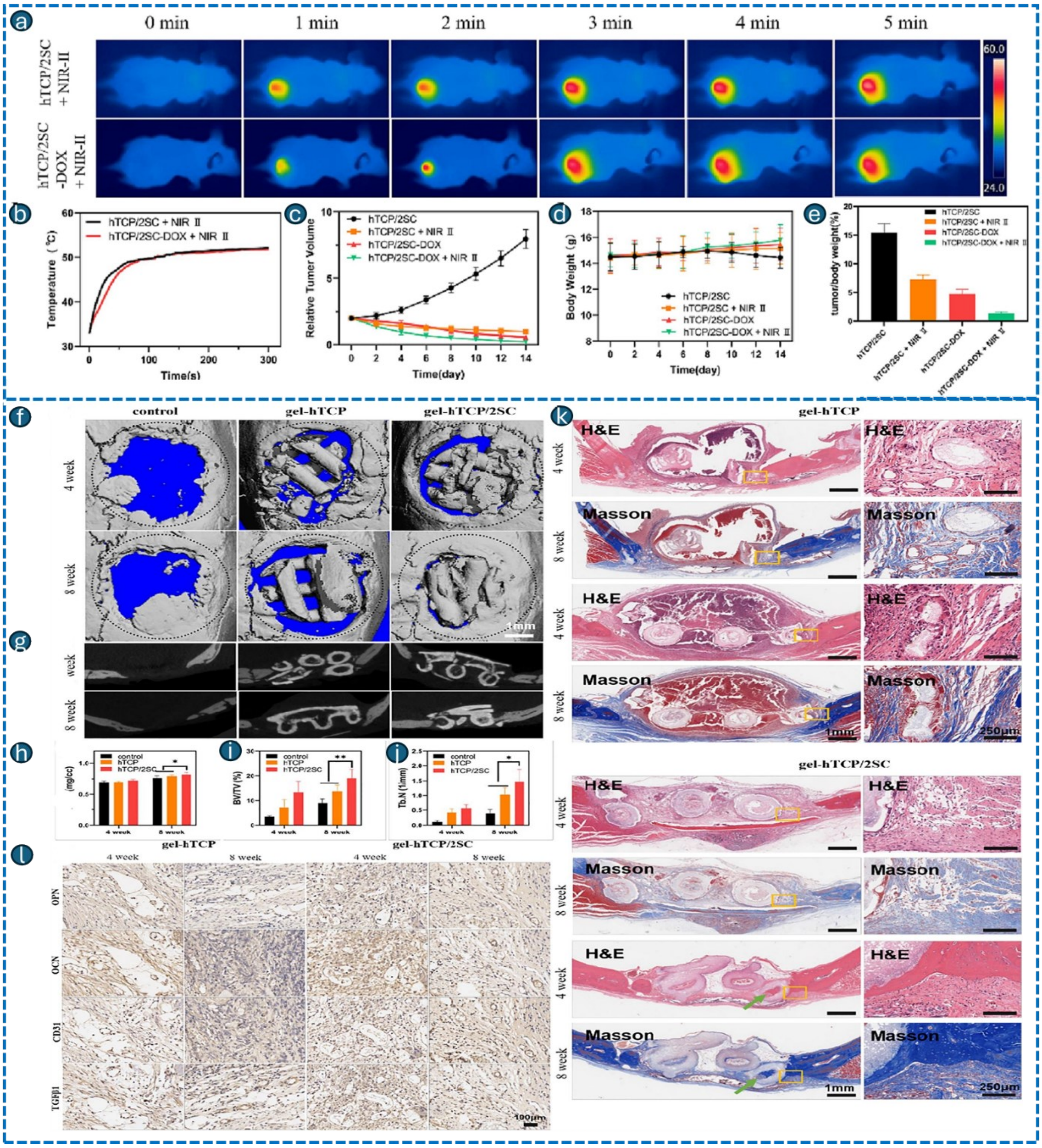

Figure S3. *In vivo* antitumor efficacy. Thermal images (a) and their corresponding photothermal heating curves (b) of *in vivo* 3D printed hTCP/2SC and hTCP/2SC-DOX composite scaffolds under 1064 nm laser irradiation (1.5 W/cm2, 5 min). Relative tumor volume (c), body weight (d), and tumor/body weight ratio (e) in mice after different treatments for 14 days. Evaluation of *in vivo* bone regeneration: 3D reconstruction of micro-CT images showing new bone formation in defect areas after different treatments for 4 and 8 weeks (f); and sectional views (g). Quantitative analysis of bone mineral density (BMD) (h), bone volume/tissue volume (BV/TV) (i), and trabecular bone number (Tb. N) (j) in the region of interest. H&E staining and Masson's trichrome staining of cranial defect sites after implantation

of 3D-printed gel-hTCP and gel-hTCP/2SC core/shell composite scaffolds for 4 and 8 weeks (k). Immunohistochemical staining of newly formed tissues targeting OPN, OCN, CD31, and TGFβ1 for treatment with gel-hTCP and gel-hTCP/2SC core/shell composite scaffolds for 4 and 8 weeks (l). Data expressed as mean ± SD (n = 5). Adapted with permission [30]. Copyright 2023, Elsevier.

Taken together, AM and 4D printing have very versatile and breakthrough technology in crafting micro-devices owing to high control of complex geometries and multi-material integration. Most notably, the development of such multi-stimuli-responsive systems augments functionality both in biomedical and industrial applications, paving the pathways leading to advanced innovations in precision medicine, soft robotics, and regenerative therapies. Research and development of multi-purpose devices tailored to certain medical uses, such as photothermal microneedles loaded with antimicrobial agents, gentle robotic sensors, and bio-inks appropriate for tissue engineering. For instance, the incorporation of appropriate functional materials, such as nanoparticles and stimulus-responsive polymers, makes them exceedingly versatile in drug delivery approaches, cancer treatment, and regenerative medicine. The continuous developments in material science and process optimization promise a slew of innovative applications in health care and beyond.

S5.1. Passive devices

The principle of passive-delivery microdevices is the administration of a medication using implant designs with one or more reservoirs. Reservoirs are hermetically sealed from the delivery site by a polymeric membrane, which controls the activation and timing of release. The substance of the membrane reacts chemically or biochemically with its surrounding environment at the place of implantation to regulate the rate of membrane breakdown. Drugs can be slowly and steadily released over an extended time using passive-delivery devices, which rely on diffusion or osmotic pressure to transport the payload. Examples include the fentanyl transdermal system (Duragesic) and the fluocinolone acetonide intravitreal implant (Retisert), streamlining treatment delivery without integrated electronics. Because of the lack of dose dependence and the fact that the medication distribution does not necessitate active feedback control or telemetric activation, these devices are mostly reserved for the long-term, continuous treatment of chronic conditions. The main benefits of this passive-delivery microdevice type are its compact size, biocompatibility, huge payload capacity, ease of

operation, and ability to load a range of drug formulations. It also lacks the requirement for supporting electronics [31].

At present, clinical applications make use of biodegradable polymers as passive-delivery systems. After brain tumor removal, the residual tumor tissues can be treated with the chemotherapeutic medication 1,3-bis(2-chloroethyl)-1-nitroso-urea (BCNU) (carmustine), administered locally over three weeks using Gliadel polymer wafers [32]. The hydrolysis of the biodegradable polymer causes the medication reservoirs in these delivery systems to gently discharge their contents into the body. Controlled-release implantable devices can be engineered from environmentally responsive hydrogels. Baldi et al. developed microvalves made of double-sided structures with silicon membranes responsive to the stimulus of pH, temperature, glucose concentration, etc., with higher response times than the existing systems. These developed microvalves could be incorporated into autonomous systems that facilitate reaction cycles inside microreaction chambers without the requirement of extra controlling electronics [33]. Diabetes, exacerbated due to the rise of obesity rates, poses significant challenges to global healthcare, especially due to poor adherence to insulin administration. Glucose-responsive hydrogels present a promising avenue for controlled insulin delivery, enhancing clinical reliability and effectiveness, while challenging researchers to refine response kinetics and advance engineering of these systems. [34]. Passive resorbable millimetre-sized devices constructed of compression-molded poly(l-lactic) acid (PLLA) reservoirs with poly(lactide-co-glycolide) membranes were created by Grayson et al., as another form of passive delivery device [35]. The medication was released over time as the multiple-reservoir membranes broke down after implantation. Degradation of PLLA-based devices occurred gradually over several months, enabling the release of medications and other biological chemicals across the membrane prior to the complete absorption of the device at the implant site. These molecules include heparin, human growth hormone, and dextran. Release via the poly(lactide-co-glycolide) membranes occurred as water permeated the polymer, resulting in swelling and hydrolysis. Polymers with higher molecular weights often require more time for complete degradation, facilitating pulsatile and timely drug release through the incorporation of various molecular weights in the membrane polymers.

To aggressively treat malignant gliomas, innovative passive-delivery devices were developed. These devices have shown higher chemotherapeutic effectiveness of temozolomide compared to BCNU (carmustine). The great efficacy of chemotherapeutic drugs like temozolomide, O6-benzylguanine, and STAT3 inhibitor III in treating glioblastomas suggests their possible usage

with implantable drug-delivery systems. Future research on passive-delivery devices will explore methods for delivering multiple drugs simultaneously from multiple reservoirs, enhancing treatment efficacy for chronic illnesses requiring minimally invasive and localized drug delivery.

S5.2. Active devices

These utilize numerous mechanisms for the controlled release of therapeutic agents, including micropumps driven by magnetic actuators, gas pressure, and electrothermal or electrochemical systems. These devices provide customized options for treating various diseases and can be either activated or deactivated after implantation. But these systems usually require miniature power electronics that increase the overall size of the system, which is mitigated by a telemetry system. Moreover, active devices provide precise control of the pharmacokinetic profile of drug delivery, unlike passive devices. The development of active-delivery devices using microelectrochemical systems (MEMS) technology was first intended for controlled-release applications. Early devices used electrochemical dissolution of gold or polymeric membranes to transport drugs to the site of action [36]. Pulsatile and regulated drug release from numerous reservoirs was possible with selective membrane activation. MEMS-based devices could precisely release several agents from a single device [37,38]. Li et al. subsequently showed *in vivo* delivery of the chemotherapeutic agent BCNU in rats using this technique [39].

S5.2.1. Multifunctional MEMS devices

Multifunctional MEMS devices were developed for the simultaneous recording of neural activity and drug delivery. Altuna et al. prepared microprobes that are flexible by utilizing SU-8 polymer, incorporated electrodes made of platinum for sensing, and a microfluidic channel responsible for delivering the drug. Single-channelled probes like tetrodes and dual-channelled probes like linear probes were tested in live rats, and it was observed that these delivered therapeutic agents to brain-targeted areas, simultaneously recording neuronal activity, including spikes and ripples, demonstrating their flexibility in neuroscience research [40,41]. Despite decades of management of neurological diseases like Parkinson's and epilepsy with L-DOPA and anti-epileptic drugs, effective treatments remain elusive. Implantable devices such as neural stimulators arose as promising options; however, certain challenges, like poor biocompatibility leading to fibrosis and inflammation, need to be faced. A combination of drug delivery systems and neural probes using technologies like MEMS offers a potential solution

by providing simultaneous neural data recording as well as targeted drug delivery, addressing symptoms as well as adverse effects [42,43].

S5.2.2. Electrothermally actuated MEMS microchips

Electrothermally actuated MEMS microchips are intriguing gadgets that utilize thermal expansion to generate mechanical motion at the microscale. These are miniature mechanical systems embedded within microchips. Actuation is accomplished using Joule heating, which involves sending current through a microstructure to produce heat. Thermal expansion in certain materials induced by heat leads to movement. Generally, it entails bimorph structures, comprising two layers of materials with disparate thermal expansion coefficients. Upon heating, one layer expands more significantly than the other, resulting in the structure bending or twisting. Such a device was created for the intracranial delivery of drugs for the treatment of malignant tumors in the brain [44]. This device employed an active MEMS approach to improve drug release kinetics, potentially boosting efficacy and lowering toxicity. The fundamental technique involved the melting of silicon nitride membranes through resistive heating, facilitating a controlled drug release pattern, with each membrane providing a tailored drug release configuration when activated individually.

Glioblastoma is considered a highly aggressive brain cancer form with low survival rates, partially due to the challenges faced in drug delivery across the blood-brain barrier (BBB) [45]. The effectiveness of local implants remained limited, which were developed to bypass the BBB; hence, an active microchip implant was developed, offering controlled drug delivery in the brain [46]. However, refilling or a source of power exchange is not favourable for CNS implants, as it involves risky procedures such as neurosurgery. This led to the development of a MEMS device run electrothermally, showing its effectiveness *in vitro* as well as *in vivo* by increasing the survival rates and controlling drug release in glioblastoma-induced rats.

Santini et al. developed an electrothermally actuated MEMS microchip that enables pulsative and controlled release of chemicals from either multiple or single reservoirs [37]. A solid-state silicon microchip may release one or more chemicals on demand. Thin anode membranes covering microreservoirs packed with solid, liquid, or gel substances dissolve electrochemically to release them. A prototype microchip using gold and saline solution as the electrode material and release medium has shown regulated, pulsatile chemical release in proof-of-principle release trials. Release studies showed that reservoir activation can be modulated separately, enabling complicated release patterns. A gadget with a small size, fast

response times, and low power consumption may have other benefits. All chemical molecules to be released are stored in the microchip's reservoirs, enabling autonomous gadget development. Depending on the application, a microbattery, multiplexing circuitry, and memory could be integrated directly onto the device, allowing it to be mounted on a small probe, implanted, swallowed, integrated with microfluidic components to create a ‘laboratory-on-a-chip,’ or added to a standard electronic package. This innovation is followed by potential research showcasing the advancements of MEMS drug delivery systems in treating several ailments.

Postmenopausal women with low estrogen levels are at risk of osteoporosis, which is characterized by reduced bone density and degradation of bones, with a higher risk of fracture. A microchip device was developed that delivers parathyroid hormone, which stimulates the formation of bones. Human clinical trials performed on postmenopausal women confirmed the bioequivalence and safety of microchips to that of the standard subcutaneous delivery injections [47].

Massive blood loss and severe trauma often result in hemorrhagic shock, which needs immediate treatment to prevent death or any permanent damage. During limited healthcare facility access, hemostatic dressings are often self-applied, but they may not cure internal bleeding. Inotropic agents and vasopressin become crucial to managing this hemorrhagic shock. Hence, a biomedical microdevice was coined to treat hemorrhagic shock for emergency and ambulatory services, where this device treats high-risk patients by rapidly delivering vasopressin and holds the capability to address several acute conditions, such as neurological and cardiovascular emergencies [47]. A novel generation of drug-delivery devices has been developed expressly for trauma care, where swift intervention is essential for patient life. The implantable rapid-drug-delivery device (IRD^3) was designed for the prompt treatment of hemorrhagic shock, utilizing vasopressin as a prototype medication [48]. The device was created as a prophylactic subcutaneous implant for high-risk individuals, such as troops in combat zones. The design includes a triggering algorithm that integrates blood pressure and heart rate data from conventional ECG and blood pressure sensors. It was comprised of a membrane, reservoir, and actuation layers. Microresistors present in the actuation layer produced heat by the passage of current, which induced the formation of bubbles and a rise in internal pressure, eventually rupturing the membrane and releasing the drug bolus swiftly.

S5.2.3. Magnetically actuated MEMS drug delivery system

A magnetically actuated MEMS drug delivery system was created to ensure regulated delivery of chemotherapeutics. This device includes a magnetic membrane composite sealed microreservoir. Membrane deflection is prompted by an external magnetic field, due to which the internal pressure of the reservoir is increased, eventually facilitating diffusion of the drug via micron-sized pores [49]. An anti-cancer drug, docetaxel, whose mechanism of action is the disruption of the mitotic spindle, was selected to assess the device's release profile [50,51]. The application of this device ranges from the treatment of various tumors, such as breast cancer, emphasizing its necessity in local drug delivery and improved efficacy with minimal systemic exposure. A freshly prepared docetaxel solution was compared *in vitro* with the drug released through this device, and the results were matched, indicating the capability of utilising this drug for clinical use, as well as further research and development.

S5.2.4. Microfluidic hydraulic MEMS-based drug delivery devices

Microfluidic hydraulic MEMS-based drug delivery devices run by stimulation via a mechanism that was developed to deliver drugs within the inner ear using a microfluidic circuit connected to microcannulas that enable the flow of fluid outside and inside the cochlea. The compound dissolved within the fluid is delivered efficiently due to the rapid recharge and discharge of fluid caused by different tubing sizes. This system offers a new pathway to its utilization and study in clinical application, as it demonstrates an alteration in the action potential threshold of the compound, providing effective penetration of drugs [52,53]. Sensorineural hearing loss is a condition that affects millions of people in the world. It encompasses various conditions that affect the cochlea and vestibular parts of the inner ear, caused by genetic mutations, infections, drug exposure, trauma, etc., eventually leading to cochlear hair cell death. Cochlear implants provided various advancements in deafness treatment; however, they lost efficacy due to the degeneration of cells. Novel devices such as reciprocating micropumps showcase promising options to deliver treatment compounds without affecting cochlear function and restoring perception to audio [54,55].

Taken together, to meet the growing demand for minimally invasive drug delivery methods, innovative implantable biomedical microdevices have been developed through advancements in MEMS and miniaturization technologies. These devices, both active and passive, can administer precise doses of therapeutic agents at controlled intervals. The preceding sections highlight the potential of these microdevices to enhance traditional approaches for managing both acute and chronic conditions. With their highly engineered designs and capacity to tailor

individual pharmacokinetic profiles, they offer a unique platform for efficient, personalized treatment with minimal invasiveness.

Table S1. Summary of stimuli-responsive microdevices and delivery systems categorized by trigger type, device format, and Technology Readiness Level (TRL).

Stimulus	Microdevice	Current TRL	Evidence supporting TRL	Translational Gap	Application	Ref.
Temperature	Disposable microfluidic Device (2015)	TRL 3–4	Demonstrated controlled temperatures (37/42/50 °C, ±0.3 °C), increased apoptosis with MHIFU vs. incubation at 42 °C, and AFM-observed membrane *changes in vitro* studies	Lack of *in vivo* validation, HIFU-TSL dosing/thermometry & device scale-up pending	Model temperature-triggered chemotherapeutic release and cell-level effects	56
	Microparticle-based microdevice (2017)	TRL 2–3	Supercritical fluid emulsion fabrication of magnetized PLGA/PLA microparticles is an established lab-scale method, surface coating enables tunable release, *in vitro* characterization typical for such systems; analogous microfluidic and microdevice platforms remain early-stage research tools	Lack of *in vivo* navigation validation studies	Targeted drug delivery with controlled release	57
	Soft microrobot (2018)	TRL 2–3	Demonstrated *in vitro* with basic motion control and stimulus-responsive swelling, no evidence of animal validation	Complex navigation in *in vivo* studies, biocompatibility of long-term magnetic	Controlled local drug delivery (Proof-of-concept)	58

				actuation, lack of scalable fabrication, and no preclinical safety studies		
	Microfluidic hydrogel-based microcarriers (2018)	TRL 3	Microfluidic fabrication and temperature-dependent release demonstrated *in vitro*, with no *in vivo* validation	Scaling microfluidic production, stability of thermo-responsive behaviour in physiological conditions, lack of biocompatibility and pharmacokinetic data	Controlled drug release, tunable thermos-responsive carrier system	59
	Actuators (2018)	TRL 2–3	Demonstrated reversible hydrogel actuation, on-chip valve control, and tunable deformation in *in vitro* microfluidic prototypes, consistent with PNIPAM-based thermoresponsive device behaviour in microfabricated systems	Not intended for clinical use; poor long-term stability of thermogels, slow response time, challenges in scalability, sterilization, and integration with	Programmable microfluidic control, biomolecular handling, soft micro-robotics, lab-on-chip actuation	60

				biomedical workflows, no *in vivo* validation		
	hMSC–microdevice complex (2019)	TRL 3	Controlled and versatile attachment to hMSCs, complexes are stable after cultivation and trypsinization, no loss of proliferation, retained migration and spheroid behaviours in all *in vitro* studies	No *in vivo* validation, homing efficiency and biodistribution of hMSCs are known translational hurdles, need GMP-compliant microdevice fabrication and cell-product release testing; regulatory path for cell+device combination remains complex	Targeted/escort delivery using hMSCs while preserving motility and viability	61
	Microinjectors (2020)	TRL 3–4	*In vitro* penetration through GI-mimicking hydrogels and *ex vivo* intestinal tissues, some prototypes tested in small animals, demonstrating successful mucosal insertion and	Requires safety validation for GI perforation risk, reproducible actuation timing, scalable	Oral delivery of biologics by trans-mucosal injection in the intestine	62

			localized drug release	microfabrication, biocompatible dissolution of components, and comprehensive *in vivo* pharmacokinetic studies prior to regulatory progression		
	Microneedles (2022)	TRL 3–4	Demonstrated microneedle mechanical insertion capacity, temperature-triggered swelling, and controlled drug release *in vitro*, some systems show *in vivo* animal pharmacodynamic enhancement and improved delivery efficiency	Need standardized mechanical robustness under physiological conditions, large-scale reproducibility, long-term stability of thermo-responsive polymers, safety validation for repeated skin application, and regulatory clarity for stimuli-responsive MNs	Controlled transdermal drug delivery	63

	3D-printed PNAGA hydrogel microrobot (2023)	TRL 3–4	Demonstrated 3D printing feasibility, thermo-responsive drug release, and *in vitro* cytotoxicity, lack of *in vivo* tumour model testing	Complex *in vivo* navigation and targeting, long-term biocompatibility studies, limited understanding of tumor penetration, scalability and regulatory challenges for micro-robotic devices	Cancer therapy via temperature-triggered drug release	64
pH	Implantable pH-responsive drug-delivery device (2009)	TRL 3–4	Extensive *in vitro* data and preclinical studies reported for pH-responsive hydrogels, implant design frameworks, modeling, and materials are well documented for implantable controlled release	Long-term biostability/degradation and mechanical robustness *in vivo* batch-consistent pH response, tissue–device integration, fouling, and sterilization; predictive in-human	Site-specific, on-demand release in acidic microenvironments, long-acting local therapy	65

				release models, regulatory path for responsive implants vs. conventional long-acting depots		
	Hydrogel-coated microcantilever sensing arrays (2011)	TRL 2–3	Behaviour consistent with hydrogel-based microenvironment-responsive systems that demonstrate responsive swelling and biochemical sensitivity strictly *in vitro* without translational validation	No *in vivo* testing, challenges with hydrogel stability, cantilever drift, sterilization, long-term performance in physiological fluids, limited scalability, no regulatory pathway	Label-free detection of pH changes, analytes, and biochemical microenvironment cues, environmental/biomedical sensing	66
	Microspheres (2012)	TRL 3–4	Fabrication of pH-responsive PLGA hollow microspheres, stable encapsulation of multiple drugs, MN mechanical strength sufficient for rat skin insertion, rapid dissolution of PVP MNs depositing HMNs, pH-triggered	Requires optimization for drug loading uniformity, long-term HM stability, and reproducibility of CO_2-triggered rupture	Smart transdermal multidrug delivery with two-stage, sequential release for combinational or staged therapy	67

			CO_2-mediated rupture causing controlled secondary drug release, and successful in-skin localization and diffusion of HMs	in more complex human skin pH environments. No large-animal or chronic-use safety studies, scaling PLGA HMN fabrication and MN integration under GMP is nontrivial. The regulatory pathway for multistage, reactive MNs systems remains undefined		

	Soft micro-robot (2016)	TRL 3–4	Supports align with established fields: microfluidic drug-delivery devices enabling precise actuation and targeting, and pH-responsive hydrogels validated for controlled, environment-triggered drug release in preclinical contexts	Requires integration of magnetic actuation, hydrogel release, and biocompatible encapsulation. Challenges include *in vivo* navigation, immune clearance, biodegradation control, imaging guidance, and regulatory pathways for magnetically actuated implants	Site-specific delivery in tumor or inflamed acidic microenvironments	68

	Microneedle (2018)	TRL 4	Study shows show *in vivo* pharmacodynamics evaluation, greater anti-OVA IgG1 and $CD8^+$ T-cells vs soluble DNA vaccine and tumor rejection in B16/OVA melanoma; mechanism and composition detailed (OSM–(PEG-PAEU), poly(I:C), LbL MN coating)	Needs GMP-scalable MN manufacturing, sterilization, coating uniformity & shelf-life, human skin translation, dose-finding & safety; regulatory pathway for DNA vaccine + adjuvant on MN patches; broader clinical evidence for oncologic indications is pending	Cancer immunotherapy (DNA vaccine) via transcutaneous delivery with enhanced DC activation, type-I IFN, antibodies & $CD8^+$ T-cells	69
	Microchannel-based system (2018)	TRL 3–4	Geometric control of release rates, pH-responsive function verified with cell viability assays, all *in vitro* studies	Lacks the evidence of *in vivo* studies, needs biocompatibility studies and scale-up of microfabrication and regulatory path for channel-programmed,	Programmable multi-phase delivery of therapeutics in *in vitro* settings, foundational architecture for future implants	70

				multi-payload implants remain open		
	Microrocket (2019)	TRL 4–5	*In vivo* animal studies show enhanced local drug retention, magnetic targeting, and biodegradability of PASP/Fe–Zn components, supporting SI details zeta-potential, propulsion, and assay methods	Scale-up and manufacturability of uniform microrockets, navigation and imaging in complex gastric dynamics, long-term biocompatibility & by-product fate (Zn^{2+}/Fe species) in larger animals, regulatory path for self-propelled ingestible micromotors remains undefined	Targeted gastric delivery elevated mucosal retention	71
	Microneedle (2021)	TRL 3-4	pH-responsive microneedle systems have been successfully demonstrated *in vivo* in animal models, showing	Requires validation in infection-bearing wound models,	Smart wound healing—on-demand release of	72

			microenvironment-responsive release, sustained therapeutic effects, and safe skin insertion. Evidence from analogous pH-responsive core-shell MNs shows environment-triggered rupture and prolonged drug action in rodent models	long-term safety of polymer coatings, and stability of pH-responsive performance in heterogeneous wound fluids, challenges in scaling uniform polymer coatings, sterility assurance, and regulatory classification as an adaptive wound-responsive medical device	antimicrobial/anti-inflammatory/analgesic agents in response to wound acidity, supports chronic wound management and skin-barrier repair	
	Asymmetric microfluidic/chitosan pH-responsive device (2023)	TRL 3–4	Demonstrated pH-dependent release and antibacterial performance *in vitro*, biocompatibility assessed, and initial *ex vivo* bone-mimetic testing	No *in vivo* bone defect validation, limited data on osteointegration, sterilization and mechanical robustness	Local drug release for infective bone defect treatment	73

				issues, manufacturing scalability, and regulatory challenges for implantable drug-release devices		
	Double-layered MOF-based micro swimmers (2024)	TRL 2–3	*In vitro* propulsion, ROS-triggered dual-drug release, and cytotoxicity assays, no *in vivo* tumor studies	Poor propulsion in physiological conditions, potential metal toxicity, unclear clearance pathway, scale-up barriers, and regulatory uncertainty	Adaptive dual-drug anti-cancer therapy	74

	Microneedle (2024)	TRL 4–5	*In vivo* validation in rat postoperative pain model: MNs implant in skin within 15 min, pH-responsive shell rupture, prolonged ropivacaine release >72 h, significant reduction in mechanical & thermal pain sensitivity	Requires large-animal studies, long-term biocompatibility, and safety of microcrystal release kinetics; manufacturing scale-up of core–shell MNs, regulatory pathways for responsive MN analgesic systems not yet defined, need to validate self-monitoring accuracy across diverse wound types	Postoperative analgesia with >72 h sustained pain relief, responsive to incision microenvironment; minimally invasive local delivery	75

Enzyme	Shape-coded hydrogel microparticle-based multiplexed enzyme bioassay in a microfluidic device (2010)	TRL 2–3	Demonstrated shape-coded hydrogel fabrication, enzyme immobilization, and simultaneous multi-analyte detection *in vitro*, compatible with microfluidic flow-based analysis; similar to other immobilized multi-enzyme microreactor systems used for analytical research	No clinical or *in vivo* relevance, enzyme stability limitations, mass-manufacturing of encoded particles, need for integration with validated clinical diagnostic workflows, and regulatory irrelevance for therapeutic translation	Multiplexed biochemical sensing, diagnostic assays, high-throughput screening	76
	Microfluidic device (2012)	TRL 2–3	Demonstrated shape-coded hydrogel fabrication, enzyme immobilization, and simultaneous multi-analyte detection *in vitro*, compatible with microfluidic flow-based analysis; similar to other immobilized multi-enzyme microreactor systems used for analytical research	No clinical or *in vivo* relevance, enzyme stability limitations, mass-manufacturing of encoded particles, need for integration with validated clinical diagnostic workflows, and regulatory irrelevance for	Multiplexed biochemical sensing, diagnostic assays, high-throughput screening	77

				therapeutic translation		
	Microneedles (2016)	TRL 4–5	Demonstrated enhanced immune activation *in vitro* and *in vivo*, validated antitumor response with synergistic immunotherapy	Human skin penetration variability, dose-scaling for checkpoint inhibitors, safety and immune-related adverse event risk, GMP manufacturing of delivery system, regulatory hurdles for combination	Antitumor immunotherapy via transdermal delivery of checkpoint inhibitors	78
	Catalase-laden microdevice (2016)	TRL 3–4	Demonstrated microdevice loading and stable attachment to cells, preserved cell viability, and enzyme activity *in vitro*, comparable systems show *in vivo* effects in rodents, supporting similar maturity levels	Needs controlled *in vivo* biodistribution, enzyme longevity, immunogenicity assessment, scalable fabrication, and regulatory definition for enzyme-device + living-cell	Antioxidant therapy, inflammation modulation, and cell-assisted enzyme delivery	79

				combinational products		
	Actuator (2024)	TRL 3	Demonstrated enzyme-triggered expansion, morphological change, and controlled release *in vitro*; no *in vivo* testing	Enzyme availability varies physiologically, with limited stability in complex biological fluids, scale-up of protein-PEGDA hybrid hydrogels, lack of biocompatibility and safety data for *in vivo* environments	Controlled drug release + shape-morphing hydrogel actuator	80

	Microrobotic enzyme-responsive hydrogel delivery system (2025)	TRL 3–4	Demonstrated targeted microrobotic navigation, enzyme-triggered drug release, and significant tumour suppression *in vitro* and *in vivo* studies	Challenges in deep-tissue microrobot navigation, enzyme variability between human tumours, long-term biocompatibility of microrobots, scale-up manufacturing, and complex regulatory pathway for combined micro-robotic therapeutic systems	Localized delivery of hydrogel-immobilized therapeutics for triple-negative breast cancer	81
Redox	Closed-loop drug-level control system (biosensor + controller + infusion pump) (2017)	TRL 5	Demonstrated in live rabbits and rats: continuous measurement of doxorubicin levels and real-time dose adjustments; system maintained desired set-points despite inter-animal PK variability and acute drug–drug interactions	Requires miniaturization for human use, long-term biosensor stability, biocompatibility validation, clinical-grade pump integration,	Precision dosing of chemotherapeutics, patient-specific pharmacokinetic compensation	82

				cybersecurity/algorithm robustness, and a regulatory framework for automated closed-loop therapeutic systems		
	DNA-crosslinked, donor–acceptor (D–A) stimuli-responsive CMC hydrogels (2018)	TRL 2–3	Reversible high/low stiffness via D–A or G-quadruplex switching, shape-memory & triggered self-healing demonstrated *in vitro*	Translation would require biostability in physiological fluids, enzymatic degradation control of DNA linkers, scalable synthesis & sterilization, and use-case/regulatory pathway definition	Smart functional hydrogel matrices for biosensing/actuation research	83
	Miniaturized redox-capacitor assembled inside a microdevice (2018)	TRL 2-3	Demonstrated selective assembly, surface functionalization, and electrochemical performance *in vitro*, supports toxin sensing and cell-response quantification in microfluidic environments,	Lack of *in vivo* studies requires enhanced stability, antifouling properties, high-throughput	Microbial toxin detection, mammalian cell cytotoxicity analysis, redox-based microfluidic bioassays	84

			comparable to other analytical microdevices such as spectro-electrochemical platforms and immobilized-enzyme microreactors	integration, standardized calibration, and no defined regulatory pathway for use beyond laboratory diagnostics		
	Microneedle (2021)	TRL 4	Demonstrated microneedle delivery, TME-triggered MIL-100(Fe) degradation, O_2 generation, and enhanced chemo-phototherapy *in vitro* and *in vivo* studies	Limited human skin penetration variability, uncertain Fe-based MOF metabolism/clearance, potential photothermal safety concerns, scale-up of MN-MOF integration, and regulatory barriers for combination nanomedicine-microneedle systems	O_2-evolving chemophototherapy for solid tumors	[85]
	Organohydrogel-	TRL 2–3	3D-stretchable devices (up to 500%	No *in vivo*/clinical	Stretchable	[86]

	based soft electronics with polyphenol-incorporated double networks (2022)		strain), >1000 cycles at 200% strain with preserved performance, >200 $mF \cdot cm^{-2}$ capacitance; multi-component (TA/EG) gels enabling robust interfaces, all demonstrated *in vitro*/lab-scale prototypes	data, long-term biocompatibility and sweat/skin environment stability; sterilization and packaging, large-area manufacturing and device encapsulation, regulatory path for wearable bioelectronic materials remains to be defined	bioelectronics for wearable/skin-like devices	

	Wireless bioelectronic device (2024)	TRL 4–5	Demonstrated controlled electrochemical release, wireless programmability, and significant tumour suppression *in vivo* studies	Long-term biocompatibility of implanted electronics, risk of electrochemical byproducts, complex regulatory path for active implantable, manufacturing scalability, and human-scale intratumoral dosing challenges	Localized, programmable intratumoral drug release for breast cancer	87
	4D-printed redox-responsive needle panel meter (2025)	TRL 2–3	Demonstrated programmable 4D-printed geometric deformation under redox conditions; validated glucose and lactate sensing *in vitro* studies, lack of biological or clinical validation	Lacks the long-term clinical validation, requires manual enzymatic steps, needs microfluidic integration, sample preprocessing required,	Quantitative sensing of glucose and lactate concentrations	88

				lab-optimized conditions are unlikely to translate directly to field use, no continuous wear/biocompatibility data, and not benchmarked against clinical CGM standards		
	3D-printed spectro-electrochemical platform for redox-based bioelectronic device (2025)	TRL 2–3	Demonstrated 3D-printed microdevice fabrication, integrated electrode–optical detection, and redox signal characterization *in vitro*, typical of spectro-electrochemical microdevices designed for analytical testing	Not clinically oriented, requires improved sensitivity, electrode stability, biofouling resistance, standardized calibration, and integration with validated clinical	Biosensing, redox-based biochemical analysis, lab-on-chip bioelectronics	89

				diagnostic workflows		
Hypoxia	Microfluidic device generating tunable oxygen gradients (2015)	TRL 3	Similar hypoxia-engineered platforms demonstrate controlled oxygen gradients and 3D hypoxic microenvironments for studying cancer and endothelial cell responses *in vitro* and *in vivo*	No *in vivo* validation; microdevice does not replicate full tumor microenvironment (ECM, immune cells, perfusion); challenges in scaling for drug-screening pipelines, regulatory irrelevance for therapeutic development	*In vitro* evaluation of hypoxia-dependent cytotoxicity of anticancer drugs, cancer microenvironment modeling	90
	Microdevice platform recapitulating hypoxic tumor microenvironments (2017)	TRL 2–3	Demonstrated formation of physiologically relevant oxygen gradients, validated with oxygen sensor imaging, numerical modeling, and spatially resolved hypoxic signaling in cancer cells, entirely *in vitro* prototype research	Not intended for *in vivo* use, lacks physiological complexity, challenges in scaling, standardization, and integration with	*In vitro* modeling of tumor hypoxia, signaling studies, gradient mapping, and drug-response assays	91

				regulated diagnostic workflows, and regulatory path irrelevant to clinical therapeutics because it is an analytical research tool		
	O_2-controllable laccase-crosslinked gelatin hydrogel (2017)	TRL 4	Platform validated through polymer synthesis, O_2-monitoring assays, rheology, and 3D cell encapsulation, shown to control hypoxia gradients in standard culture and rodent *in vivo* systems	Requires biostability optimization for long-term implantation; limited by host-immune response, diffusion-limited nutrient transport, scalability for therapeutic use, and no regulatory pathway	Studying hypoxia-driven cell behaviour *in vitro*, evaluating tissue grafts & tumor models *in vivo*	92

	High-throughput microfluidic system with linear oxygen gradient (2021)	TRL 3–4	Demonstrated high-throughput spheroid generation and exposure to five distinct oxygen levels: quantified ROS and drug responses (doxorubicin, tirapazamine) in breast tumor spheroids *in vitro*	Not suitable for *in vivo* use, limited to *in vitro* modelling, device fabrication complexity, integration challenges for clinical workflows, and no regulatory pathway since it is a research-only microfluidic platform	Study of chronic/cyclic hypoxia, ROS generation, and drug cytotoxicity in tumor spheroids	93
	Oxygen-tunable endothelialized microvascular chip (2025)	TRL 3–4	Demonstrated precise control of oxygen levels over endothelialized microchannels, enabling hypoxia–reperfusion cycles *in vitro*, consistent with other validated microfluidic oxygen-control systems used for tumor and disease modeling	Limited to *in vitro* disease modelling, lacks systemic circulation, immune components, and hemodynamic complexity, and fabrication challenges for large-scale use	Modeling hypoxia–reperfusion injury and vaso-occlusion in sickle cell disease	94
Ionic	Salt-responsive	TRL 2–3	Demonstrated reversible salt-induced	Not validated in	Antifouling coatings,	95

	zwitterionic polymer brushes (2015)		swelling, lubrication switching, and reduced protein/cell adsorption *in vitro,* consistent with known zwitterionic brush behaviour and responsive polymer-surface engineering literature	complex biological environments; long-term stability under physiological ionic strengths unresolved, scale-up and substrate-compatibility challenges; regulatory pathway undefined	tunable-friction surfaces, biosensing and marine/medical surface protection	
	Hydrogel (2017)	TRL 3–4	Analogous multi-stimuli hydrogels demonstrate *in vitro* responsiveness and occasional rodent-model validation of microenvironment-triggered behaviour, supporting similar developmental maturity	No standardized *in vivo* pharmacokinetics, enzymatic response varies across tissues; need for GMP-grade polypeptide synthesis; mechanical stability and sterilization challenges; regulatory uncertainty for multi-	Site-specific drug delivery in pathological microenvironments with simultaneous ionic/pH/enzymatic cues	96

				trigger, biodegradable peptide hydrogels		
	Degradable soft ionic microdevices (2020)	TRL 2–3	Demonstrated stretchability, ionic conductivity, rapid self-healing, and controlled degradation *in vitro*, device prototypes and basic electromechanical performance shown in lab conditions	No *in vivo* validation, long-term biocompatibility and degradation-product safety unknown; challenges in sterilization, integration with electronics, and scaling of organohydrogel fabrication, unclear regulatory pathway for degradable soft-ionic implantable	Soft bioelectronics, wearable/implantable sensing, soft robotic microdevices	97
	Zwitterionic polymer-brush (2020)	TRL 2–3	Demonstrated synthetic grafting of zwitterionic brushes onto hydrogel matrices, ion-responsive friction	No validation under physiological shear/ionic	Low-friction, antifouling, and tunable-lubrication	98

			coefficients measured *in vitro*, behaviour consistent with other zwitterionic salt-responsive polymer brush studies	complexity, long-term stability in biological fluids uncertain; scaling of brush-hydrogel grafting, no regulatory framework	hydrogel coatings, biomaterial surface engineering	
	Microneedles (2023)	TRL 4–5	Fabrication by template replication + 3D transfer printing, ion-tunable mechanics demonstrated, *in vivo* efficacy in diabetic rat full-thickness wounds with enhanced healing/regeneration	Need GMP-grade exosomes and batch consistency, long-term safety of indwelling tips, sterilization & shelf-life, human-skin adhesion/retention variability, combo-product regulatory path (device + biologic)	Diabetic ulcer healing (pro-angiogenic, immunomodulatory exosome delivery)	99
	Agar/alginate wet-spun microfiber-shaped	TRL 2-3	Typical agar/alginate wet-spun microfibers show controllable mechanical behaviour, ionic responsiveness, and	No stability/safety data under physiological pH,	Soft actuators, micro-scaffolds, and responsive microfiber	100

	hydrogel (2023)		microscale patterning, used strictly *in vitro* as functional hydrogel materials	complex degradation behaviour, need for reproducible micro-topographic manufacturing, no regulatory framework, and a lack of *in vivo* studies	platforms for sensing or cell-guidance	
	Ionic-liquid–mediated organogel for Cyclosporine A transdermal delivery (2024)	TRL 4–5	Comparable hydrogel and organogel systems have been validated *in vitro* and *in vivo* in rodent models, demonstrating controlled microenvironment-responsive behaviour and effective delivery under physiological conditions	Requires long-term dermal toxicity studies, variability of ionic liquid safety across species, need for human skin permeation validation, organogel scalability and stability, and regulatory ambiguity for ionic-liquids enhanced pharmaceutical	Topical treatment of psoriasis via localized Cyclosporine A delivery without systemic toxicity	101

				formulations		
	Microgels (2025)	TRL 2–3	Demonstrated ion-triggered crosslinking, tunable rheology, printability, and shape-changing behaviour *in vitro* studies, similar to other early-stage microfluidic and oxygen-controlled biofabrication platforms, used only in lab environments	No biological validation; long-term stability unknown, scaling issues for manufacturing, lack of biocompatibility testing *in vivo*, unclear regulatory pathway for printed smart materials	3D and 4D (bio)printing for dynamic constructs, shape-morphing materials	102

TRL 1, 2: Basic principles observed, technology concept formulated**; TRL 3:** Experimental proof of concept shown in lab**; TRL 4:** Laboratory validation of component/process**; TRL 5:** Validation in relevant biological models**; TRL 6:** Prototype demonstration in preclinical/ GLP studies**; TRL 7:** Prototype demonstration in human clinical trials Phase I**; TRL 8:** Technology qualified and approval (regulatory clearance)**; TRL 9:** Actual system proven in routine clinical/ market use

References

1 B. J. Lee, K. Hsiao, G. Lipkowitz, T. Samuelsen, L. Tate and J. M. DeSimone, *Addit. Manuf.*, 2022, **55**, 102800.

2 C. T. Hagan, C. Bloomquist, I. Kim, N. M. Knape, J. D. Byrne, L. Tu, K. Wagner, S. Mecham, J. DeSimone and A. Z. Wang, *Acta Biomater.*, 2022, **148**, 163–170.

3 R. Janusziewicz, J. R. Tumbleston, A. L. Quintanilla, S. J. Mecham and J. M. DeSimone, *Proceedings of the National Academy of Sciences*, 2016, **113**, 11703–11708.

4 S. R. Dabbagh, M. R. Sarabi, M. T. Birtek, S. Seyfi, M. Sitti and S. Tasoglu, *Nat. Commun.*, 2022, **13**, 1–24.

5 F. M. den Hoed, M. Carlotti, S. Palagi, P. Raffa and V. Mattoli, *Micromachines 2024, Vol. 15, Page 275*, 2024, **15**, 275.

6 A. Das, A. Ghosh, S. Chattopadhyaya and C. F. Ding, *Opt. Laser Technol.*, 2024, **168**, 109893.

7 J. Li and M. Pumera, *Chem. Soc. Rev.*, 2021, **50**, 2794–2838.

8 I. Xenikakis, M. Tzimtzimis, K. Tsongas, D. Andreadis, E. Demiri, D. Tzetzis and D. G. Fatouros, *European Journal of Pharmaceutical Sciences*, 2019, **137**, 104976.

9 F. P. W. Melchels, J. Feijen and D. W. Grijpma, *Biomaterials*, 2010, **31**, 6121–6130.

10 T. D. Ngo, A. Kashani, G. Imbalzano, K. T. Q. Nguyen and D. Hui, *Compos. B Eng.*, 2018, **143**, 172–196.

11 T. M. Valentin, S. E. Leggett, P. Y. Chen, J. K. Sodhi, L. H. Stephens, H. D. McClintock, J. Y. Sim and I. Y. Wong, *Lab Chip*, 2017, **17**, 3474–3488.

12 J. Ziesmer, I. Sondén, J. Venckute Larsson, P. Merkl and G. A. Sotiriou, *ACS Appl. Bio Mater.*, 2024, **7**, 4533–4541.

13 X. Wang, J. Liu, Y. Zhang, P. M. Kristiansen, A. Islam, M. Gilchrist and N. Zhang, *Virtual Phys. Prototyp.*, DOI:10.1080/17452759.2023.2248101.

14 I. Ertugrul, *Micromachines 2020, Vol. 11, Page 518*, 2020, **11**, 518.

15 Z. Luo, H. Zhang, R. Chen, H. Li, F. Cheng, L. Zhang, J. Liu, T. Kong, Y. Zhang and H. Wang, *Microsyst. Nanoeng.*, 2023, **9**, 1–13.

16 S. B. Gugulothu and K. Chatterjee, *ACS Macro Lett.*, 2023, **12**, 494–502.

17 M. Carlotti, O. Tricinci and V. Mattoli, *Adv. Mater. Technol.*, 2022, **7**, 2101590.

18 W. Wang, Z. Q. Chen, B. Lin, M. C. Liu, Y. Zhang, S. J. Liu, Y. Li and Q. Zhao, *Chemical Engineering Journal*, 2024, **493**, 152469.

19 T. Zandrini, S. Taniguchi and S. Maruo, *Micromachines 2017, Vol. 8, Page 35*, 2017, **8**, 35.

20 S. Pagliano, D. E. Marschner, D. Maillard, N. Ehrmann, G. Stemme, S. Braun, L. G. Villanueva and F. Niklaus, *Microsyst. Nanoeng.*, 2022, **8**, 105.

21 A. Isaakidou, I. Apachitei, L. E. Fratila-Apachitei and A. A. Zadpoor, *Journal of Functional Biomaterials 2023, Vol. 14, Page 494*, 2023, **14**, 494.

22 E. O. Olakanmi, R. F. Cochrane and K. W. Dalgarno, *Prog. Mater. Sci.*, 2015, **74**, 401–477.

23 P. Kulinowski, P. Malczewski, E. Pesta, M. Łaszcz, A. Mendyk, S. Polak and P. Dorożyński, *Addit. Manuf.*, 2021, **38**, 101761.

24 S. R. Dabbagh, M. R. Sarabi, R. Rahbarghazi, E. Sokullu, A. K. Yetisen and S. Tasoglu, *iScience*, 2021, **24**, 102012.

25 S. Mei, Z. Li, X. Chen, W. Zhao, Y. Zhang, X. Zhang, Z. Cui, P. Fu, X. Pang and M. Liu, *Journal of Materials Research and Technology*, 2023, **26**, 5095–5104.

26 S. C. Ligon, R. Liska, J. Stampfl, M. Gurr and R. Mülhaupt, *Chem. Rev.*, 2017, **117**, 10212–10290.

27 M. O. F. Emon, F. Alkadi, D. G. Philip, D. H. Kim, K. C. Lee and J. W. Choi, *Addit. Manuf.*, 2019, **28**, 629–638.

28 C. Lin, L. Liu, Y. Liu and J. Leng, *ACS Appl. Mater. Interfaces*, 2021, **13**, 12668–12678.

29 S. Mousavi, D. Howard, F. Zhang, J. Leng and C. H. Wang, *ACS Appl. Mater. Interfaces*, 2020, **12**, 15631–15643.

30 X. Zhang, H. Wei, C. Dong, J. Wang, T. Zhang, L. Huang, D. Ni and Y. Luo, *Chemical Engineering Journal*, 2023, **461**, 141855.

31 N. M. Elman, Y. Patta, A. W. Scott, B. Masi, H. L. Ho Duc and M. J. Cima, *Clin. Pharmacol. Ther.*, 2009, **85**, 544–547.

32 M. A. Moses, H. Brem and R. Langer, *Cancer Cell*, 2003, **4**, 337–341.

33 A. Baldi, M. Lei, Y. Gu, R. A. Siegel and B. Ziaie, *Sens. Actuators B Chem.*, 2006, **114**, 9–18.

34 E. Ginter and V. Simko, *Adv. Exp. Med. Biol.*, 2013, **771**, 42–50.

35 A. C. Richards Grayson, I. S. Choi, B. M. Tyler, P. P. Wang, H. Brem, M. J. Cima and R. Langer, *Nat. Mater.*, 2003, **2**, 767–772.

36 Where a Pill Won't Reach on JSTOR, https://www.jstor.org/stable/26060243, (accessed 25 October 2025).

37 J. T. Santini, M. J. Cima and R. Langer, *Nature*, 1999, **397**, 335–338.

38 F. Frankel, DOI:10.1002/1521-3773(20000717)39:14.

39 Y. Li, R. S. Shawgo, B. Tyler, P. T. Henderson, J. S. Vogel, A. Rosenberg, P. B. Storm, R. Langer, H. Brem and M. J. Cima, *Journal of Controlled Release*, 2004, **100**, 211–219.

40 A. Altuna, E. Bellistri, E. Cid, P. Aivar, B. Gal, J. Berganzo, G. Gabriel, A. Guimerà, R. Villa, L. J. Fernández{ Ef and L. Menendez De La Prida{, 2013, **13**, 1422.

41 K. Gao, G. Li, L. Liao, J. Cheng, J. Zhao and Y. Xu, *Sens. Actuators A Phys.*, 2013, **197**, 9–14.

42 Y. Zhong and R. V. Bellamkonda, *Brain Res.*, 2007, **1148**, 15–27.

43 S. T. Retterer, K. L. Smith, C. S. Bjornsson, K. B. Neeves, A. J. H. Spence, J. N. Turner, W. Shain and M. S. Isaacson, *IEEE Trans. Biomed. Eng.*, 2004, **51**, 2063–2073.

44 B. C. Masi, B. M. Tyler, H. Bow, R. T. Wicks, Y. Xue, H. Brem, R. Langer and M. J. Cima, *Biomaterials*, 2012, **33**, 5768–5775.

45 G. alias R. R. Naik, R. S. P, S. R. Jadhav, R. Pokale, P. Hedayat, D. Datta, B. Prajapati, S. Mutalik and N. Dhas, *ACS Pharmacol. Transl. Sci.*, 2025, **2025**, 3410.

46 Panigrahi M, D. Pk and P. Pm, *Indian J. Cancer*, DOI:10.4103/0019-509X.76623.

47 R. Farra, N. F. Sheppard, L. McCabe, R. M. Neer, J. M. Anderson, J. T. Santini, M. J. Cima and R. Langer, *Sci. Transl. Med.*, DOI:10.1126/SCITRANSLMED.3003276.

48 N. M. Elman, H. L. Ho Duc and M. J. Cima, *Biomed. Microdevices*, 2009, **11**, 625–631.

49 F. N. Pirmoradi, J. Jackson, H. Burt and M. Chiao, *2011 16th International Solid-State Sensors, Actuators and Microsystems Conference, TRANSDUCERS'11*, 2011, 2831–2834.

50 J. B. Wolinsky, Y. L. Colson and M. W. Grinstaff, *Journal of Controlled Release*, 2012, **159**, 14–26.

51 S. Alken and C. M. Kelly, *Cancer Manag. Res.*, 2013, **5**, 357–365.

52 E. E. L. Pararas, D. A. Borkholder and J. T. Borenstein, *Adv. Drug Deliv. Rev.*, 2012, **64**, 1650–1660.

53 J. Fiering, M. J. Mescher, E. E. Leary Swan, M. E. Holmboe, B. A. Murphy, Z. Chen, M. Peppi, W. F. Sewell, M. J. McKenna, S. G. Kujawa and J. T. Borenstein, *Biomed. Microdevices*, 2009, **11**, 571–578.

54 Otology & Neurotology, https://journals.lww.com/otology-neurotology/citation/1985/03000/Cochlear_Implants__1981_To_1985.13.aspx, (accessed 25 October 2025).

55 C. L. Budenz, B. E. Pfingst and Y. Raphael, *The Anatomical Record: Advances in Integrative Anatomy and Evolutionary Biology*, 2012, **295**, 1896–1908.

56 L. Meng, Z. Deng, L. Niu, F. Li, F. Yan, J. Wu, F. Cai and H. Zheng, *Theranostics*, 2015, **5**, 1203–1213.

57 V. Cricchio, M. Best, E. Reverchon, N. Maffulli, G. Phillips, M. Santin and G. Della Porta, *J. Pharm. Sci.*, 2017, **106**, 2097–2105.

58 H. Lee, H. Choi, M. Lee and S. Park, *Biomed. Microdevices*, DOI:10.1007/s10544-018-0344-y.

59 Y. H. Roh, J. Y. Moon, E. J. Hong, H. U. Kim, M. S. Shim and K. W. Bong, *Colloids Surf. B Biointerfaces*, 2018, **172**, 380–386.

60 L. D'eramo, B. Chollet, M. Leman, E. Martwong, M. Li, H. Geisler, J. Dupire, M. Kerdraon, C. Vergne, F. Monti, Y. Tran and P. Tabeling, *Microsystems & Nanoengineering 2018 4:1*, 2018, **4**, 17069-.

61 J. Xia, A. C. Tsai, W. Cheng, X. Yuan, T. Ma and J. Guan, *Biomater. Sci.*, 2019, **7**, 2348–2357.

62 A. Ghosh, L. Li, L. Xu, R. P. Dash, N. Gupta, J. Lam, Q. Jin, V. Akshintala, G. Pahapale, W. Liu, A. Sarkar, R. Rais, D. H. Gracias and F. M. Selaru, *Sci. Adv.*, 2020, **6**, 4133–4161.

63 J. Y. Li, Y. H. Feng, Y. T. He, L. F. Hu, L. Liang, Z. Q. Zhao, B. Z. Chen and X. D. Guo, *Acta Biomater.*, 2022, **153**, 308–319.

64 Y. Zhou, M. Ye, H. Zhao and X. Wang, *Int. J. Bioprint.*, 2023, **9**, 709.

65 J. Chen, M. Chu, K. Koulajian, X. Y. Wu, A. Giacca and Y. Sun, *TRANSDUCERS 2009 - 15th International Conference on Solid-State Sensors, Actuators and Microsystems*, 2009, 1465–1468.

66 D. S. Vanblarcom and N. A. Peppas, *Biomedical Microdevices 2011 13:5*, 2011, **13**, 829–836.

67 C. J. Ke, Y. J. Lin, Y. C. Hu, W. L. Chiang, K. J. Chen, W. C. Yang, H. L. Liu, C. C. Fu and H. W. Sung, *Biomaterials*, 2012, **33**, 5156–5165.

68 H. Li, G. Go, S. Y. Ko, J. O. Park and S. Park, *Smart Mater. Struct.*, 2016, **25**, 027001.

69 H. T. T. Duong, Y. Yin, T. Thambi, T. L. Nguyen, V. H. Giang Phan, M. S. Lee, J. E. Lee, J. Kim, J. H. Jeong and D. S. Lee, *Biomaterials*, 2018, **185**, 13–24.

70 D. Yang, J. S. Lee, C. K. Choi, H. P. Lee, S. W. Cho and W. H. Ryu, *Acta Biomater.*, 2018, **68**, 249–260.

71 M. Zhou, T. Hou, J. Li, S. Yu, Z. Xu, M. Yin, J. Wang and X. Wang, *ACS Nano*, 2019, **13**, 1324–1332.

72 A. Ullah, M. Jang, H. Khan, H. J. Choi, S. An, D. Kim, Y. R. Kim, U. K. Kim and G. M. Kim, *Sensors and Actuators B-chemical*, DOI:10.1016/j.snb.2021.130441.

73 H. Chen, W. Tan, T. Tong, X. Shi, S. Ma and G. Zhu, *Int. J. Mol. Sci.*, DOI:10.3390/ijms24054616.

74 Y. Zhong, H. Li, T. Jiang, X. Mu, M. Seki and U. K. Cheang, *Advanced Intelligent Systems*, 2024, **6**, 2400244.

75 A. Zhang, Y. Zeng, B. Xiong, X. Jiang, Y. Jin, S. Wang, Y. Yuan, W. Li and M. Peng, *Adv. Funct. Mater.*, 2024, **34**, 2314048.

76 E. Jang and W. G. Koh, *Sens. Actuators B Chem.*, 2010, **143**, 681–688.

77 M. K. L. Chu, J. Chen, C. R. Gordijo, S. Chiang, A. Ivovic, K. Koulajian, A. Giacca, X. Y. Wu and Y. Sun, *Lab Chip*, 2012, **12**, 2533–2539.

78 Y. Ye, J. Wang, Q. Hu, G. M. Hochu, H. Xin, C. Wang and Z. Gu, *ACS Nano*, 2016, **10**, 8956–8963.

79 J. Xia, Z. Wang, Y. Yan, Z. Cheng, L. Sun, Y. Li, Y. Ren and J. Guan, *Langmuir*, 2016, **32**, 13386–13393.

80 Y. Liu and L. R. Khoury, *Small science*, DOI:10.1002/smsc.202400214.

81 M. Tian, M. Keshavarz, A. A. Demircali, B. Han and G. Z. Yang, *Small*, DOI:10.1002/smll.202408813.

82 P. L. Mage, B. S. Ferguson, D. Maliniak, K. L. Ploense, T. E. Kippin and H. T. Soh, *Nature Biomedical Engineering 2017 1:5*, 2017, **1**, 0070-.

83 C. Wang, M. Fadeev, M. Vázquez-González and I. Willner, *Adv. Funct. Mater.*, 2018, **28**, 1803111.

84 W. Shang, Y. Liu, E. Kim, C. Y. Tsao, G. F. Payne and W. E. Bentley, *Lab Chip*, 2018, **18**, 3578–3587.

85 S. Luo, Y. Zhao, K. Pan, Y. Zhou, G. Quan, X. Wen, X. Pan and C. Wu, *Biomater. Sci.*, 2021, **9**, 6772–6786.

86 W. Wang, F. Chen, L. Fang, Z. Li and Z. Xie, *ACS Appl. Mater. Interfaces*, 2022, **14**, 12583–12595.

87 M. Souri, S. Elahi and M. Soltani, *Expert Opin. Drug Deliv.*, 2024, **21**, 495–511.

88 T. H. Yang, Y. S. Birhan, P. C. Chu, L. R. Chanu and P. S. Lai, *Mater. Des.*, DOI:10.1016/j.matdes.2025.114228.

89 C. Y. Chen, E. Kim, F. R. Zakaria, M. J. Chu, B. Wu, G. F. Payne and W. E. Bentley, *Small Methods*, 2025, **9**, 2401843.

90 Z. Wang, Z. Liu, L. Li and Q. Liang, *Microfluidics and Nanofluidics 2015 19:6*, 2015, **19**, 1271–1279.

91 Y. Ando, H. P. Ta, D. P. Yen, S. S. Lee, S. Raola and K. Shen, *Scientific Reports 2017 7:1*, 2017, **7**, 15233-.

92 D. M. Lewis, M. R. Blatchley, K. M. Park and S. Gerecht, *Nature Protocols 2017 12:8*, 2017, **12**, 1620–1638.

93 R. Primavera, M. Razavi, B. D. Kevadiya, Y. Zhang, F. Jiang, Y. Charles Zhao, al -, S. Flores-Torres, O. Peza-Chavez, H. Kuasne, I. Berger Fridman, G. Stefano Ugolini, V. VanDelinder, S. Cohen and T. Konry, *Biofabrication*, 2021, **13**, 035037.

94 S. R. Schad, J. D. Beckman, W. A. Lam and D. K. Wood, *Lab Chip*, 2025, **25**, 4920–4933.

95 J. Yang, H. Chen, S. Xiao, M. Shen, F. Chen, P. Fan, M. Zhong and J. Zheng, *Langmuir*, 2015, **31**, 9125–9133.

96 G. Ma, W. Lin, Z. Yuan, J. Wu, H. Qian, L. Xu and S. Chen, *J. Mater. Chem. B*, 2017, **5**, 935–943.

97 L. Fang, J. Zhang, W. Wang, Y. Zhang, F. Chen, J. Zhou, F. Chen, R. Li, X. Zhou and Z. Xie, *ACS Appl. Mater. Interfaces*, 2020, **12**, 56393–56402.

98 A. O. Osaheni, A. Ash-Shakoor, I. Gitsov, P. T. Mather and M. M. Blum, *Langmuir*, 2020, **36**, 3932–3940.

99 X. Zhang, J. Gan, L. Fan, Z. Luo and Y. Zhao, *Advanced Materials*, 2023, **35**, 2210903.

100 W. Yekai, L. Haoran, Y. Kun, L. Xiufang, L. Zhentan and W. Dong, *Carbohydr. Polym.*

101 D. Datta, S. P. Bandi and V. V. K. Venuganti, *ACS Omega*, 2024, **9**, 41565–41582.

102 V. Pal, D. Gupta, S. Liu, I. Namli, S. H. A. Rizvi, Y. O. Yilmaz, L. Haugh, E. M. Gerhard and I. T. Ozbolat, *Small*, 2025, **21**, e02262.
